# Analytical Prediction of Critical Transitions in Oscillator Networks With Non‐Local Links

**DOI:** 10.1002/advs.78134

**Published:** 2026-10-08

**Authors:** Qiang Li, Chumin Sun, David Waxman, Renaud Lambiotte, Jie Sun, Peng Ji

**Affiliations:** ^1^ Institute of Science and Technology for Brain‐Inspired Intelligence Fudan University Shanghai China; ^2^ College of Future Information Technology Fudan University Shanghai China; ^3^ Theory Lab, Central Research Institute, 2012 Labs Huawei Technologies Co., Ltd. Sha Tin Hong Kong; ^4^ Key Laboratory of Computational Neuroscience and Brain‐Inspired Intelligence Ministry of Education Shanghai China; ^5^ Mathematical Institute University of Oxford Oxford UK; ^6^ MOE Frontiers Center for Brain Science Fudan University Shanghai China

**Keywords:** complex network, dynamical system, dynamical systems theory, kuramoto model, statistical physics

## Abstract

A wide range of dynamical systems can be modeled as oscillators coupled through an interaction network. Such frameworks have been widely used to explore the emergence of collective phenomena across diverse applications. Here, we develop an analytical framework for oscillator‐based Ising systems with Kuramoto‐type pairwise coupling and a second harmonic term. Spectral dimension reduction identifies a stability boundary governed by the balance between global coupling and second‐harmonic strength, while a mean‐field Fokker–Planck analysis predicts the transition between single‐cluster and two‐cluster stationary states and clarifies the role of noise. Low‐energy Ising configurations are predominantly obtained when the second harmonic term is strong relative to noise. Partition‐function analysis further shows that, for the ring‐based topology, links to third neighbors yield more desired solutions than the other added non‐local connections. Applications to graph coloring and MaxCut demonstrate the relevance of these predictions to oscillator‐based Ising optimization. These results provide a predictive framework for understanding how dynamical parameters, noise, and network structure jointly shape collective states and low‐energy configurations.

## Graphical abstract

An analytical framework reveals how global coupling, second‐harmonic strength, noise, and network structure govern oscillator‐based Ising dynamics. Spectral reduction predicts stability boundaries, while Fokker–Planck analysis captures one‐ to two‐cluster transitions and identifies conditions favoring low‐energy configurations. For the ring‐based topology, third‐neighbor links yield more desired solutions, with implications demonstrated through graph coloring and MaxCut.

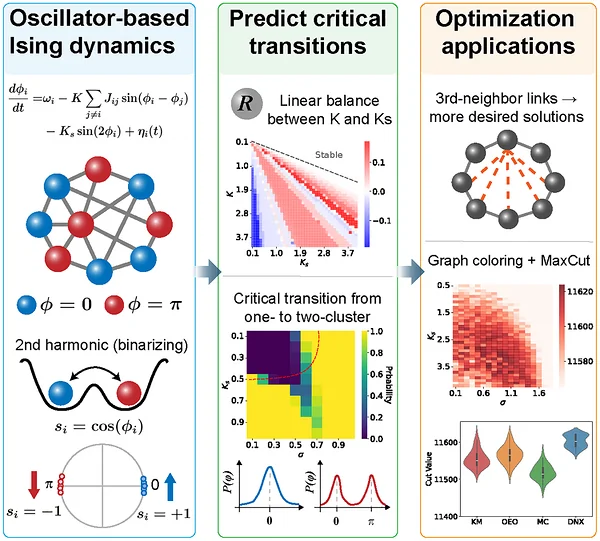

## 1 Introduction

Networks of coupled oscillators appear throughout the natural and engineered worlds, from pacemaker cells in the heart and firing neurons in the brain to power grids and flashing firefly swarms [1, 2, 3]. When individual parts interact with their neighbors, order can emerge spontaneously. Oscillators may synchronise completely, split into distinct clusters, or follow other collective patterns [4, 5, 6]. A widely studied model for such collective behavior is the Kuramoto model, in which oscillators interact through pairwise phase differences, and the dynamics is invariant under a uniform phase shift [7, 8].

Substantial analytical progress has been made in understanding synchronization and phase‐locked states in oscillator networks. For globally coupled populations, mean‐field approaches have provided analytical descriptions of synchronization transitions and collective dynamics [9]. For heterogeneous and network‐coupled Kuramoto‐type systems, analytical conditions have been developed to relate synchronization and stability to coupling strength, frequency heterogeneity, and network topology, with important applications to power grids [10, 11]. A broader overview for oscillator networks from an engineering and control perspective is provided in Ref. [12]. More recently, stable stationary states of power‐grid and Kuramoto oscillator networks have also been characterized through convex optimization [13].

A distinct application of coupled phase oscillators arises in oscillator‐based Ising machines, where continuous oscillator phases are used to encode binary Ising spins for combinatorial optimization [2, 14, 15]. In addition to Kuramoto‐type pairwise phase coupling, such systems incorporate a second harmonic term that favors two phase states separated by π [16]. Unlike the pairwise phase‐difference interaction of the standard Kuramoto model, the second harmonic term depends on the absolute phase and breaks its continuous rotational symmetry. This symmetry breaking enables the two preferred phase states to be mapped onto binary spin states in the Ising representation. The resulting dynamics therefore defines an oscillator‐based Ising system with Kuramoto‐type pairwise coupling and a second harmonic term.

More broadly, dynamical‐system approaches to Ising optimization have been extensively explored in classical, physical, semiclassical, and quantum‐inspired settings [17, 18]. Different realizations include quantum annealing [19], simulated annealing [20], coherent Ising machines [21, 22], oscillator‐based Ising machines [16, 23], simulated bifurcation [24], and other dynamical optimization schemes. Although these approaches rely on different physical mechanisms, they share the general idea of encoding a combinatorial optimization problem into an Ising‐type energy landscape and using controlled dynamics to search for low‐energy configurations. Related ideas also arise in quantum‐inspired optimization. For example, a mean‐field approximate optimization algorithm has been developed by replacing quantum evolution in the quantum approximate optimization algorithm with classical spin dynamics under a mean‐field approximation [25]. Such studies establish dynamical‐system optimization as a broad framework rather than a feature specific to oscillator‐based Ising machines.

For oscillator‐based Ising systems, however, a systematic analytical understanding of how interaction strength, the second harmonic term, stochastic fluctuations, and network structure jointly shape the dynamics remains limited [26, 27, 28]. In particular, it is important to determine the conditions for transitions between single‐cluster and two‐cluster states and to understand how structural modifications of the interaction network affect the accessibility of low‐energy configurations.

Noise can play a dual role in oscillator‐based Ising dynamics. Moderate fluctuations can facilitate transitions out of locally stable states and promote exploration of low‐energy configurations, whereas excessive noise may destabilize the binary phase states required for the Ising representation [29, 30, 31]. Long‐range, or non‐local, links can also reshape the accessible low‐energy configurations [32, 33]. Here, non‐local links refer to connections beyond nearest neighbors with respect to the prescribed ring‐based reference topology [34, 35]. This notion is defined by graph distance within the reference topology and does not imply a physical or geographical distance between optimization variables. The effects of non‐local links on the accessibility of low‐energy states remain to be characterized analytically.

In this work, we study an oscillator‐based Ising system with Kuramoto‐type pairwise coupling and a second harmonic term in both deterministic and stochastic settings. We develop an analytical framework to characterize the effects of coupling parameters, noise, and network structure. We identify critical transitions between single‐cluster and two‐cluster states and determine how noise shifts these boundaries. For the ring‐based topology, we further show that connections to third neighbors yield more desired solutions than the other added non‐local links. We examine these analytical predictions using graph‐coloring and MaxCut problems.

## 2 Results

### 2.1 Mapping the Ising Model to Oscillator‐Based Dynamics

The Ising model consists of a collection of coupled spins, $s_{i}$, which take two discrete values: $s_{i}\in \left\{+1,-1\right\}$. The energy of the model, known as the Ising Hamiltonian, is written as

(1) $$\begin{array}{c} H=-\sum_{i=1}^{N}h_{i}s_{i}-\sum_{1\leq i<j\leq N}J_{ij}s_{i}s_{j}, \end{array}$$

where $h_{i}$ represents the external magnetic field acting on the i‐th spin, and $J_{ij}$ represents the coupling interaction between the i‐th and j‐th spins. When interactions are positive (all $J_{i,j}>0$), the coupling is said to be *ferromagnetic*, and it is *antiferromagnetic* when interactions are negative. The field terms may equivalently be absorbed by introducing an auxiliary spin $s_{N+1}\equiv +1$ and setting $J_{i,N+1}=J_{N+1,i}=h_{i}$, such that the Hamiltonian can be written as $H=-\sum_{1\leq i<j\leq N+1}J_{ij}s_{i}s_{j}$.

The minimum‐energy state of an Ising Hamiltonian can encode the solution of a broad class of combinatorial optimization problems [36]. This correspondence has motivated a variety of dynamical approaches to Ising optimization in classical, physical, and quantum‐inspired settings [17, 25]. Such approaches use controlled dynamics to explore the corresponding energy landscape and seek low‐energy configurations. Here, we focus on oscillator‐based Ising dynamics with Kuramoto‐type pairwise coupling and a second harmonic term [16, 23, 28]. In the oscillator representation, the binary Ising spins are encoded by the oscillator phases according to $s_{i}=\cos\left(\phi_{i}\right)$, where $\phi_{i}=0$ and $\phi_{i}=\pi$ correspond to $s_{i}=+1$ and $s_{i}=-1$, respectively. For these binary phases, $\sin\phi_{i}=0$, and hence $s_{i}s_{j}=\cos\phi_{i}\cos\phi_{j}=\cos\left(\phi_{i}-\phi_{j}\right)$. Consequently, the pairwise Ising interaction $-J_{ij}s_{i}s_{j}$ is represented by the phase interaction $-J_{ij}\cos\left(\phi_{i}-\phi_{j}\right)$, while the field term $-h_{i}s_{i}$ is represented by $-h_{i}\cos\phi_{i}$. Taking the negative gradients of these phase‐energy terms with respect to $\phi_{i}$ yields the corresponding contributions $-J_{ij}\sin\left(\phi_{i}-\phi_{j}\right)$ and $-h_{i}\sin\phi_{i}$, respectively. The second harmonic term is included to promote the binary phase states required for the Ising representation. We consider the following oscillator‐based Ising dynamics

(2) $$\frac{d\phi_{i}}{dt}=\omega_{i}-Kh_{i}\sin\phi_{i}-K\sum_{j\neq i}J_{ij}\sin\left(\phi_{i}-\phi_{j}\right)-K_{s}\sin\left(2\phi_{i}\right).$$

Here, $\phi_{i}$ and $\omega_{i}$ are the phase and natural frequency of the i‐th oscillator, respectively, K is the global coupling strength, and $J_{ij}$ encodes the underlying interactions defined in the Ising Hamiltonian (1). The last term in Equation (2) is the *second harmonic term*, with strength $K_{s}$. Unlike the pairwise phase‐difference interaction of the standard Kuramoto model, this term depends on the absolute phase and breaks the global phase‐shift symmetry of the standard Kuramoto dynamics [16]. This symmetry breaking favors two phase states separated by π, providing the phase‐binarization mechanism. In the absence of the second harmonic term, $K_{s}=0$, the zero‐field dynamics is governed by the Kuramoto‐type pairwise interaction, and the stationary phases may exhibit a continuous phase distribution depending on the network structure and parameter regime (see Figure 1 Ai, ii). When $K_{s}\neq 0$, the second harmonic term favors two phase states near 0 and π, leading to a bimodal phase distribution (see Figure 1 Aiii, iv).

**FIGURE 1:**
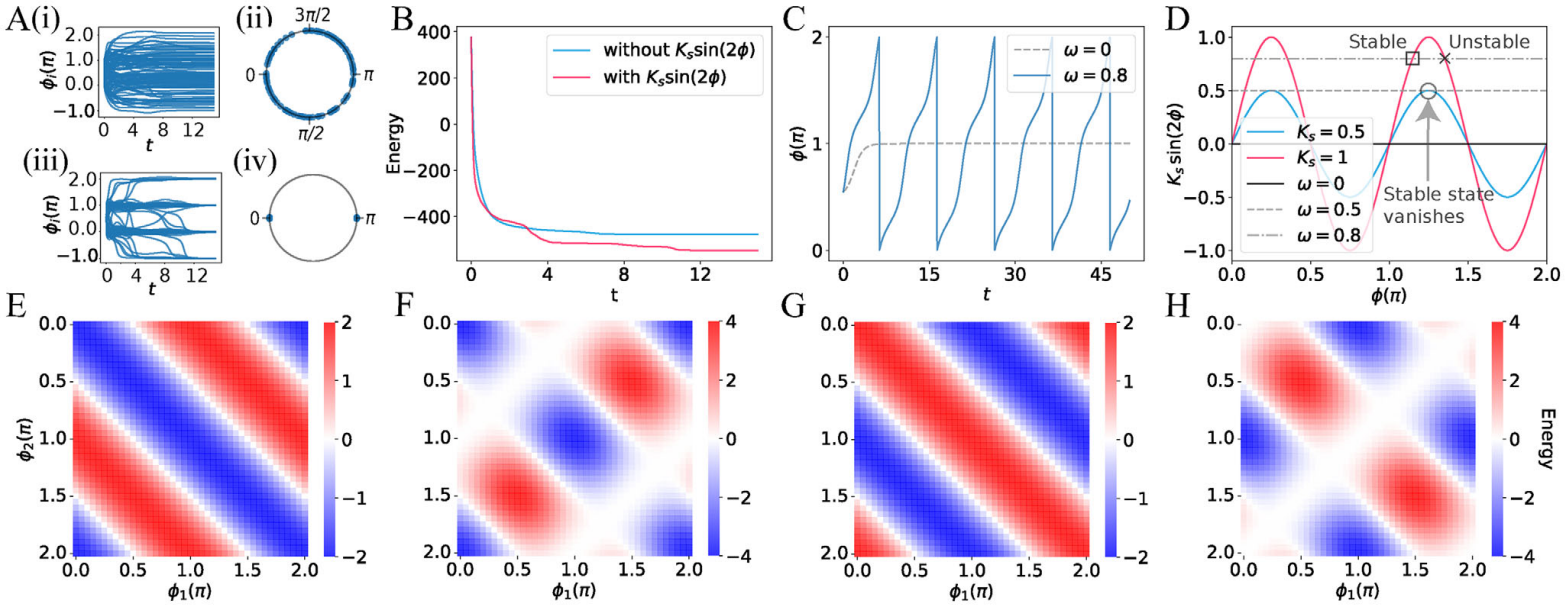
Influence of the second harmonic term, natural frequency, and magnetic interaction on the oscillator‐based Ising dynamics. (A) Effects of the second harmonic term $K_{s}\sin\left(2\phi_{i}\right)$. (i) and (ii) In the absence of the second harmonic term, the oscillators tend to synchronize within the interval [0,2π). The resulting final states exhibit a continuous distribution on a circle. (iii) and (iv) The second harmonic term disrupts the continuous synchronization, leading to the segregation of oscillators into two groups, one converging toward ϕ=0 and the other toward ϕ=π. (B) Energy decreases as the system evolves. (C) Different natural frequencies ω result in synchronized systems located at either moving (blue line) or stationary (gray dashed line) states. (D) The relationship between coupling strength ($K_{s}$) and natural frequency (ω) determines the type of synchronized states. Stationary states prevail when $|\omega /K_{s}|<1$, while moving states emerge when $|\omega /K_{s}|>1$. Stable stationary states vanish at $|\omega /K_{s}|=1$. (E,F) Energy distributions influenced by magnetic interaction type and second harmonic term. (E) The ferromagnetic interaction $J_{ij}>0$ without $K_{s}\sin\left(2\phi_{i}\right)$. (F) The ferromagnetic interaction ($J_{ij}>0$) with $K_{s}\sin\left(2\phi_{i}\right)$. Minimum energy values are located at binary phases with $\phi_{i}^{*}=0$ or π, and these phases tend to satisfy $\phi_{i}^{*}=\phi_{j}^{*}$ for adjacent nodes (i,j). (G) The antiferromagnetic interaction $J_{ij}<0$ without $K_{s}\sin\left(2\phi_{i}\right)$. (H) The antiferromagnetic interaction $J_{ij}<0$ with $K_{s}\sin\left(2\phi_{i}\right)$, the minimal values appear at the binary phases with $\phi_{i}^{*}=0$ or π, and these phases tend to have a phase difference π for adjacent nodes (i,j).

The energy function of the system (2) is given by

(3) $$\begin{array}{cc} E(\phi (t))= & -\sum_{i}\omega_{i}\phi_{i}\left(t\right)-K\sum_{i}h_{i}\cos\phi_{i}\left(t\right)\\ & -K\sum_{i<j}J_{ij}\cos\left(\phi_{i}\left(t\right)-\phi_{j}\left(t\right)\right)-\frac{1}{2}K_{s}\sum_{i}\cos\left(2\phi_{i}\right), \end{array}$$

where, with a ⊤ superscript denoting a matrix transpose, the quantity ϕ denotes the N component column vector $\phi ={\left[\phi_{1},\phi_{2},\text{…},\phi_{N}\right]}^{⊤}$. The time evolution of the system moves in the direction of the energy function, E(ϕ(t)), decreasing with time (dE/dt≤0) (see Figure 1B, where the red and blue lines indicate the behavior with and without the second harmonic term, respectively). Unless otherwise stated, the following analytical discussion of the basic oscillator dynamics and energy landscapes focuses on the zero‐field case $h_{i}=0$. Nonzero inhomogeneous fields are retained when required by specific optimization formulations, such as the graph‐coloring problem.

To gain insight into the system's behavior, we shall consider some simplified scenarios. In the absence of direct coupling of the oscillators (K=0), each oscillator has the tendency to rotate with its own natural frequency, $\omega_{i}$, as well as being influenced by the second harmonic term, and obeys ${\dot{\phi }}_{i}=\omega_{i}-K_{s}\sin\left(2\phi_{i}\right)$ [37, 38, 39, 40]. The value of the coupling strength $K_{s}$ determines different phase states of the system: when $|\omega /K_{s}|<1$, there is a stable stationary state; but when $K_{s}$ is reduced, so $|\omega /K_{s}|=1$, the system approaches a transition; and when $|\omega /K_{s}|>1$, non‐stationary ‘moving’ states may emerge (see Figure 1C,D). In what follows, we set the natural frequencies to zero ($\omega_{i}=0$) to focus on the oscillator‐based Ising setting, where the stationary phase configurations encode the Ising spins. This assumption allows us to analyze the effects of the coupling parameters and network structure on the system's behavior. Accordingly, the present analysis is restricted to the zero‐natural‐frequency case and does not cover general oscillator networks with heterogeneous intrinsic frequencies.

In the absence of the second harmonic term ($K_{s}=0$), the energy landscape in Equation (3) is mainly determined by the pairwise interactions, i.e., the $J_{ij}$. If the interactions are ferromagnetic, adjacent oscillators tend to approach phase synchronization, and minimal values of the energy are located at $\phi_{i}=\phi_{j}$ for all nodes (see Figure 1E). If the interactions are antiferromagnetic then the energy landscape has a different form (see Figure 1G) with minimal energy values lying at the bottom of valleys (see Figure 1E,G). The presence of the second harmonic term ($K_{s}\neq 0$) modifies the energy landscape of the oscillator system [16]. Discrete basins of attraction emerge with minimal energy values located at oscillator phases of either 0 or π (see Figure 1F,H). For $J_{ij}>0$, all oscillators approach phase synchronization with minimal energy values at $\phi_{i}^{*}=\phi_{j}^{*}$ (see Figure 1F). For $J_{ij}<0$, the energy is minimized when adjacent oscillators satisfy $\phi_{i}^{*}+\phi_{j}^{*}=\pi$, and this suggests that in the corresponding Ising model, the adjacent spins tend to have the opposite orientations (see Figure 1H; see also Figure S1).

In the binary‐phase regime, $\cos\phi_{i}^{*}=s_{i}$, $\cos\left(\phi_{i}^{*}-\phi_{j}^{*}\right)=s_{i}s_{j}$, and $\cos(2\phi_{i}^{*})=1$. With $\omega_{i}=0$, the oscillator energy at a stationary binary‐phase state reduces to

(4) $$E\left(\phi^{*}\right)=-K\sum_{i}h_{i}s_{i}-K\sum_{i<j}J_{ij}s_{i}s_{j}-NK_{s}/2=KH-NK_{s}/2.$$

The term $-NK_{s}/2$ is an additive constant and does not affect the relative energies of different binary spin configurations. Apart from the overall factor K and this additive constant, the oscillator energy reproduces the Ising Hamiltonian, including both the inhomogeneous field terms and the pairwise interactions. This correspondence is established for stationary states in the binary‐phase regime and does not by itself imply that the oscillator dynamics will reach the global minimum in large‐scale Ising problems. The ability of the dynamics to access low‐energy configurations in larger systems is examined separately below using graph‐coloring and MaxCut problems.

### 2.2 Spectral Reduction and Relaxation to the Stationary State

Motivated by the correspondence between the oscillator energy and the Ising Hamiltonian in the binary‐phase regime, we next analyze the high‐dimensional oscillator dynamics using spectral dimension reduction. Throughout this subsection, we retain the assumption $\omega_{i}=0$, so that the reduced dynamics characterizes the stability of stationary states in the oscillator‐based Ising system with zero natural frequencies. Dynamical‐system formulations provide a useful framework for exploring Ising optimization problems [17, 26]. However, the computational cost of numerical integration and the number of possible Ising configurations increase rapidly with the number of coupled oscillators N, making large systems increasingly difficult to analyze. To alleviate these limitations and gain theoretical insight into how network structure impacts the energy landscape, we adopt an analytical approach based on spectral dimension reduction of the dynamics [41, 42].

The spectral dimension reduction builds on [41, 42] and is described in detail in the *Methods* section. Intuitively, the dynamics is simplified by searching for a linear, global observable R representing the weighted instantaneous activity of the global state of the system (2)

(5) $$\begin{array}{c} R=a^{⊤}\phi . \end{array}$$

Here $a={\left[a_{1},a_{2},\text{…},a_{N}\right]}^{⊤}$ denotes normalized weights of each oscillator, with $\sum_{i}a_{i}=1$. The key is to determine weights that properly take into account the importance of each node for the dynamics. In the literature, both analytical and simulated results have shown that the observable R is a good indicator of the oscillatory system's behavior [42], and that it has the potential of providing intuitive insight into the interplay of structure and dynamics. Under the assumption that the system is close to a stable stationary state with observable $R^{*}$, we derive an equation for the time evolution of the deviation $\delta R=R-R^{*}$

(6) $$\begin{array}{c} \dot{\delta }R\approx -\left[K\alpha \left(\beta -1\right)\cos\left(\left(\beta -1\right)R^{*}\right)+2K_{s}\right]\delta R, \end{array}$$

where α is determined by the eigenvalue of the network structure with the largest magnitude, reflecting the most significant mode of the network's dynamics. The term β≥1 is related to the eigenvector corresponding to this largest eigenvalue and the degree matrix, indicating how the network topology influences the system behavior. Equation (6) determines how the high dimensional system of networked oscillators returns to a stationary state after small perturbations (see Figure 2A,E), and clearly shows the effects of the global coupling, K, and the second harmonic coupling, $K_{s}$ (see Figure 2,F). Further derivations and perturbation tests are provided in Section S3.1 and Figures S2 and S3.

**FIGURE 2:**
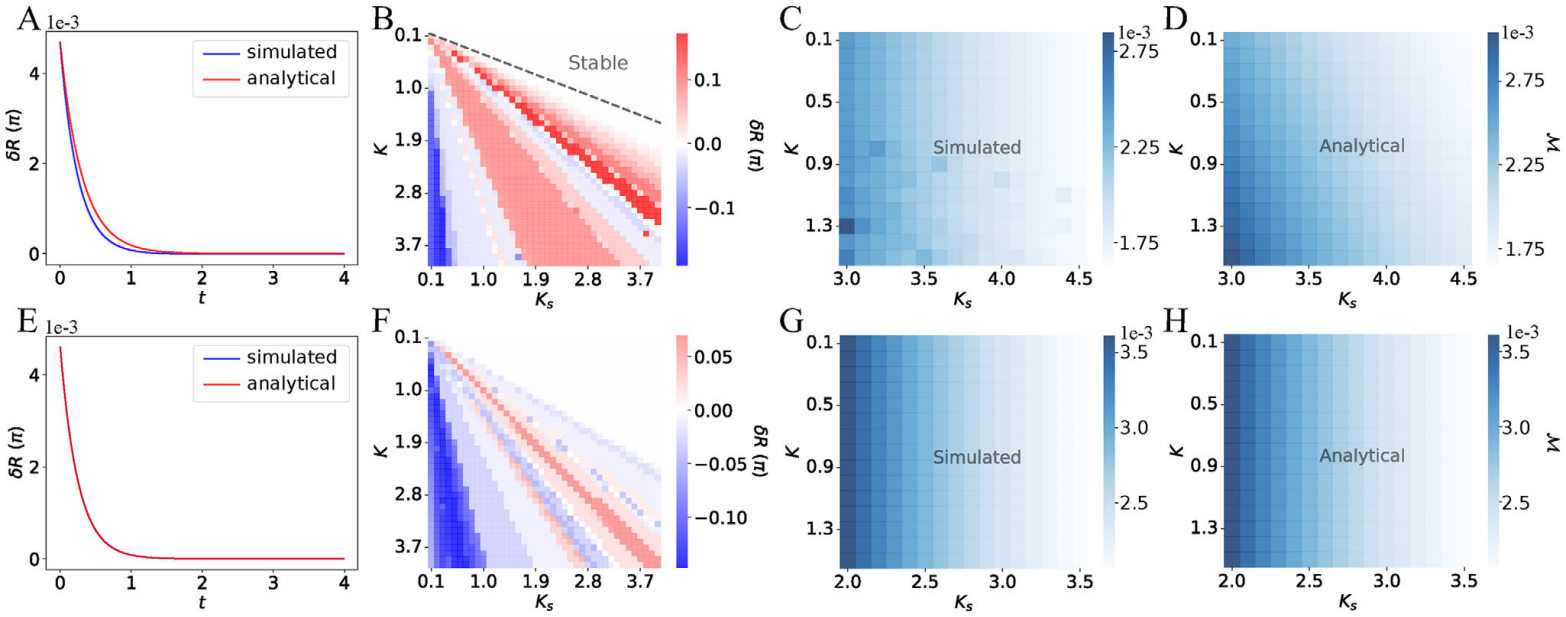
Spectral dimension reduction analysis of coupling parameters K and $K_{s}$ with small perturbations in networks. (A–D) Results derived from an Erdös–Rènyi network with N=100 and the connection probability of 0.1. (A) The global observable δR, obtained around stationary states from simulations and analytical expressions, converges to 0 after small perturbations, indicating the system returns to its original stationary state. (B) Exploring the boundary of stability for a fixed stationary state by varying the parameters K and $K_{s}$. The white triangular region in the upper right signifies the system's return to the original state. The gray dashed line, determined by Equation (6), represents the analytical boundary indicating the linear balance relations between K and $K_{s}$ for stability. Synchronization is achieved by calculating δR after a long simulation time (t=100 s). (C) and (D) represent simulated and analytical coherency performance metric M, respectively. The assumption for analytical M in Equation (7) is that the system can converge to the original state, so the parameters K and $K_{s}$ are primarily considered from the white triangular regions in (B). (E–H) Derived from a regular network with N=100 where each node connects to its nearest five neighbors, resulting in a node degree of 10. (E) Exploration of δR variations over time t. (F) The stability boundary of δR with varying the parameters K and $K_{s}$. (G) and (H) demonstrate that the coherency performance metric M is solely influenced by the second harmonic term in a network where each node has the same degree.

In Equation (6), as $K_{s}$ increases, the coefficient of δR decreases, resulting in a faster rate at which δR tends to zero. This indicates that the system returns to its stationary state more quickly and easily. Most of the simulations in Ref. [42] show that β is close to 1. For a small $R^{*}$, $\cos(\left(\beta -1\right)R^{*})\geq 0$, and the term α<0, these imply that as K decreases, the rate at which δR tends to zero increases, meaning the system returns to its stationary state more quickly and easily. The coefficients on the right side of Equation (6) determine the linear balance between K and $K_{s}$, which indicates the stability boundary of the system. Specifically, it means that the parameters K and $K_{s}$ determine the conditions under which the system can return to its original stationary state after small perturbations. In Figure 2B,F, we use two representative network structures to validate the linear balance between K and $K_{s}$. The results show that as $K_{s}$ increases, the region where δR=0 gradually expands, indicating that the system returns to its stationary state. However, as K increases, the region where δR=0 gradually shrinks. These figures demonstrate that the stability and synchronization properties of the system are governed by the interplay between K and $K_{s}$, which in turn is influenced by the underlying network topology through α and β.

To quantify further the transient response to perturbations, we define a coherency performance metric, M, as $M=\int_{0}^{\infty }\left|\delta R(t)\right|dt$ [43]. The metric M measures the energy dissipated by the oscillators during the return of the system to the stationary state. The quantity δR converges over time, and the metric M is found to be given by

(7) $$\begin{array}{c} M=\frac{\left|\delta R(0)\right|}{K\alpha \left(\beta -1\right)\cos\left(\left(\beta -1\right)R^{*}\right)+2K_{s}}. \end{array}$$

The derivation of Equation (7) is provided in Section S3.1. When $J_{ij}<0$ and β>1, we have α<0 and the expression (7) implies that the system, with strong coupling K and weak second‐harmonic coupling $K_{s}$, dissipates a high amount of energy as it returns to the stationary state (see Figure 2,D). In contrast, if each node has the same degree and β=1, the coherency performance metric is determined solely by $K_{s}$ (see Figure 2G,H).

In conclusion, we use the global observable to describe the stability of stationary states via spectral dimension reduction. We derive the linear balance between K and $K_{s}$, which provides a systematic approach for tuning these parameters to achieve improved performance and faster convergence following small perturbations. Our findings indicate that, for networks with β>1, increased global coupling requires higher energy to restore the original state. However, for networks where nodes have similar degrees, energy consumption is primarily influenced by $K_{s}$.

### 2.3 Fokker–Planck Equation Reveals Phase Transition

The integration of the deterministic dynamical system (2) allows the identification of local minima of the energy landscape in the vicinity of the initial state. To explore a broader portion of the energy landscape and improve access to lower‐energy configurations, mechanisms that facilitate escape from local minima are often introduced. Here, we examine the role of additive noise in this process. To analyze effects of the noise and coupling parameters, we adopt a Fokker–Planck equation to investigate the mean‐field case. In the limit of an infinite number of oscillators, the macroscopic behavior of the stochastic system is described by [44]

(8) $$\begin{array}{cc} \frac{\partial P}{\partial t}= & -\frac{\partial }{\partial \phi }\left\{\left[-K\int_{-\pi }^{\pi }d\phi^{\prime }\sin\left(\phi -\phi^{\prime }\right)n\left(\phi^{\prime },t\right)-K_{s}\sin\left(2\phi \right)\right]P\right\}\\ & +\sigma^{2}\frac{\partial^{2}P}{\partial \phi^{2}}, \end{array}$$

where P(ϕ,t) is the probability distribution of $\phi_{i}$ at time t, and σ represents magnitude of the noise. The term $n\left(\phi ,t\right)=\frac{1}{N}\sum_{j=1}^{N}\delta \left(\phi_{j}\left(t\right)-\phi \right)$ denotes the normalized number density of the oscillators with phase ϕ at time t.

The procedure for solving the Fokker–Planck equation is detailed in the *Methods* section. By assuming that the system reaches a stationary state, the time derivative of the probability distribution P(ϕ,t) becomes zero, reducing the equation to an ordinary differential form for n(ϕ). Two self‐consistent equations for the averages of cos(ϕ) and sin(ϕ) are derived, providing the synchronization parameter Ψ. The probability distribution n(ϕ) is expressed as an exponential function of Ψ, with the periodic boundary condition ensuring its periodicity. Solving these equations yields n(ϕ), which reveals the system's synchronization dynamics. The derivative of n(ϕ) yields the transition line between one and two clusters stationary distributions,

(9) $$\begin{array}{c} K_{s}^{c}=\frac{K\Psi }{2}. \end{array}$$

The transition line described by Equation (9) indicates the boundary between different synchronized states. When $K_{s}<K_{s}^{c}$, the oscillators tend to synchronize in one cluster, while for $K_{s}>K_{s}^{c}$, they split into two synchronized clusters. Figure 3A shows the transition line analytically predicted by Equation (9). In the upper left of the transition line, the system has a one‐cluster state. With the increase of the noise intensity and the coupling $K_{s}$, the system undergoes a phase transition and two‐cluster states appear in the lower right of the transition line. The analytical transition line illustrates the system's sensitivity to noise and the second harmonic, which is crucial for understanding the behavior of large‐scale oscillator networks. Further derivations and analytical results are provided in Section S4 and Figures S4 and S5.

**FIGURE 3:**
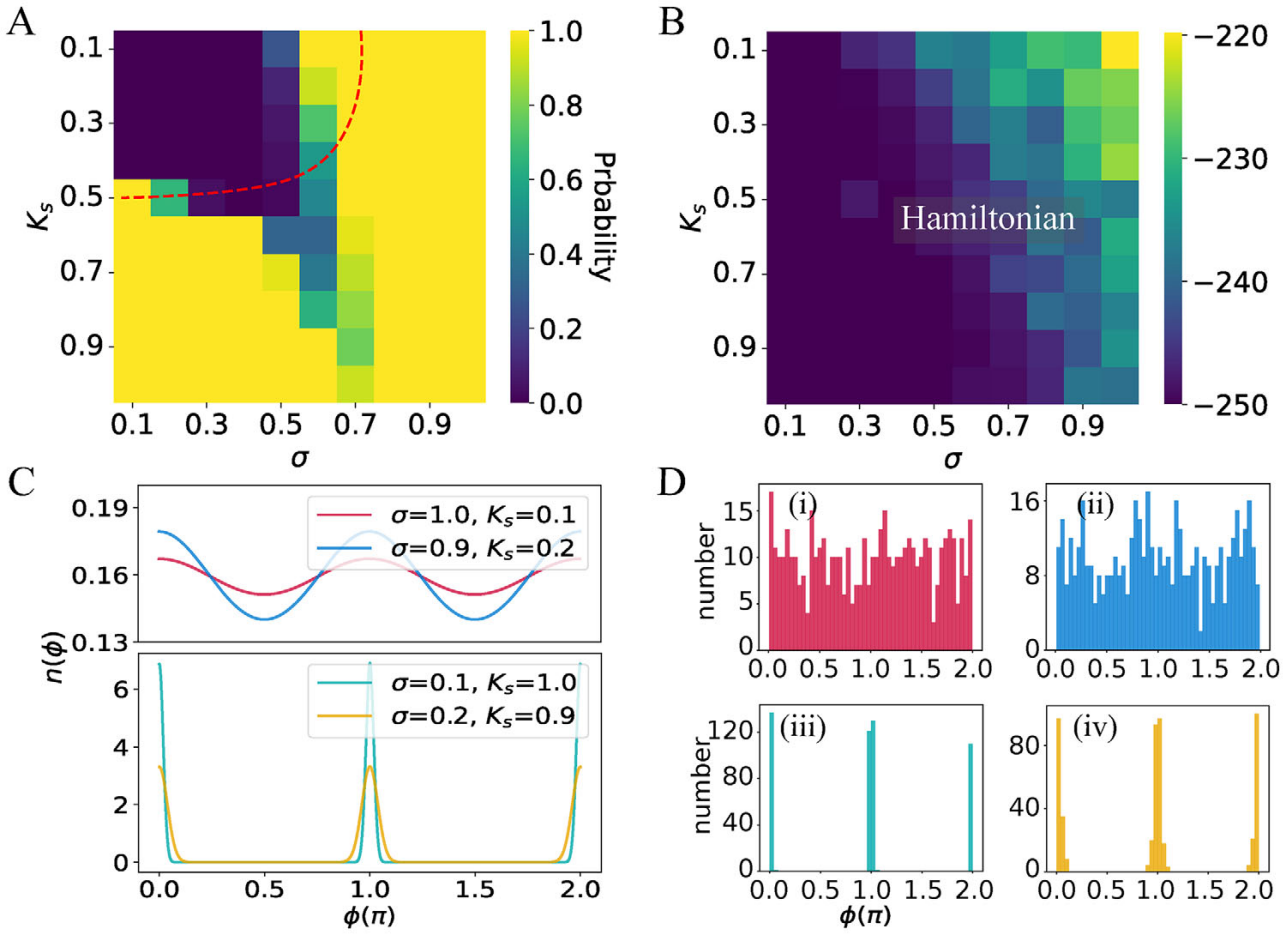
Fokker–Planck equation reveals a noise‐induced phase transition. (A) Phase transition line determined by σ and $K_{s}$. We simulate the system 50 times and display the probabilities of the two stationary clusters. (B) The minimal Ising Hamiltonian distribution among 50 simulations. (C) Lines of the extended n(ϕ). (D) The simulated phase distributions with four parameters taken from (C). The color indicates the parameter setting. (B,D) are the extended results of the Fokker–Planck equation based on the antiferromagnetic and uniform interaction. All of the simulation results are obtained with problem size N=500.

The above analysis of the Fokker–Planck equation is obtained with $J_{ij}=1$ between coupled nodes i and j, and in this case this term drives the system to approach synchronization with $\phi_{i}=\phi$ for all oscillators. While as shown in Figure 3C, the system could also exhibit two‐cluster states. This result is similar to the case of the antiferromagnetic couplings with $J_{ij}<0$ in the Ising Hamiltonian [16, 26]. Therefore, we hypothesize that the above analysis of the density function n(ϕ) could potentially provide insights for this case of the effects of the parameter space on the system behaviors.

Numerically, we integrate the stochastic system with the antiferromagnetic coupling $J_{ij}=-1$ for coupled oscillators and obtain the projection of the Ising Hamiltonian on the parameter space of $K_{s}$ and σ (see Figure 3B). Low energy values are mainly distributed in the regions of $K_{s}>\sigma$ (lower left corner in Figure 3B), while high values emerge in the regions of $K_{s}<\sigma$ (upper right corner in Figure 3B). To explain further the mechanisms, we select four representative settings of $K_{s}$ and σ, and plot the corresponding analytical density functions n(ϕ) based on the analytical prediction (19) (in Figure 3C) and numerical solutions (in Figure 3D). For $K_{s}<\sigma$, n(ϕ) has a wide distribution near ϕ=0 or π (see upper panel of Figure 3C). In this case, n(ϕ)>0 near ϕ=π/2 or 3π/2 implies that many oscillators with $s_{i}\approx 0$ are randomly distributed and do not contribute to the minimization of the Ising Hamiltonian.

For $K_{s}>\sigma$, the analytical distributions of n(ϕ) show that most oscillators are located near ϕ=0 or π (see lower panel of Figure 3C). The numerical solutions validate this result (see Figure 3D(iii),(iv)). This explains why the Ising Hamiltonian in this parameter region could be minimized. These results indicate that moderate noise may facilitate exploration of low‐energy Ising configurations, whereas excessive noise degrades phase binarization and optimization performance.

By using the Fokker–Planck equation, we derive an analytical expression deciding the state distribution. The results show that the stationary phase distribution of the oscillator‐based Ising system is either a one‐ or two‐cluster distribution. The transition line of the state distribution is decided by Equation (9). The system tends to locate in two stationary clusters when $K_{s}$ and σ cross the transition line. For the antiferromagnetic and uniform coupling, we extend the results of the Fokker–Planck equation and explain the influence of the noise and second harmonic term on the minimal values of the Ising Hamiltonian, which are related to the optimal solutions of the combinatorial optimization problems. We find that large noise could impair the connections between the oscillator energy and Ising Hamiltonian. The minimum values of the Ising Hamiltonian are mainly distributed in the regions with $K_{s}>\sigma$.

### 2.4 Partition Function and Graph Coloring Problem

The above analysis reveals the effects of coupling strengths and noise magnitude on the system behavior within the mean‐field framework. Additionally, the underlying network structure also plays a central role in determining the likelihood of the system occupying each state. Here, we focus instead on non‐trivial network structures and proceed as follows. In the stochastic setting, one needs to find the relation between the partition function, at a finite temperature T of the Ising model and the trajectories of the associated noisy oscillator‐based Ising dynamics. The partition function contains information about the probability of each energy configuration, and captures how the network structure impacts the stochastic properties of the model. Note that the lowest energy state of a given Hamiltonian is in general unknown for combinatorial optimization problems, but that its value can be estimated in specific cases.

To go further, we take the graph coloring problem, which consists in assigning m colors to N nodes, as a concrete problem that can be formulated as the minimization of an Ising model. The Hamiltonian representation of the graph coloring problem is derived in the *Methods* section. Here, we consider a ring as a reference topology, in which each node has a well‐defined nearest‐neighbor relation. We then introduce an additional edge between nodes that are not nearest neighbors on the ring. Throughout this analysis, we refer to such an added edge as a non‐local link. Thus, the term non‐local is defined relative to the reference topology rather than to a physical distance between optimization variables. The Ising Hamiltonian and the corresponding partition function are provided in the *Methods* section. Intuitively, the solution of the partition function in the limit of zero temperature yields the number of solutions where the corresponding Ising energy is zero. For a simple ring, we can analytically obtain the number of desired solutions,

(10) $$Z_{2}={\left(c_{1}^{N}+c_{2}^{N}\right)}^{m},$$

where $c_{1}≜\left(1+\sqrt{5}\right)/2$ and $c_{2}≜\left(1-\sqrt{5}\right)/2$. The constants $c_{1}$ and $c_{2}$ are the two eigenvalues derived from the connection mode matrix of the adjacent nodes, i.e., the spectral properties of the corresponding graph. The derivation of Equation (10) and related results are provided in Section 5.2.1 and Figure S6. Equation (10) shows that $Z_{2}$ grows exponentially with the number of oscillators N and the number of colors m (see Figure 4A).

**FIGURE 4:**
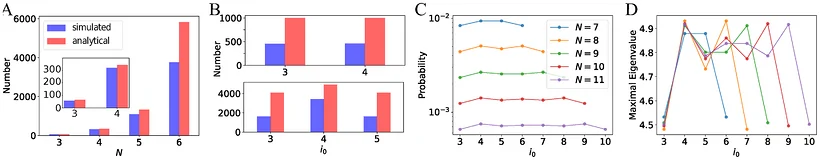
The partition function describes the number of desired solutions in the ring‐based topology. (A) Exponential growth of desired solutions with increasing size N. (B) Variations in the number of desired solutions for a fixed N with different added non‐local links. Upper and lower panels correspond to N=5 and N=6, respectively. (C) Influence of different added non‐local links on the probabilities of desired solutions. The largest probability among the considered connections is obtained for a link to the third neighbor. (D) The largest eigenvalue of the graph Laplacian as a function of the position of the added non‐local link, exhibiting trends similar to those in (C). The line colors indicate different problem sizes N, as specified in (C).

After adding one non‐local link between node 1 and node $i_{0}$, the corresponding $Z_{2}$ is approximated as

(11) $$\begin{array}{c} Z_{2}\approx {\left(\frac{c_{1}^{N}+c_{2}^{N}/2-c_{1}^{i_{0}-1}c_{2}^{N-i_{0}+1}-c_{1}^{N-i_{0}+1}c_{2}^{i_{0}-1}}{5}\right)}^{m}. \end{array}$$

For the ring‐based topology, the effect of non‐local links on the number of optimal solutions is described in Equation (11). Since $Z_{2}$ represents the number of desired solutions, with the addition of non‐local links, the number of desired solutions Equation (11) decreases in comparison with Equation (10) (see Figure 4B, compared to Figure 4A without non‐local links). When $i_{0}=4$ or N−2, representing a connection to the third neighbor (either left or right), $Z_{2}$ reaches its maximum value in Equation (11). This indicates that linking to a third neighbor induces the maximal number of desired solutions, compared to linking to other neighbors (see Figure 4C). Further details are provided in Section S5.2.2 and Figures S7 and S8.

We use the spectral properties of the corresponding graph to explain the result. We denote by L the Laplacian matrix of the graph, where $L_{ii}=\sum_{j}A_{ij}$ and $L_{ij}=-A_{ij}$ for i≠j. For a fixed problem size N, we compute the maximum eigenvalue of the Laplacian matrix for different values of $i_{0}$ (from 3 to N−1). The results show that when the non‐local link connects to the third neighbor, the maximum eigenvalue reaches its highest value across various problem sizes (see Figure 4D). The line trends in Figure 4D are very similar to Figure 4C, indicating that the maximum eigenvalue can be used to describe the effects of non‐local link on the number of optimal solutions. A larger maximum eigenvalue leads to improved stability and synchronization of the system, allowing energy to spread more effectively through the network. This results in a more favorable distribution of energy states and increases the number of desired solutions. For the ring‐based topology, connecting to the third neighbor is associated with more favorable spectral properties and a larger number of desired solutions than the other added connections.

Overall, the partition‐function analysis shows that the number of desired solutions grows with problem size, while the introduction of non‐local links generally reduces this number. Among the considered non‐local connections, linking to the third neighbor yields the largest number of desired solutions. Additional partition‐function results are presented in Section S5.3 and Figures S9–S11.

To demonstrate the practical advantage of our analytical description of the Kuramoto‐type oscillator model, we consider the case when the global minimum of the energy landscape is known, namely the case of planar maps. The four‐color theorem guarantees that any planar map can be colored using at most four colors [45, 46]. Here, we take the American mainland map with N=47 regions as an example and simulate the Kuramoto‐type oscillator model with m=4 colors.

The final states provide a valid coloring of the map (see Figure 5A). Additional graph‐coloring results are provided in Figures S12 and S13. Following the spectral dimension reduction analysis, we investigate the effects of K and $K_{s}$ on the global observable δR. The results show the analytically predicted linear balance between K and $K_{s}$, which can be used to speed up convergence following small perturbations away from the stationary state (see Figure 5B). Additionally, we calculate the state density function via the Fokker–Planck equation and find that the minimal values of the Ising Hamiltonian are mainly distributed in the region with $K_{s}>\sigma$ (see Figure 5C).

**FIGURE 5:**
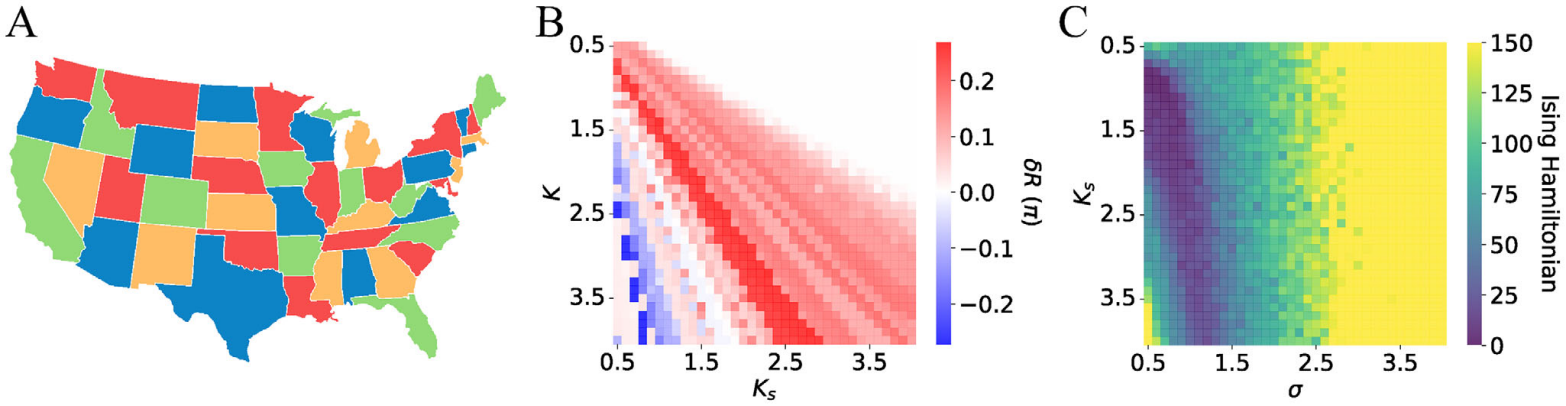
Application of the Kuramoto‐type oscillator model to the graph‐coloring problem. (A) Efficient coloring of the American mainland map using four colors with the Kuramoto‐type oscillator model. For the simulation, the selected parameters are K=2.0, $K_{s}=1.5$, and σ=0.2. (B) Deviation of global observable δR vs. coupling parameters ($K_{s},K$). The boundary of the white triangle block in the upper right panel illustrates the linear balance between K and $K_{s}$. (C) Minimal values of the Ising Hamiltonian mainly concentrate in the area with $K_{s}>\sigma$.

### 2.5 Solving MaxCut Combinatorial Optimization Problems

The MaxCut problem aims to partition the nodes into two sets such that the number of edges between the two sets (i.e., the cut) is maximized [16, 47, 48]. Given a network G=(V,E) with nodes V and edges E, the aim is to group nodes into two subsets $G_{1}=\left(V_{1},E_{1}\right)$, $G_{2}=\left(V_{2},E_{2}\right)$, with $V_{1}\cap V_{2}=⌀$, so that the number $E_{\mathrm{cut}}$ of edges between the two subsets is maximized. The graph partition is mapped to Ising spins by assigning +1 to a spin i if $i\in V_{1}$, and −1 if $i\in V_{2}$. The Ising Hamiltonian in Equation (1) can then be rewritten as $H=-\sum_{i<j}J_{ij}-2E_{\mathrm{cut}}$, where the cut size is defined as $E_{\mathrm{cut}}=\frac{1}{2}\sum_{i<j}J_{ij}\left(s_{i}s_{j}-1\right)$. The maximum cut is achieved when the Ising Hamiltonian is minimized.

Based on the oscillator energy–Ising Hamiltonian correspondence, we construct the corresponding oscillator dynamics and consider the G1 graph, a benchmark instance from the G‐set, as an example [16, 49]. First, we investigate the global dynamics of the system via the spectral dimension method. As shown in Figure 6A, the node degrees in G1 exhibit a concentrated distribution, which suggests that the term β in Equation (16) is close to 1. Considering small perturbations from stationary states, we focus on the stability of the global observable R in Equation (5), and numerically and analytically determine the convergence of δR to the stationary state in Figure 6B. The analytical prediction is based on Equation (6). Additionally, we calculate the projection of δR onto the parameter space of K and $K_{s}$ in Figure 6C, and results also show the existence of the linear balance in Figure 2B,F. In the presence of noise, we simulate the corresponding stochastic dynamics 50 times for each parameter pair $\left(K_{s},\sigma \right)$ over the ranges $K_{s}\in \left[0.5,4.2\right]$ and σ∈[0.1,1.8]. For each parameter pair, the maximum cut value obtained among the 50 simulations is shown in Figure 6D. The results show that high cut values are predominantly distributed in the region $K_{s}>\sigma$, consistent with the preceding Fokker–Planck analysis. In particular, the best‐known cut value of 11624 [16] is reached at $\left(K_{s},\sigma \right)=\left(3.4,0.9\right)$, (3.5,0.7), and (3.7,1.1), all of which satisfy $K_{s}>\sigma$. These results indicate that stronger second‐harmonic effects relative to noise favor binary phase localization and facilitate access to high‐quality MaxCut solutions. At large noise magnitudes, particularly σ≥1.5, the cut values generally decrease, indicating that excessive noise degrades the optimization performance.

**FIGURE 6:**
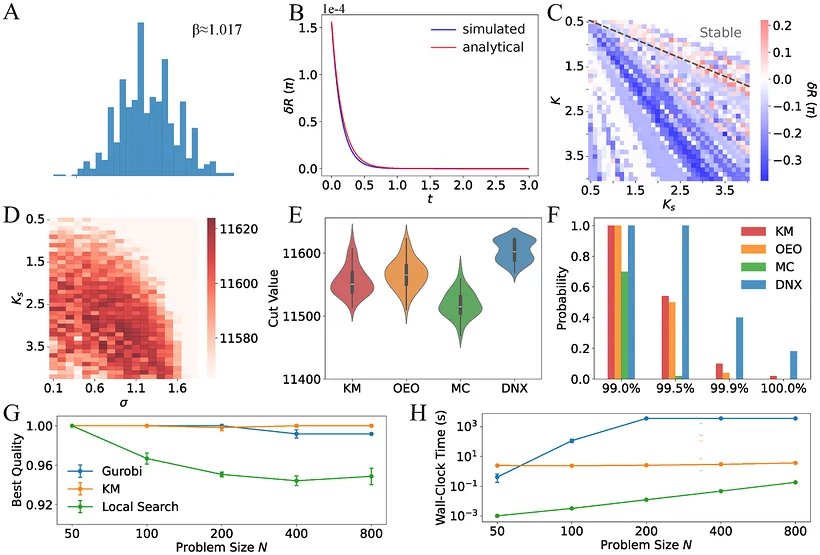
Solution of the MaxCut problem using the Kuramoto‐type oscillator model. Panels (A–F) use the G1 instance with 800 nodes and 19176 edges. (A) Network degrees exhibit a central distribution. (B) The simulated and analytical δR converge to 0. (C) Linear balance between K and $K_{s}$ to maintain stable stationary states. The dashed line represents the analytical boundary determined by Equation (6). (D) Maximum cut values from 50 simulations for each $\left(\sigma ,K_{s}\right)$ pair. (E) Distributions of the best cut values obtained from 50 independent runs of the four methods under their respective fixed parameter settings. (F) Probabilities of reaching 99%, 99.5%, 99.9%, and 100% of the baseline value 11624. (G) Best normalized solution quality vs. problem size N. Results are averaged over three independent graph instances, with error bars indicating the standard deviation across graphs. (H) Wall‐clock computation time vs. problem size N.

Additionally, we compare the MaxCut performance of the Kuramoto‐type oscillator model with three alternative methods: an OEO‐based coherent Ising machine (CIM) [21], Monte Carlo simulated annealing, and D‐Wave NetworkX with a classical simulated annealing sampler. The Monte Carlo implementation is based on a publicly available code repository (https://github.com/gautham‐umasankar), while D‐Wave NetworkX provides graph‐optimization interfaces for solving problems such as MaxCut (https://dnx.readthedocs.io). Additional parameter settings and results are provided in Section S7 and Figures S14–S17. The OEO‐based CIM evolves a set of nonlinear oscillator states with self‐feedback and mutual coupling to search for low‐energy Ising configurations. The Monte Carlo method employs single‐spin Metropolis updates together with an annealing schedule, whereas D‐Wave NetworkX is combined here with the classical simulated annealing sampler provided by the D‐Wave Ocean tools. In contrast, the Kuramoto‐type oscillator model performs the search through the continuous‐time dynamics of coupled phase oscillators with a second‐harmonic term that promotes binary phase states.

Each method is evaluated over 50 independent runs under its corresponding parameter settings. The maximum cut values obtained by the Kuramoto‐type oscillator model, OEO‐based CIM, Monte Carlo method, and D‐Wave NetworkX are 11624, 11623, 11576, and 11624, respectively. Thus, both the Kuramoto‐type oscillator model and D‐Wave NetworkX reach the best‐known value, while the OEO‐based CIM obtains a value only one edge below it and the Monte Carlo method gives a lower maximum value. For the comparisons in Figure 6E,F, we use K=1.5, $K_{s}=3.4$, and σ=0.9 for the Kuramoto‐type oscillator model. For the OEO‐based CIM, the self‐feedback coefficient and coupling strength are fixed at α=0.9 and β=0.05, respectively, and the standard deviation of the Gaussian noise is set to 0.1. Figure 6E shows the distributions of the best cut values obtained from the 50 runs of each method. Figure 6F further compares the probabilities of reaching 99%, 99.5%, 99.9%, and 100% of the baseline value 11624.

To assess scalability beyond the single G1 instance, we further benchmark the Kuramoto‐type oscillator model against Gurobi and 1‐flip local search on random 10‐regular graphs with N=50, 100, 200, 400, and 800. Three independent graph instances are generated for each problem size. The Kuramoto‐type oscillator model and local search are each evaluated over 20 independent runs per graph. Gurobi is run once per graph using one CPU thread, a target MIP gap of zero, and a time limit of 3600 s. For the Kuramoto‐type oscillator model, the parameters are fixed at K=2.0, $K_{s}=3.0$, and σ=0.8 for all graph sizes.

For each graph, the solution quality of each stochastic method is evaluated using the best cut obtained over 20 runs. The cut value is normalized by the Gurobi‐certified optimum when available and otherwise by the best observed feasible cut among the three methods. As shown in Figure 6G, the Kuramoto‐type oscillator model maintains a best normalized quality close to unity over the investigated range of problem sizes and achieves higher solution quality than 1‐flip local search for N≥100. Gurobi certifies the optimum for all instances with N=50 and 100. For N≥200, however, Gurobi reaches the 3600‐s time limit without certifying optimality. Consequently, its reported values for these sizes are incumbent solutions, and normalization is performed relative to the best observed feasible cut rather than a certified optimum. In particular, the Kuramoto‐type oscillator model obtains better feasible cuts than the corresponding Gurobi incumbents for the N=400 and 800 instances, although global optimality remains unverified. Detailed cut values, Gurobi solver status, and MIP gaps are reported in Table 1.

**TABLE 1:** MaxCut solution quality on random 10‐regular graphs.

Method	N=50	N=100	N=200	N=400	N=800
KM	176/178/176	362/360/362	728/726/730	1458/1460/1458	2908/2920/2904
Local Search	176/178/176	348/348/352	692/692/696	1376/1372/1384	2774/2742/2768
Gurobi	176/178/176	362/360/362	728/726/734	1440/1448/1452	2884/2896/2880
Gurobi Status	O/O/O	O/O/O	TL/TL/TL	TL/TL/TL	TL/TL/TL
MIP Gap (%)	0/0/0	0/0/0	4.53/4.41/3.68	8.61/8.15/7.64	12.27/12.19/12.33

* Values separated by slashes correspond to the three independent graph instances. O and TL denote optimal and time limit, respectively. MIP gap denotes the relative gap between the incumbent solution and the best bound reported by Gurobi.

Figure 6H shows the corresponding computation times. For the Kuramoto‐type oscillator model and local search, the runtime is first averaged over 20 independent runs for each graph. For all three methods, the reported values are the mean ± standard deviation across the three graph instances, with Gurobi run once per graph. All timing experiments were performed on the same workstation equipped with a 13th Gen Intel Core i7‐13650HX processor and 16 GB of RAM. All methods were executed using a single CPU thread, and no GPU acceleration was used. Local search is the fastest method but yields lower‐quality solutions as the problem size increases. The mean runtime of the Kuramoto‐type oscillator model increases only moderately, from approximately 2.5 s at N=50 to 3.7 s at N=800, while maintaining high solution quality. By contrast, the Gurobi runtime increases rapidly and reaches the prescribed 3600 s limit for every instance with N≥200. The corresponding computation times are reported in Table 2.

**TABLE 2:** Computation time (s) for MaxCut on random 10‐regular graphs.

Method	N=50	N=100	N=200	N=400	N=800
KM	2.482±0.030	2.392±0.010	2.578±0.001	2.963±0.050	3.678±0.090
Local Search	0.001±0.0002	0.003±0.0001	0.012±0.0005	0.047±0.0020	0.184±0.0019
Gurobi	0.42±0.23	115.55±29.60	3600	3600	3600

## 3 Discussion and Conclusion

This work develops an analytical framework for understanding how higher‐harmonic forcing, noise, and network structure jointly shape the collective states of oscillator‐based Ising dynamics. Rather than relying solely on numerical simulations, we combine spectral dimension reduction, mean‐field Fokker–Planck analysis, and partition‐function calculations to obtain explicit predictions for stability boundaries, noise‐induced transitions, and topology‐dependent access to low‐energy configurations. These results show that the macroscopic organization of the system is not determined by coupling strength alone, but by a balance among the global interaction strength, the second harmonic term, stochastic fluctuations, and the spectral properties of the underlying network.

The use of dynamical systems for Ising optimization is well established across classical, physical, and quantum‐inspired approaches. For example, mean‐field reductions of quantum optimization algorithms can lead to classical spin dynamics, as demonstrated by the mean‐field approximate optimization algorithm [25]. Our contribution is complementary to these algorithmic developments. Rather than proposing dynamical optimization itself as a new paradigm, we focus on analytically characterizing the critical transitions, noise dependence, and network‐topology effects specific to oscillator‐based Ising dynamics.

A central finding is that the second harmonic interaction provides a binarizing mechanism that links continuous oscillator phases to discrete Ising spins. This term reshapes the energy landscape by favoring phase states near 0 and π, thereby enabling the oscillator network to represent binary configurations. Within this landscape, the deterministic spectral reduction reveals how small perturbations around stationary states relax back to equilibrium and how the parameters K and $K_{s}$ define stability boundaries. The reduced equation for the global observable R provides a compact description of the high‐dimensional dynamics and clarifies why increasing the second harmonic strength can enhance the recovery of stationary states, whereas the effect of the global coupling depends on the network spectrum.

The stochastic analysis further shows that noise plays a dual role. Moderate fluctuations can help the system explore the energy landscape and escape less favorable local configurations, whereas excessive noise disrupts the binary phase structure required for an accurate Ising representation. The mean‐field Fokker–Planck equation predicts a transition between single‐cluster and two‐cluster stationary distributions, with the transition boundary controlled by the competition between the second harmonic forcing and the noise intensity. This provides an analytical explanation for the numerical observation that low‐energy Ising configurations are mainly obtained in parameter regions where the second harmonic term dominates the noise.

Beyond parameter effects, our results highlight the importance of network topology. For the ring‐based topology considered in the partition‐function analysis, adding non‐local links can reshape the number and accessibility of low‐energy configurations. In particular, links to third neighbors lead to a larger number of desired solutions than the other added non‐local connections. This effect is reflected in the spectral properties of the graph, suggesting that appropriate structural modifications can improve the ability of oscillator networks to approach globally favorable configurations. The demonstrations on graph coloring and MaxCut problems further support the practical relevance of these analytical predictions.

There are several limitations to the present study. First, the one‐dimensional spectral reduction is most effective when the dominant collective behavior can be captured by a single real mode. For directed, weighted, or strongly asymmetric networks, complex eigenmodes may contribute substantially to the dynamics, and a one‐dimensional observable may be insufficient. In such cases, multidimensional reductions that retain several relevant spectral modes would be required [42]. Second, the mean‐field Fokker–Planck analysis assumes uniform interactions and therefore provides a tractable but idealized description of the stochastic dynamics. Extending this theory to heterogeneous networks with broad degree distributions, nonuniform coupling strengths, or intrinsic‐frequency disorder will be important for quantitative predictions in more realistic systems. Third, the notion of non‐local links is tied to the ring‐based reference topology considered in the partition‐function analysis. Further work is needed to determine how analogous structural modifications should be defined and whether their observed advantage persists in more general network topologies without a natural notion of locality.

A further limitation is that the present framework focuses on phase‐based oscillator dynamics, in which each node is described by a single phase variable. More general oscillatory systems may involve additional amplitude or multidimensional state variables and can exhibit richer collective behaviors, as found in neuronal and other nonlinear oscillator models [50]. Synchronization in such multidimensional systems has also been analyzed using master stability and Lyapunov approaches [51]. Extending the present spectral‐reduction and stochastic framework to amplitude–phase or multidimensional oscillator systems would require incorporating these additional state variables and their coupling to the phase dynamics. Whether the stability boundaries and topology‐dependent effects identified here persist in such systems remains an important direction for future work.

Several directions follow naturally from these limitations. A first step is to extend the spectral theory to multi‐mode reductions for directed, weighted, and asymmetric networks, where the interaction between several collective modes may determine stability and convergence. A second direction is to develop heterogeneous Fokker–Planck or large‐deviation descriptions that can bridge finite‐size stochastic effects with the infinite‐population theory. A third direction is to systematically quantify how local structural edits, such as link addition or removal, shift spectral properties and reshape the effective energy landscape. A fourth direction is to extend the present framework to amplitude–phase and multidimensional oscillator systems, where additional state variables may modify the stability boundaries and topology‐dependent collective behaviors. Such results could provide design principles for oscillator‐based Ising machines and related physical optimization systems.

## 4 Methods

### 4.1 Spectral Dimension Reduction

In this section, we describe the reduction method [41, 42] used to investigate the system (2). The global observable R is defined as

(12) $$\begin{array}{c} R=a^{⊤}\phi , \end{array}$$

where $a={\left[a_{1},a_{2},\text{…},a_{N}\right]}^{⊤}$ denotes normalized weights of each oscillator, with $\sum_{i}a_{i}=1$. The quantity a determines the relative contribution of each oscillator as a function of its structural features. Although a uniform weighting: $a_{i}=1/N$, can be taken for simplicity, this neglects structural and dynamical differences of individual oscillators. Taking the time derivative in the linear global observable R (12) and taking the governing dynamics (2) into account yield,

(13) $$\dot{R}=-K\sum_{i,j}a_{i}J_{ij}\sin\left(\phi_{i}-\phi_{j}\right)+F\left(\phi \right),$$

where $F\left(\phi \right)=-K_{s}\sum_{i=1}^{N}a_{i}\sin\left(2\phi_{i}\right)$ indicates a weighted sum of the second harmonic terms. The right hand side of Equation (13) can be expanded further around $\phi_{i}=\beta R$ and $\phi_{j}=\gamma R$. The truncated term is taken as $O[{\left(\phi_{i}-\beta R+\phi_{j}-\gamma R\right)}^{2}]$ and is in general close to zero. The time derivative of the global observable, namely $\dot{R}$, leads to self‐consistent equations, and linear terms in the expanded equation will only cancel if

(14) $$\alpha \beta R=\phi^{⊤}Da,$$

(15) $$\alpha \gamma R=\phi^{⊤}J^{⊤}a,$$

where D is a diagonal matrix whose diagonal elements represent the degree of the i‐th node with $D_{ii}=\sum_{j=1}^{N}J_{ij}$. In general, an exact solution of α requires that a satisfies the conditions of Equations (14) and (15). In this case, a should be an eigenvector of both D and $J^{⊤}$, with the corresponding eigenvalues αβ and αγ, respectively. However generally, the matrices D and $J^{⊤}$ will have different eigenvectors.

Let us proceed under the assumption that the dynamics R is *mainly* determined by the underlying network structure. We then *choose* a to be an eigenvector of $J^{⊤}$, i.e., $J^{⊤}a=\lambda a$, where λ=γα is the corresponding eigenvalue. Using $1_{N}^{⊤}a=1$, we have $\lambda =1_{N}^{⊤}J^{⊤}a=a^{⊤}\left(J1_{N}\right)=\alpha$, and this suggests γ=1, i.e., λ=α. As the largest eigenvalue of the matrix J is a key indicator of a network's structure, the weight vector a is treated as the eigenvector of $J^{⊤}$ of the eigenvalue of largest magnitude.

Based on this assignment of the vector a, we solve Equation (14) by using a least squares method. The parameter β is calculated by $\beta^{*}=\arg\min_{\beta }{∥Da-\beta \alpha a∥}^{2}$, with solution

(16) $$\beta^{*}=\frac{1}{\alpha }\frac{a^{⊤}Da}{a^{⊤}a},$$

where β≥1. If each node has the same degree, we obtain $\alpha =d_{i}$, $a_{i}=1/N$, and β=1. For the nonlinear term F(ϕ) in Equation (13), an expression for R cannot be explicitly obtained.

To reduce the dimension of F(ϕ) further, we assume that the system is located in a stable stationary state, written $\phi^{*}$, which is also indicated by the global observable $R^{*}$. Considering small perturbations away from the stationary state, written δϕ, the linearization of the dynamics (13) leads to

(17) $$\begin{array}{c} \dot{\delta }R\approx -\left[K\alpha \left(\beta -1\right)\cos\left(\left(\beta -1\right)R^{*}\right)+2K_{s}\right]\delta R, \end{array}$$

where $\delta R=R-R^{*}$, thus recovering Equation (6).

### 4.2 Solving Fokker–Planck equation

Considering uniform interactions and t→∞, the probability distribution P(ϕ,t) in Equation (8) can be replaced by n(ϕ,t), and the procedures of solving the Fokker–Planck equation of n(ϕ) have been intensively presented [37, 38, 39, 40], Here, we illustrate the main procedures as follows. Assuming the stable stationary state of the system with ∂P/∂t=0, the Fokker–Planck Equation (8) becomes an ordinary equation of n(ϕ). Denoting the average of cosϕ and sinϕ on n(ϕ) as two self‐consistent equations yields

(18) $$\begin{array}{cc} & \mu =\int_{-\pi }^{\pi }d\phi \;\cos\phi \;n\left(\phi \right),\\ & \upsilon =\int_{-\pi }^{\pi }d\phi \;\sin\phi \;n\left(\phi \right). \end{array}$$

The integral term in the Fokker–Planck equation can also be rewritten as $\int_{-\pi }^{\pi }\sin\left(\phi -\phi^{\prime }\right)n\left(\phi^{\prime }\right)=\Psi \sin\left(\phi -\alpha \right)$, where $\Psi =\sqrt{\mu^{2}+\upsilon^{2}}$ and α=arctan(υ/μ). Based on the periodic boundary condition n(ϕ+2π)=n(ϕ), we obtain $n\left(\phi \right)=\Phi^{-1}e^{-G(\phi -\alpha )}$, where $\Phi =\int_{-\pi }^{\pi }d\phi \;e^{-G(\phi )}$ and $G\left(\phi \right)=-\left(K\Psi \cos\phi +\frac{K_{s}}{2}\cos2(\phi +\alpha )\right)/\sigma^{2}$. By using this expression of n(ϕ), we can further solve the self‐consistent equations Equation (18) and obtain ⟨cosϕ⟩=Ψ and ⟨sinϕ⟩=0, where the expectation term is denoted as $\left\langle f\left(\phi \right)\right\rangle =\Phi^{-1}\int_{-\pi }^{\pi }d\phi \;f\left(\phi \right)\exp\left(-G(\phi )\right)$. The function G(ϕ) consists of symmetric and anti‐symmetric parts with $G\left(\phi \right)=G_{s}\left(\phi \right)+G_{a}\left(\phi \right)$, and the anti‐symmetric part $G_{a}\left(\phi \right)=K_{s}\sin\left(2\phi \right)\;\sin\left(2\alpha \right)/\left(2\sigma^{2}\right)$. The expectation of sin(ϕ) can be rewritten as $\left\langle \sin\phi \right\rangle =\Phi^{-1}\int_{0}^{\pi }d\phi \;\sin\phi \;e^{-G(\phi )}\left(1-e^{2G_{a}\left(\phi \right)}\right)$, and $\text{sgn}\left(\left\langle \sin\phi \right\rangle \right)=\text{sgn}\left(G_{a}\left(\phi \right)\right)$, with the sign function sgn(·). Additionally with this solution and with $\text{sgn}(G_{a}\left(\phi \right))=\text{sgn}\left(\sin\alpha \right)$, we have α=0. Therefore, the expression of n(ϕ) is reduced to

(19) $$n\left(\phi \right)=\Phi^{-1}e^{\frac{1}{\sigma^{2}}\left(K\Psi \cos\phi +\frac{K_{s}}{2}\cos\left(2\phi \right)\right)},$$

where $\Phi =\int_{-\pi }^{\pi }d\phi \;e^{\frac{1}{\sigma^{2}}\left(K\Psi \cos\phi +\frac{K_{s}}{2}\cos\left(2\phi \right)\right)}$ and $\Psi =\int_{-\pi }^{\pi }d\phi \;\cos\phi \;n\left(\phi \right)$.

As described in [37, 38, 39, 40], multiple peaks of n(ϕ) indicate a multicluster state as t→∞. The peaks of n(ϕ) are related to the local extreme of n(ϕ), determined by dn/dϕ=0 with

(20) $$\frac{dn}{d\phi }=-\frac{2K_{s}}{\sigma^{2}}\left(\cos\phi +\frac{K\Psi }{2K_{s}}\right)\sin\phi \;n\left(\phi \right).$$

When the term $K\Psi /(2K_{s})>1$, this indicates that n(ϕ) has one maximal value in the interval [0,2π) and the system has a one‐cluster state. When $0<K\Psi /(2K_{s})<1$, n(ϕ) has two maxima at ϕ=0 and ϕ=π, corresponding to two clusters, as well as two minima at $\phi =\arccos\left(-K\Psi /(2K_{s})\right)$ and $\phi =2\pi -\arccos\left(-K\Psi /(2K_{s})\right)$ in the interval [0,2π). Therefore, Equation (9) illustrates the transition line from one to two clusters stationary distribution.

### 4.3 Partition Function of Graph Coloring Problem

The basic idea of the graph coloring problem is to assign a set of m colors, denoted as $c_{1},c_{2},\text{…},c_{m}$, to N nodes of a network with an adjacency matrix A, such that no two adjacent nodes have the same color. The Ising Hamiltonian follows [16, 26, 36, 52, 53, 54, 55]

(21) $$\begin{array}{cc} H & =\sum_{i=1}^{N}{\left(m-2+\sum_{k=1}^{m}s_{ic_{k}}\right)}^{2}+\sum_{(i,j)\in E}^{N_{E}}\sum_{k=1}^{m}A_{ij}\left(1+s_{ic_{k}}\right)\left(1+s_{jc_{k}}\right)\\ & ≜H_{1}+H_{2}, \end{array}$$

where $s_{ic_{k}}$ denotes coloring the i‐th node with the k‐th color. In Equation (21) we have H≥0, $H_{1}\geq 0$ and $H_{2}\geq 0$. The first term, $H_{1}$, characterizes the local coloring properties of each node, and $H_{1}=0$ if there is only one color on each node i with $s_{ic_{k}}=+1$. The second term, $H_{2}$, represents adjacent node coloring properties, and $H_{2}=0$ if two adjacent nodes do not have the same color, with $s_{ic_{k}}=+1$ and $s_{jc_{k}}=+1$ for each pair (i,j)∈E. Based on the correspondence between the oscillator energy and the Ising Hamiltonian in the binary‐phase regime, we construct the corresponding oscillator‐based Ising dynamics for the graph coloring problem as

(22) $${\dot{\phi }}_{i}=-Kh_{i}\sin\phi_{i}-K\sum_{j}J_{ij}\sin\left(\phi_{i}-\phi_{j}\right)-K_{s}\sin\left(2\phi_{i}\right)+\eta_{i}\left(t\right),$$

where the intrinsic magnetic field term, $h_{i}=-\left(m-2\right)-d_{i}$, depends on the degree $d_{i}$ of node i and the number of colors, m, and $\eta_{i}\left(t\right)$ denotes additive noise with zero mean and standard deviation $\sqrt{2}\sigma$. An analytical study of this model in the mean‐field case via a Fokker–Planck equation is given in the Supporting Information, but here we focus on the general case when the network structure is non‐trivial. The network structure and parameter settings are derived via expanding the Ising Hamiltonian (21), and using the relations $s_{i}=\cos\left(\phi_{i}\right)$, $s_{i}s_{j}=\cos\left(\phi_{i}-\phi_{j}\right)$ and $s_{i}^{2}=\cos\left(2\phi_{i}\right)$. The detailed derivation is provided in Sections S6.1 and S6.2. The coupling matrix J is a m×m block matrix, where each diagonal block is −A and the off‐diagonal block corresponds to the identity matrix −I. The resulting oscillator‐based Ising system contains Nm phase oscillators. In a stationary state, the phase of each oscillator will approach 0 or π. When the oscillator dynamics approaches a stationary binary‐phase configuration corresponding to H=0, node i is assigned color k if $\phi_{ic_{k}}=0$. The Ising Hamiltonian $H_{2}$ incorporates the effects of the underlying interactions on the partition function $Z_{2}$, expressed as

(23) $$\begin{array}{cc} Z_{2}= & \sum_{\left\{S_{p}\right\}}e^{-\frac{|H_{2}|}{T}}\\ = & \int_{-\infty }^{\infty }\frac{d\hat{x}}{\pi }\frac{1}{1+{\hat{x}}^{2}}\sum_{\left\{S_{p}\right\}}e^{-i\hat{x}\frac{\sum_{k=1}^{m}\sum_{(i,j)\in E}^{N_{E}}\left(1+s_{ic_{k}}\right)\left(1+s_{jc_{k}}\right)}{T}}. \end{array}$$

To focus on the influence of networks on the Hamiltonian, we first consider a simple ring. In this case, the corresponding Hamiltonian can be rewritten as

$$H_{2}=\sum_{k=1}^{m}\sum_{i=1}^{N}\left(1+s_{ic_{k}}\right)\left(1+s_{i+1\;c_{k}}\right),$$

where $s_{N+1\;c_{k}}\equiv s_{1c_{k}}$.

Additionally, after adding one non‐local link between node 1 and node $i_{0}$, the corresponding form of $H_{2}$ can be expressed as

$$H_{2}=\sum_{k=1}^{m}\sum_{i=1}^{N}\left(1+s_{ic_{k}}\right)\left(1+s_{i+1\;c_{k}}\right)+\left(1+s_{1\;c_{k}}\right)\left(1+s_{i_{0}\;c_{k}}\right).$$

In the low temperature limit, T→0, $Z_{2}$ reduces to

(24) $$\begin{array}{cc} Z_{2}= & \left[\left(c_{1}^{i_{0}}-c_{2}^{i_{0}}\right)\left(c_{1}^{N-i_{0}+2}-c_{2}^{N-i_{0}+2}\right)+2\left(c_{1}^{i_{0}-1}-c_{2}^{i_{0}-1}\right)\right.\\ & {\left.\times \;(c_{1}^{N-i_{0}+1}-c_{2}^{N-i_{0}+1})\right]}^{m}{\left(\frac{1}{5}\right)}^{m}. \end{array}$$

Given that $c_{1}c_{2}=-1$, $(2+c_{1}^{2})/5\approx 1$, and $(2+c_{2}^{2})/5\approx 1/2$, we can further expand the terms on the right‐hand side of the above expression and obtain the approximate form of $Z_{2}$ (see Section S5.2.2).

## Conflicts of Interest

The authors declare no conflicts of interest.

## Supporting information


**Supporting File**: advs78134‐sup‐0001‐SuppMat.pdf.

## Data Availability

Research data are not shared.
