# Supplementary material for: Analytical Prediction of Critical Transitions in Oscillator Networks With Non‐Local Links
Source: Adv Sci (Weinh). 2026 Oct 8:e78134. Online ahead of print. doi: 10.1002/advs.78134 (PMC13650058; doi:10.1002/advs.78134)
Supplement: Supplementary file 1 [file ADVS-9999-e78134-s001.pdf]

Supporting Information
Analytical prediction of critical transitions in oscillator networks with non-local links

Qiang Li, Chumin Sun, David Waxman, Renaud Lambiotte, Jie Sun, Peng Ji*

*Corresponding author. E-mail: pengji@fudan.edu.cn (P. Ji).

1 Deriving the Kuramoto Model from an Autonomous Dynamical System

The autonomous dynamics of a single nonlinear self-sustaining oscillator is [1]

$$\frac{d}{dt}\vec{q}(\vec{x}(t)) + \vec{f}(\vec{x}(t)) = \vec{0}, \tag{1}$$

where $\vec{x}(t) \in \mathbb{R}^N$ represents the state vector, and N is the dimension of the state space. The function $\vec{q}(\cdot)$ is the differential part of the system and represents how the state variables are changing with respect to time. The function $\vec{f}(\cdot)$ is the algebraic part of the system and captures any constraints or relationships that don't involve time derivatives. The term autonomous indicates that the dynamics are solely determined by the current state without explicit dependence on time, which is reflected in the fact that there is no explicit t dependence in the functions $\vec{q}(\cdot)$ and $\vec{f}(\cdot)$.

The oscillator exhibits a time-varying periodic solution in the presence of small perturbations $\vec{b}_j(t) \in \mathbb{R}^N$, $j = 1, \cdots, N$. Utilizing Floquet theory, the system's response is described in terms of a Perturbation Projection Vector (PPV), written $\vec{p}(t)$, which characterizes the oscillator's phase response to external inputs. The locking phenomenon is studied under two scenarios [1]: periodic signals and second-order perturbations. For the T-periodic signals, the phase dynamics are expressed in terms of the phase shift caused by external inputs, yielding Adler's equation under specific conditions,

$$\dot{\phi}(t) = \omega_0 - \omega^* + \omega_0 \sum_{j=0}^{N-1} A_j \sin(\phi_j(t) - \phi(t)), \tag{2}$$

where $\phi(t)$ represents the phase and A_j are couplings between oscillators. The term $\omega_0 - \omega^*$ characterizes the impact of the variability in natural frequency. In the case of the second-order perturbations $\vec{b}_j(2t) = V_j \sin(2t)$ with a constant V_j, and the PPV $\vec{p}(2t)$ is proportional to $\cos(2t)$, the dynamics of the phases are [1]

$$\dot{\phi}(t) = \omega_0 - \omega^* + \frac{\omega_0}{T} \sum_{j=1}^{N} A_j \sin(2(\phi_j(t) - \phi(t))). \tag{3}$$

For a network of oscillators, the phase dynamics are generalized to include contributions from connections, a reference signal, and a second-order harmonic signal [1]. The resulting expression resembles the Kuramoto-type oscillator model for the coupled oscillators,

$$\dot{\phi}_i(t) = \omega_i - \omega^* - \omega_i K \sum_{j=0, j\neq i} J_{ij} \sin(\phi_i(t) - \phi_j(t)) - \omega_i K_s \sin(2\phi_i(t)). \tag{4}$$

The generalized Kuramoto-type oscillator model captures the interactions among oscillators, with terms representing coupling strengths and contributions from different sources of perturbations.

2 Using the Kuramoto-type oscillator model with a second harmonic term to achieve an Ising machine

2.1 Energy Function of Kuramoto Model

The Kuramoto-type oscillator model describes synchronization in a large ensemble of coupled oscillators, which obey

$$\frac{d\phi_i}{dt} = \omega_i - K \sum_{j\neq i} J_{ij} \sin(\phi_i - \phi_j), \tag{5}$$

where ϕ_i is the phase of the i-th oscillator and ω_i is its natural frequency. As described in the main text, J_{ij} describes the coupling interactions between neighboring spins, where positive J_{ij} corresponds to ferromagnetic interactions, whereas negative J_{ij} corresponds to antiferromagnetic interactions. The constant K is the global coupling strength. For the system (5) with randomly initialized phases, the final states are continuously and uniformly synchronized in the interval $(0, 2\pi]$.

The energy function of Eq. (5) is [2]

$$E(\boldsymbol{\phi}(t)) = -\sum_i \omega_i \phi_i(t) - K \sum_{i<j} J_{ij} \cos\left(\phi_i(t) - \phi_j(t)\right). \tag{6}$$

The energy function, $E(\boldsymbol{\phi}(t))$, as a result of Eq. (5), decreases in value over time, as follows. We have

$$\frac{\partial E(\boldsymbol{\phi}(t))}{\partial \phi_k(t)} = K \sum_{l=1, l\neq k}^{N} J_{kl} \sin(\phi_k(t) - \phi_l(t)) - \omega_k = -\dot{\phi}_k(t). \tag{7}$$

Therefore,

$$\frac{dE(\boldsymbol{\phi}(t))}{dt} = -\sum_{k=1}^{N} (\dot{\phi}_k(t))^2 \leq 0. \tag{8}$$

Eq. (8) shows that $E(\boldsymbol{\phi}(t))$ has the decreasing property we would expect in a dissipative system and the coupled Kuramoto oscillators consistently tend to take it downhill to a local minimum $E(\boldsymbol{\phi}(t))$ during the system's evolution [1,3].

In the stationary state ϕ_i^* of the Kuramoto-type oscillator model (5), ϕ_i^* typically converges to continuous values which are uniformly distributed in the phase interval $[0, 2\pi)$. However, in our specific scenario, we introduce a second harmonic term to each oscillator in the network, causing the phases to converge particularly to either 0 or π. Mathematically, we express the Kuramoto-type oscillator model with a second harmonic term as [1,4]

$$\frac{d\phi_i}{dt} = \omega_i - K \sum_{j\neq i} J_{ij} \sin(\phi_i - \phi_j) - K_s \sin(2\phi_i), \tag{9}$$

where K_s is the coupling strength of second harmonic term. In the stationary state $\phi_i^* = 0$ or π, the Jacobi matrix of (9) is

$$Ja_{ij} = \begin{cases} -K \sum_{j\neq i} J_{ij} \cos(\phi_i^* - \phi_j^*) - 2K_s, & i = j, \\ K J_{ij} \cos(\phi_i^* - \phi_j^*), & i \neq j. \end{cases} \tag{10}$$

The energy function of Eq. (9) is

$$E(\boldsymbol{\phi}(t)) = -\sum_i \omega_i \phi_i(t) - K \sum_{i<j} J_{ij} \cos\left(\phi_i(t) - \phi_j(t)\right) - \frac{1}{2} K_s \sum_i \cos\left(2\phi_i\right), \tag{11}$$

where $E(\boldsymbol{\phi}(t))$ satisfies $\frac{dE(\boldsymbol{\phi}(t))}{dt} \leq 0$. At the stationary state of Eq. (9), $\phi_i^* = 0$ or π. We define $s_i = \cos(\phi_i(t))$, near the stationary state, s_i satisfies

$$s_i s_j \approx \cos(\phi_i(t) - \phi_j(t)). \tag{12}$$

By substituting Eq. (12) into Eq. (11) and employing $\cos(2\phi_i) \equiv 1$, Eq. (11) simplifies to

$$E(\boldsymbol{\phi}(t)) = -\sum_{i=1}^{N} \omega_i \phi_i^* - K \sum_{i<j} J_{ij} s_i s_j - \frac{N}{2} K_s \tag{13}$$

We employ a two-dimensional model to illustrate the above statements. Examining a network featuring two nodes, as depicted in Fig. S1 (A), we analyze the dynamics of each node following either Eqs. (5) or (9),

considering both ferromagnetic and antiferromagnetic couplings. Fig. S1 (B) illustrates the energy landscape under ferromagnetic coupling $J_{ij} > 0$, where minimal values are situated along the line defined by $\phi_1 = \phi_2$ within the range $[0, 2\pi) \times [0, 2\pi)$, forming a valley in the energy landscape. The introduction of second harmonic term $K_s \sin(2\phi_i)$ disrupts the valley of minimal values, creating discrete basins centered at $\phi_i^* = 0$ or π (see Fig. S1 (C)). In contrast, an inverted distribution of minimal and maximal values appears in the energy landscape for antiferromagnetic coupling $J_{ij} < 0$, as shown in Fig. S1 (D). The second harmonic term results in minimal energy values concentrating at $\phi_i^* = 0$ or π, with the two phases ϕ_1^* and ϕ_2^* exhibiting a tendency towards a phase difference of π when representing the locations of minimal energy values (see Fig. S1 (E)). Additionally, we investigate the distribution of stationary states for two nodes under varying the parameters K and K_s. The results show that the stationary state ϕ_i^* is a random value when $K_s = 0$, while it converges to 0 or π for $K_s > 0$, and $\phi_i^* = \pi/2$ or $3\pi/2$ for $K_s < 0$ (see Fig. S1 (F)). In our pursuit of realizing an Ising machine through Kuramoto oscillators, we specifically focus on the scenario where $K_s > 0$ in this research.

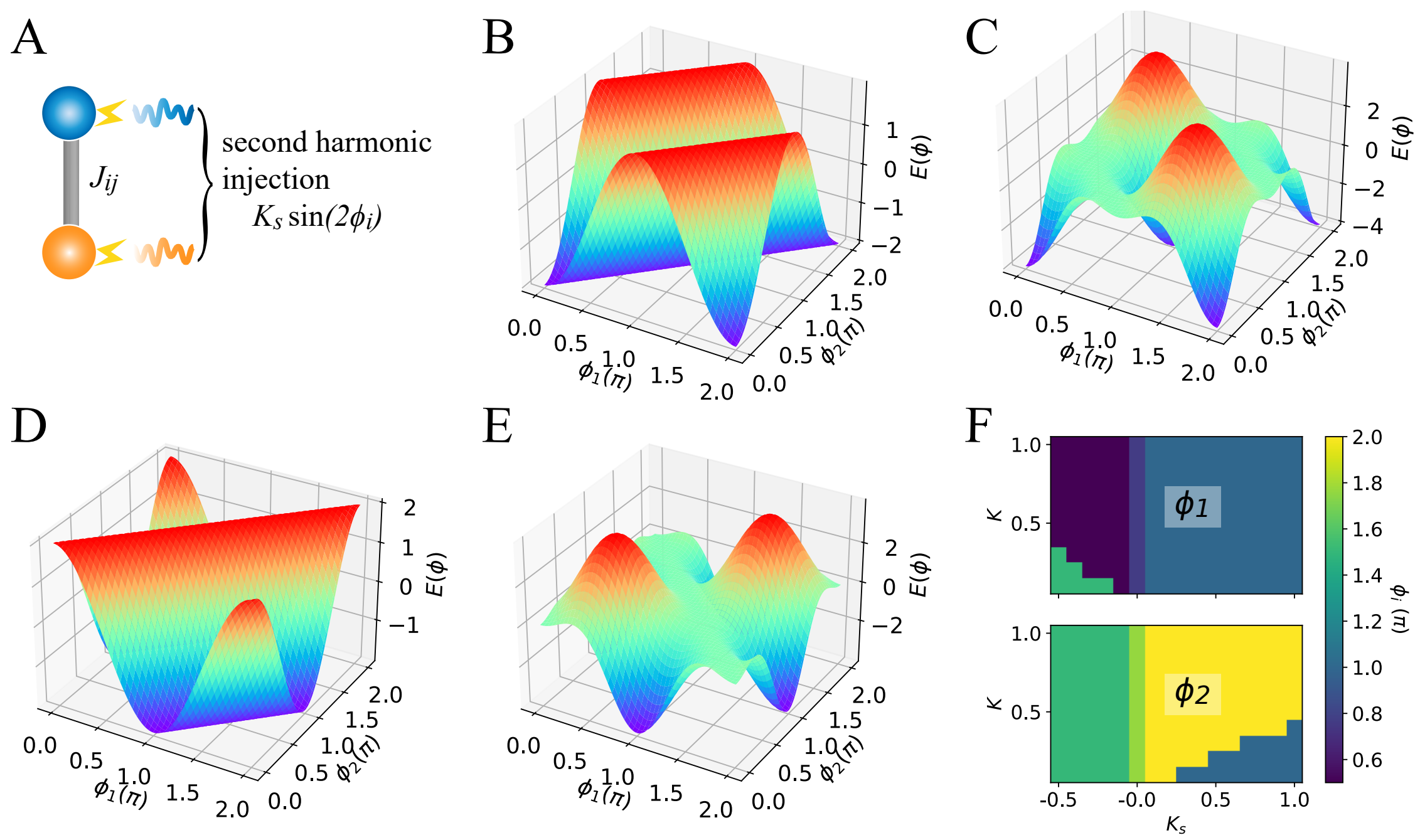

Figure S1. 2-dimensional Kuramoto-type oscillator model to illustrate the effects of the type of magnetic interaction and second harmonic term. (A) A network with 2 nodes, the dynamics of each node is expressed by Eq. (9). The blue and red balls represent the first and second nodes, respectively, and their interactions are denoted as J_{ij}. (B-D) Three-dimensional energy landscape of the network in (A). (B) The ferromagnetic interaction $J_{ij} > 0$ without $K_s \sin(2\phi_i)$, the minimal values of energy locate along the line determined by $\phi_1 = \phi_2$ in the phase regimes $[0, 2\pi) \times [0, 2\pi)$. (C) The ferromagnetic interaction $J_{ij} > 0$ with $K_s \sin(2\phi_i)$, the minimal values of energy satisfy $\phi_1 = \phi_2$ with $\phi_i^* = 0$ or π. (D) The antiferromagnetic interaction $J_{ij} < 0$ without $K_s \sin(2\phi_i)$, the distribution of maximal and minimal energy values is opposite to (B). (E) The antiferromagnetic interaction $J_{ij} < 0$ with $K_s \sin(2\phi_i)$, the locations of minimal values exhibit a binary pattern with $\phi_i^* = 0$ or π, and the connected two nodes tend to have a phase difference of π. (F) The stationary phases distributions of the two connected nodes under different coupling parameters. For simulation, the initial phases are fixed at the same random values. The stationary state ϕ_i^* is a random value for $K_s = 0$, $\phi_i^* = 0$ or π if $K_s > 0$, $\phi_i^* = \pi/2$ or $3\pi/2$ when $K_s < 0$. To achieve Ising machine via Kuramoto oscillators, we consider $K_s > 0$ in this research.

2.2 Effects of natural frequency

Assuming the oscillators are identical, i.e., $\omega_i \equiv \omega$. The parameters ω and K_s determine whether the system is in a stationary or moving state, which motivates us to investigate a special scenario with $J_{ij} \equiv 0$, i.e.,

$$\frac{d\phi}{dt} = \omega - K_s \sin 2\phi. \tag{14}$$

Letting $\frac{d\phi}{dt} = 0$ yields

$$\omega - K_s \sin 2\phi = 0. \tag{15}$$

Eq. (15) has a local minimum at $\phi = 1/2 \arcsin(\omega/K_s)$ if $|\omega/K_s| < 1$, and the corresponding system (14) is located in the stationary state. However, for the case $|\omega/K_s| > 1$, Eq. (15) does not have a local minimum and the system (14) is in a moving state with frequency [2, 5, 6]

$$\tilde{\omega} = 2\pi \left[\int_{-\pi}^{\pi} d\phi \frac{1}{\omega - K_s \sin(2\phi)} \right]^{-1}. \tag{16}$$

In what follows, we use the Kuramoto-type oscillator model with $K_s \sin(2\phi_i)$ to achieve the oscillator-based Ising machine. Our main focus is to analyze how parameters and network structures affect the distribution of stationary states among the oscillators. As a result, we set $\omega_i = 0$ for the subsequent analysis.

2.3 Relationship between the Kuramoto Model and the Ising Model

The dynamical system (9) has two useful characteristics. First, it exhibits binarized stationary states with $\phi_i^* = 0$ or π. Second, its energy function (11) serves as an objective function which the system tends to minimize as it undergoes temporal evolution. These two features suggest the potential utility of this dynamical system as an Ising machine. Ising machines are grounded in the Ising model, which is a mathematical framework that was initially employed to elucidate domain formation in ferromagnets [7]. The Ising model consists of a set of discrete variables s_i, also referred to as spins, each adopting a binary value of ± 1. The Ising model has an "energy function," known as the Ising Hamiltonian,

$$H = -\sum_{i=1}^{N} h_i s_i - \sum_{1 \le i < j \le N} J_{ij} s_i s_j, \tag{17}$$

where N is the number of spins, $\{J_{ij}\}$ and $\{h_i\}$ are real coefficients. The variable s_i takes the value $+1$ or -1 and this denotes the two possible directions, of up and down, of the i-th spin. Extending the dimension of spins from N to $N+1$, denoting $s_{N+1} \equiv +1$, $J_{i,N+1} = J_{N+1,i} = h_i$. The Ising Hamiltonian H can be simplified as

$$H = -\sum_{1 \le i < j \le N+1} J_{ij} s_i s_j. \tag{18}$$

3 Spectral dimension reduction of the Kuramoto-type oscillator model

3.1 Spectral dimension reduction based on adjacency matrix

For the natural frequency $\omega_i = 0$, the Kuramoto-type oscillator model in Eq. (9) is reduced to

$$\frac{d\phi_i}{dt} = -K \sum_{j \ne i} J_{ij} \sin(\phi_i - \phi_j) - K_s \sin(2\phi_i). \tag{19}$$

The model described by Eq.(19) is N dimensional, and the dynamics of nodes are governed by N coupled differential equations. In general, the computational cost of solving N coupled equations becomes large with increasing N. Moreover, the original system comprising N coupled equations is challenging to comprehend and does not yield many general properties of the solutions.

To describe the evolution of the whole system, we introduce a global observable R to reduce the N-dimensional system to be more practical and accessible. Considering a linear observable R of the phases as [8]

$$R = \sum_{i=1}^{N} a_i \phi_i = \boldsymbol{a}^\top \boldsymbol{\phi}, \tag{20}$$

where $a_i \in \mathbb{R}$ is the weight of the i-th node, and $\boldsymbol{a} = [a_1, a_2, \cdots, a_N]^\top$ satisfies

$$\sum_{i=1}^{N} a_i = \mathbf{1}_N \boldsymbol{a} = 1. \tag{21}$$

In a general context, certain components a_i may assume positive or negative values, where the resulting R signifies a weighted global state. In many scenarios, a_i is non-negative, turning the vector $\boldsymbol{a}$ into a probability vector, and R is referred to as a weighted average activity [8].

The linear observable R is a function dependent on the phases ϕ_i for $i = 1, 2, \cdots, N$, and is a one-dimensional variable which characterizes the global activity of the nodes. For example, when $a_i = 1/N$, R reflects variations in the unweighted average phase of the nodes. Despite the simplicity of the unweighted average phase, predicting its value based on the network structure and dynamics is challenging. Consequently, we consider that $\boldsymbol{a}$ should rely on the coupling network structure.

To obtain the evolution of R, we take the time derivative in Eq. (20) and insert Eq. (9), which leads to

$$\dot{R} = F(\boldsymbol{\phi}) - K \sum_{i,j} a_i J_{ij} \sin(\phi_i - \phi_j), \tag{22}$$

where $F(\phi) = -K_s \sum_{i=1}^{N} a_i \sin(2\phi_i)$. We expand the coupling term in Eq. (22) around $\phi_i = \beta R$ and $\phi_j = \gamma R$,

$$\begin{aligned}\sum_{i,j} a_i J_{ij} \sin(\phi_i - \phi_j) \approx& \alpha \sin(\beta R - \gamma R) + \cos(\beta R - \gamma R) \sum_{i,j} a_i J_{ij} (\phi_i - \beta R) \\ &- \cos(\beta R - \gamma R) \sum_{i,j} a_i J_{ij} (\phi_j - \gamma R),\end{aligned} \tag{23}$$

where $\alpha = \sum_{i,j} a_i J_{ij}$ and the second-order terms have been dropped [8]. By omitting the second-order terms, we can obtain an analytical solution, helping to understand the influence of network structure on the global dynamics $\dot{R}$. Additionally, the omitted terms $O[(\phi_i - \beta R + \phi_j - \gamma R)^2]$ depend on the selection of parameters β and γ, along with the weighting vector $\boldsymbol{a}$. In the following analysis, we derive a choice of parameters that minimizes the magnitude of the omitted terms. The left-hand side in Eq. (23) reduces to a function of R if the linear terms cancel out precisely, resulting in two equations which decide the parameters β and γ:

$$\alpha \beta R = \sum_{i,j} a_i J_{ij} \phi_i = \boldsymbol{\phi}^\top D \boldsymbol{a}, \tag{24}$$

$$\alpha \gamma R = \sum_{i,j} a_i J_{ij} \phi_j = \boldsymbol{\phi}^\top J^\top \boldsymbol{a}, \tag{25}$$

where D is an $N \times N$ diagonal matrix with diagonal elements $D_{ii} = d_i^{in} = \sum_{i=1}^{N} J_{ij}$. In fact, due to $R = \boldsymbol{\phi}^\top \boldsymbol{a}$, Eqs. (24) and (25) hold for any $\boldsymbol{\phi}$ yields that $\boldsymbol{a}$ serves as an eigenvector for both matrices $J^\top$ and

D, accompanied by corresponding eigenvalues $\beta\alpha$ and $\gamma\alpha$. While it may not be possible to satisfy Eqs. (24) and (25) simultaneously for an eigenvector $\boldsymbol{a}$, we establish the condition that at least one equation is exactly satisfied.

We select $\boldsymbol{a}$ as an eigenvector of $J^\top$, i.e.,

$$J^\top \boldsymbol{a} = \lambda \boldsymbol{a},$$

where $\lambda = \gamma\alpha$ is the eigenvalue. Using $\mathbf{1}_N^\top \boldsymbol{a} = 1$, we have

$$\lambda = \mathbf{1}_N^\top J^\top \boldsymbol{a} = \boldsymbol{a}^\top (J\mathbf{1}_N) = \alpha,$$

which suggests $\gamma = 1$, i.e., $\lambda = \alpha$. In general, any eigenvectors of $J^\top$ are applicable for dynamical reduction, except those satisfying $\mathbf{1}_N^\top \boldsymbol{a} = 0$. A larger modulus of α indicates a more pronounced impact of the network structure on the weighted activity. When the eigenvalue modulus associated with an eigenvector is small, the resulting global observable R may inadequately represent the network's influence. This inadequacy results in the terms $O[(\phi_i - R)^2]$ being more substantial, compared to eigenvectors with higher modulus. Therefore, selecting $\boldsymbol{a}$ as the eigenvector with the largest eigenvalue modulus signifies its dominance in characterizing the network's influence on R [8].

We solve β in Eq. (24) by using the least square method to minimize the error between the left and right-hand sides,

$$\beta^* = \arg\min_{\beta} \|D\boldsymbol{a} - \beta\alpha\boldsymbol{a}\|^2, \tag{26}$$

where the symbol $\|\cdot\|$ denotes the standard Euclidean norm. Solving Eq. (26) leads to

$$\beta^* = \frac{1}{\alpha}\frac{\boldsymbol{a}^\top D\boldsymbol{a}}{\boldsymbol{a}^\top \boldsymbol{a}}. \tag{27}$$

Eq. (27) implies $\beta^* \geq 1$, and we prove it as follows.

$$\beta^* = \frac{\boldsymbol{a}^\top D\boldsymbol{a}}{\boldsymbol{a}^\top \alpha\boldsymbol{a}} = \frac{\boldsymbol{a}^\top D\boldsymbol{a}}{\boldsymbol{a}^\top J^\top \boldsymbol{a}}, \tag{28}$$

To prove $\beta^* \geq 1$ means that $\boldsymbol{a}^\top D\boldsymbol{a} \geq \boldsymbol{a}^\top J^\top \boldsymbol{a}$. Denoting $L = D - J^\top$, for the ferromagnetic coupling $J_{ij} > 0$, L is the Laplacian matrix. L is a semi-positive matrix, which leads to $\boldsymbol{a}^\top L\boldsymbol{a} = \boldsymbol{a}^\top(D - J^\top)\boldsymbol{a} \geq 0$. For the antiferromagnetic coupling $J_{ij} < 0$, we rewrite Eq. (28) as

$$\beta^* = \frac{\boldsymbol{a}^\top D\boldsymbol{a}}{\boldsymbol{a}^\top \alpha\boldsymbol{a}} = \frac{\boldsymbol{a}^\top(-D)\boldsymbol{a}}{\boldsymbol{a}^\top(-J^\top)\boldsymbol{a}}. \tag{29}$$

Due to the matrix $-J^\top \geq 0$, same as ferromagnetic coupling, we can prove $\boldsymbol{a}^\top D\boldsymbol{a} - \boldsymbol{a}^\top J^\top \boldsymbol{a} \geq 0$. In summary, $\beta^* \geq 1$ for both ferromagnetic and antiferromagnetic couplings.

Substituting $\gamma = 1$ and Eq. (27) into Eq. (23), we have

$$\sum_{i,j} a_i J_{ij} \sin(\phi_i - \phi_j) \approx \alpha \sin(\beta R - R) \tag{30}$$

Substituting Eq.(30) into Eq. (22), we obtain

$$\dot{R} \approx F(\boldsymbol{\phi}) - K\alpha \sin(\beta R - R) \tag{31}$$

Denoting $\boldsymbol{\phi}^* = [\phi_1^*, \phi_2^*, \cdots, \phi_N^*]^\top$ as the stationary state with $\phi_i^* = 0$ or π of the system (19), with corresponding weighted observable

$$R^* = \sum_{i=1}^{N} a_i \phi_i^*. \tag{32}$$

Based on small perturbations $\delta\boldsymbol{\phi}$, i.e., $\boldsymbol{\phi} = \boldsymbol{\phi}^* + \delta\boldsymbol{\phi}$, the evolution of R in Eq. (31) is linearized as

$$\dot{\delta R} \approx -2K_s\delta R - K\alpha(\beta-1)\cos\left((\beta-1)R^*\right)\delta R. \tag{33}$$

where $\delta R = R - R^*$, and we use the fact that $\sin(2\phi_i^*) \equiv 0$ and $\cos(2\phi_i^*) \equiv 1$. The solution of the above equation is

$$\delta R = e^{-c^* t}\delta R(0), \tag{34}$$

where $c^*(K, K_s, R^*) = 2K_s + K\alpha(\beta-1)\cos\left((\beta-1)R^*\right)$ is a constant depending on the stationary state R^* and coupling parameters K and K_s. Eq. (33) establishes a linear balance relation between K and K_s, which ensures the system returns to the original stationary state.

The examination of the dynamics of δR is initially derived from small perturbations. First, we evaluate the correspondence between the analytical expression for δR in Eq. (33) and the simulated δR presented in Eq. (20) for scenarios involving small, medium, and large perturbations. The investigation encompasses Erdős–Rényi (ER) and regular networks, corresponding to $\beta > 1$ and $\beta = 1$, respectively. The analytical expression for δR closely aligns with the simulated δR, and both return to the original stationary states following small and medium perturbations, respectively (refer to Fig. S2 (A) and (B) for the ER network, and Fig. S3 (A) and (B) for the regular network). In the case of the ER network, the analytical δR tends to zero, while the simulated δR synchronizes to another state during large perturbations, indicating a transition to an alternative stationary state. Notably, the analytical δR struggles to capture the global dynamics of the system under the large perturbations (see Fig. S2 (C)). Conversely, for the regular network, both analytical and simulated δR converge to 0 following the large perturbations (see Fig. S3 (C)). Second, we explore the impact of varying parameters K and K_s on the system for small, medium, and large perturbations. Notably, we observe linear balance relations between K and K_s, which facilitates the system returning to the original stationary state following small and medium perturbations (see Fig. S2 (D) and (E) for the ER network, Fig. S3 (D) and (E) for the regular network). However, this linear balance vanishes for the large perturbations in the ER network (see Fig. S2 (F)), whereas it persists for the regular network under the same conditions (see Fig. S3 (F)).

To assess the transient response to perturbations, we exploit the coherency performance metric [9] as

$$H = \int_0^\infty |\delta R(t)|\, dt. \tag{35}$$

The coherency performance metric H quantifies the energy that is dissipated during the system's return to the stationary state. The analytical solution of δR is expressed in Eq. (34), which yields an analytical H as

$$H = |\delta R(0)| \int_0^\infty e^{-c^* t} dt = \frac{|\delta R(0)|}{c^*}, \tag{36}$$

where we assumed $c^* > 0$, i.e., the precondition for Eq. (36) is that the system can return to the original stationary state after the small perturbations.

3.2 Spectral dimension reduction based on the Laplacian matrix

One may think we can linearize Eq. (19) first depending on small perturbations, and derive the dynamics of δR directly. This idea is not feasible as follows.

The system (19) has the stationary state $\boldsymbol{\phi}^* = [\phi_1^*, \phi_2^*, \cdots, \phi_N^*]^\top$ with $\phi_i^* = 0$ or π. Linearizing Eq. (19) around the stationary state yields

$$\dot{\delta\phi}_i(t) = -2K_s\delta\phi_i - K\left[\sum_{j\neq i} J_{ij}\cos(\phi_i^* - \phi_j^*)\delta\phi_i - \sum_{j\neq i} J_{ij}\cos(\phi_i^* - \phi_j^*)\delta\phi_j\right]. \tag{37}$$

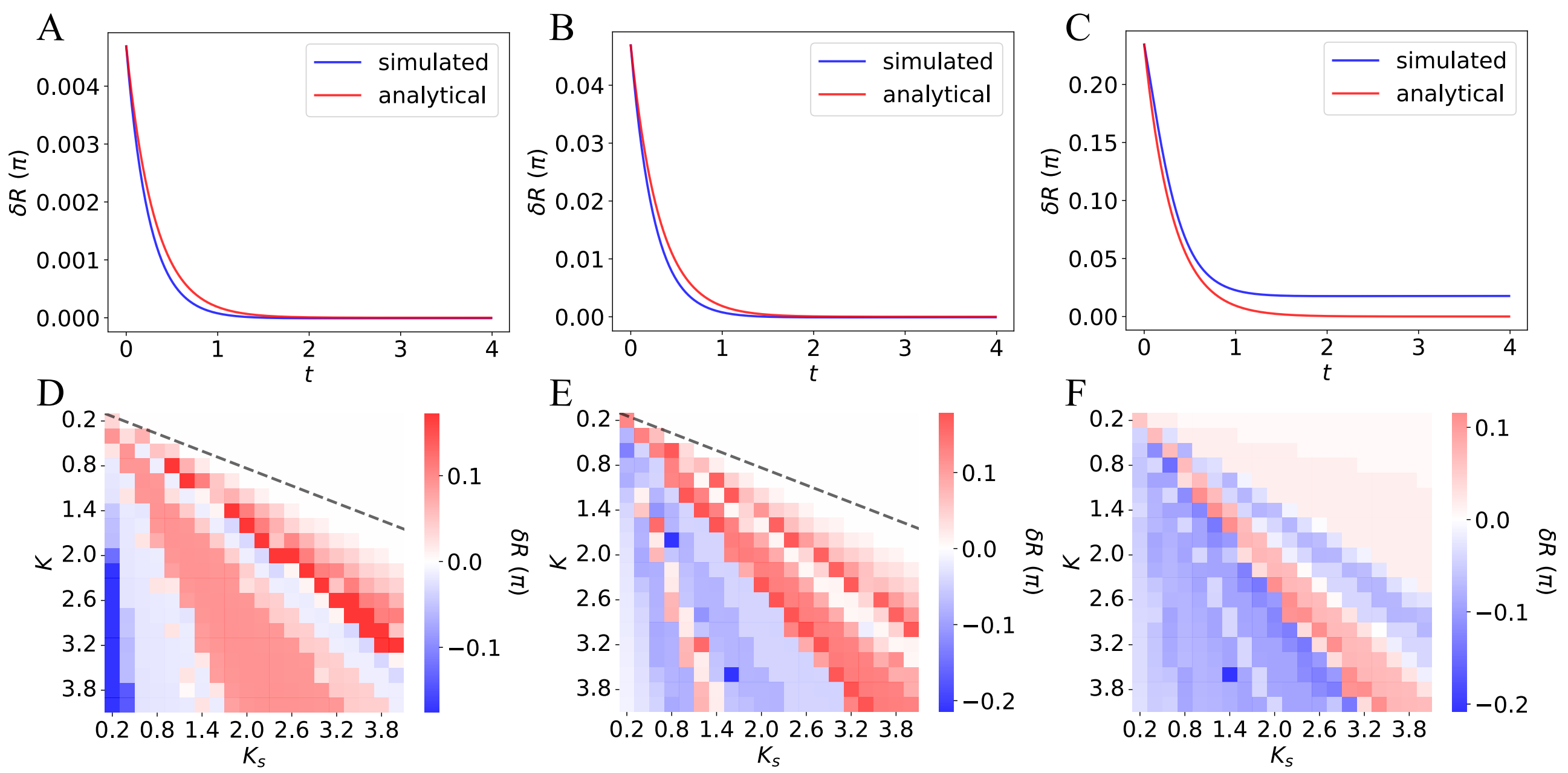

Figure S2. The influence of different magnitudes of perturbations on δR based on an ER network. (A) and (B) illustrate the system returns to the original stationary states after small and medium perturbations, respectively. (C) The system jumps into another stationary state for a large perturbation. The analytical δR predicted by Eq. (33) does not accurately capture the global dynamics of the system. (D) and (E) show the variations of δR with respect to the coupling parameters (K, K_s) for small and medium perturbations, respectively. The gray dashed line represents the linear balance relations between K and K_s. (F) Explores the effects of different parameters (K, K_s) on δR for large perturbations. For simulation, the ER network has $N = 100$ nodes and the connection probability between edges is 0.1. The small, medium, large perturbations are generated from the uniform distribution with intervals $[0, 0.01\pi)$, $[0, 0.1\pi)$, $[0, 0.5\pi)$, respectively.

Introducing the weighted Laplacian matrix L as

$$L_{ij} = \begin{cases} \sum_{k \neq i} J_{ik} \cos(\phi_i^* - \phi_k^*), & i = j, \\ -J_{ij} \cos(\phi_i^* - \phi_j^*), & i \neq j. \end{cases} \tag{38}$$

Substituting L into Eq. (37),

$$\delta \dot{\boldsymbol{\phi}} = -2K_s \delta \boldsymbol{\phi} - KL\delta \boldsymbol{\phi}. \tag{39}$$

By pre-multiplying the vector $\boldsymbol{a}^\top$ in the Eq. (37), we obtain

$$\dot{\delta R} = -2K_s \delta R - K\boldsymbol{a}^\top L \delta \boldsymbol{\phi}. \tag{40}$$

Choosing $\boldsymbol{a}^\top$ is a left eigenvector of L with the largest eigenvalue magnitude,

$$\boldsymbol{a}^\top L = \lambda_m \boldsymbol{a}^\top. \tag{41}$$

In fact, $\boldsymbol{a}$ is also the right eigenvector of $L^\top$ with the largest eigenvalue magnitude. Eq. (40) is rewritten as

$$\dot{\delta R} = -\left(2K_s + K\lambda_m\right) \delta R. \tag{42}$$

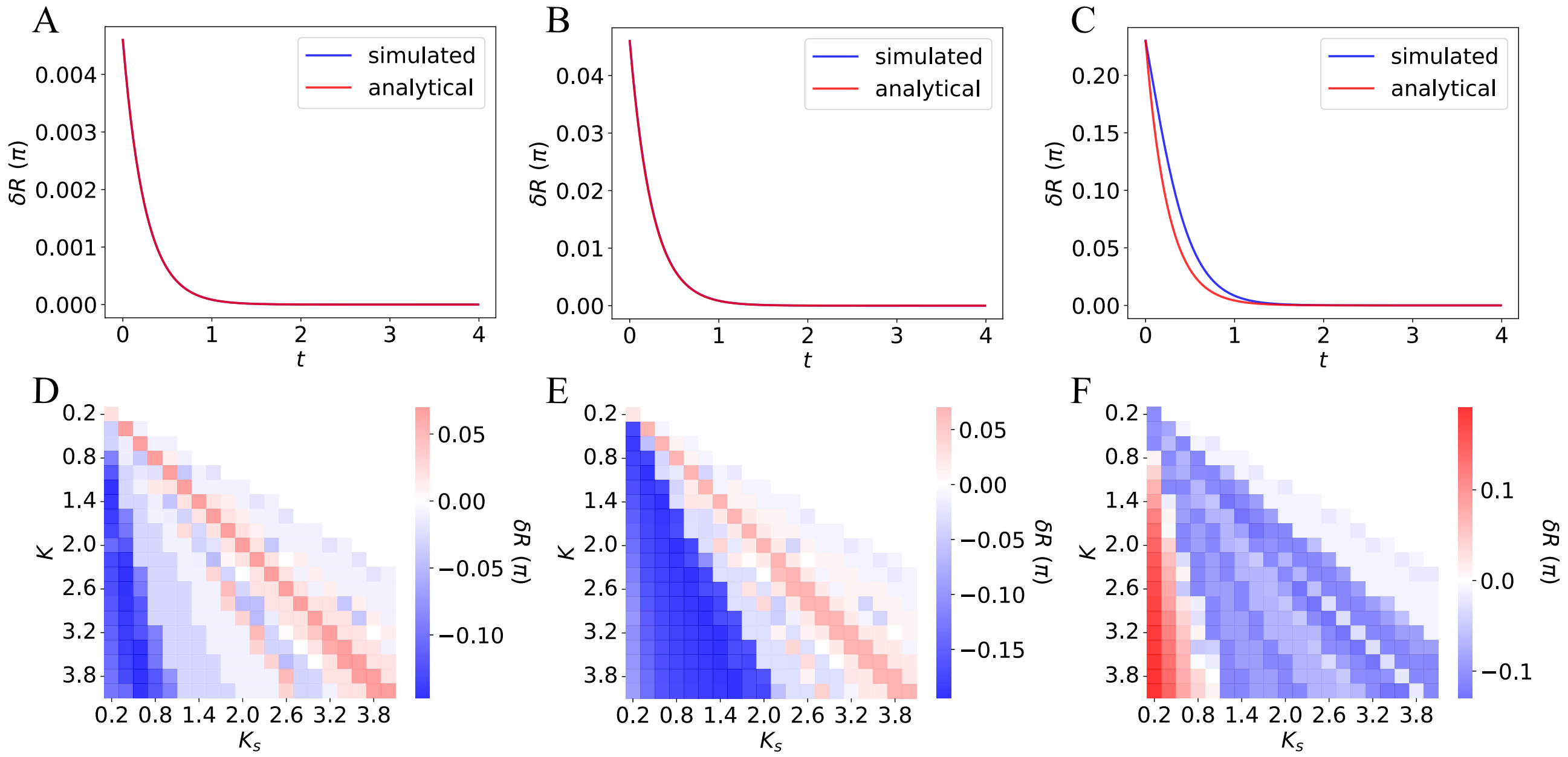

Figure S3. Effects of perturbations with varying magnitudes on δR in a regular network. (A)-(C) illustrate the system returns to the original stationary states after small, medium, and large perturbations, respectively. (D)-(F) show the variations of δR with respect to the coupling parameters (K, K_s) for small, medium and large perturbations, respectively. The regular network consists of $N = 100$ nodes, where each node connects its nearest five neighbors, resulting in a node degree of 10. The small, medium, large perturbations are generated from the uniform distribution with intervals $[0, 0.01\pi)$, $[0, 0.1\pi)$, $[0, 0.5\pi)$, respectively.

The solution of the above equation is

$$\delta R(t) = e^{-(2K_s + K\lambda_m)t}\delta R(0). \tag{43}$$

The above equation implies that the term $2K_s + K\lambda_m$ determines the stability of the stationary state. The weight vector $\boldsymbol{a}$ is an eigenvector of the weighted Laplacian matrix $L^\top$. The definition of L Eq. (38) suggests that the sum of elements in each row of L is 0, i.e., $L\mathbf{1} = \mathbf{0}$. For the transpose of L, we have $\mathbf{1}^\top L^\top = \mathbf{0}^\top$, such that

$$\mathbf{1}^\top L^\top \boldsymbol{a} = \lambda \mathbf{1}^\top \boldsymbol{a} = 0, \tag{44}$$

where λ is the eigenvalue of L with corresponding eigenvector $\boldsymbol{a}$. The above equation yields $\lambda = 0$ or $\boldsymbol{a} = 0$, neither of which satisfies our theoretical analysis.

4 Fokker–Planck equation for the oscillator-based Ising system

The Kuramoto-type oscillator model in Eq. (9) is written as [2,5]

$$\frac{d\phi_i}{dt} = -K_s \sin v\phi_i - K\sum_{j\neq i} J_{ij}\sin(\phi_i - \phi_j), \tag{45}$$

where ϕ_i, $i = 1, 2, \cdots, N$, denotes the phase of the i-th oscillator. In the first term on the right-hand side of Eq. (45), the parameter $K_s > 0$ is referred to as the K_s term in this research. The K_s term can model the dynamics

of stochastic limit-cycle oscillators or excitable elements [2,5]. The positive integer v in the K_s term is typically set to $v = 1$ or $v = 2$. The second term on the right-hand side of Eq. (45) describes coupling interactions with neighbors, which depends on the mutual phase difference among oscillators and the coupling strength J_{ij}.

Assuming uniform coupling, $J_{ij} = 1/N \neq 0$, and additive noise, we rewrite Eq. (45) as the following stochastic differential equation,

$$\frac{d\phi_i}{dt} = -K_s \sin v\phi_i - K \int_{-\pi}^{\pi} d\phi' \sin(\phi_i - \phi') n(\phi', t) + \eta_i(t), \tag{46}$$

where $n(\phi, t) = \frac{1}{N}\sum_{j=1}^{N} \delta(\phi_j(t) - \phi)$ denotes the normalized number density of the oscillators. The term $\eta_i(t)$ represents Gaussian white noise with a mean of 0, and the magnitude of the noise $\eta_i(t)$ is measured by $\sqrt{2}\sigma$.

In the large N limit, the macroscopic behavior of the system (46) is described by a Fokker–Planck equation [10] as

$$\frac{\partial P}{\partial t} = -\frac{\partial}{\partial \phi}\left[\left(-K_s \sin v\phi - K \int_{-\pi}^{\pi} d\phi' \sin(\phi - \phi') n(\phi', t)\right) P\right] + \sigma^2 \frac{\partial^2 P}{\partial \phi^2}, \tag{47}$$

where $P(\phi, t)$ represents the probability distribution of ϕ_i at time t. For uniform interactions, the phases ϕ_i are statistically independent, and the probability distribution $P(\phi, t)$ can be equated to $n(\phi, t)$. Subsequently, we replace $P(\phi, t)$ with $n(\phi, t)$ in Eq. (47), and explore its steady state as $t \to \infty$.

Assuming a stationary state, i.e. $\frac{\partial P}{\partial t} = 0$, the Fokker–Planck equation (47) yields

$$0 = -\frac{d}{d\phi}\left\{\left[-K_s \sin v\phi - K\Psi \sin(\phi - \alpha)\right] n(\phi)\right\} + \sigma^2 \frac{d^2 n(\phi)}{d\phi^2}, \tag{48}$$

where $\Psi = \sqrt{\mu^2 + \tau^2}$, $\alpha = \arctan(\tau/\mu)$. The terms μ and τ are governed by the self-consistent equations

$$\begin{aligned} \mu &= \int_{-\pi}^{\pi} d\phi \, \cos\phi \, n(\phi), \\ \tau &= \int_{-\pi}^{\pi} d\phi \, \sin\phi \, n(\phi). \end{aligned} \tag{49}$$

The solution of Eq. (48) is

$$n(\phi) = e^{-G(\phi-\alpha)}\left[n(0)e^{G(-\alpha)} - \frac{J}{\sigma^2}\int_{-\alpha}^{\phi-\alpha} d\phi' e^{G(\phi')}\right], \tag{50}$$

where the constant J is constrained by the periodic condition $n(\phi + 2\pi) = n(\phi)$, and

$$\begin{aligned} G(\phi) &= -\frac{1}{\sigma^2}\left[\frac{K_s}{v}\cos v(\phi + \alpha) + K\Psi\cos\phi\right] \\ &= -\frac{1}{\sigma^2}\left(\frac{K_s}{v}\cos v\phi \, \cos v\alpha + K\Psi\cos\phi\right) + \frac{K_s}{\sigma^2 v}\sin v\phi \, \sin v\alpha. \end{aligned} \tag{51}$$

The periodic condition $G(\phi + 2\pi) = G(\phi)$ implies that $J = 0$, and yields

$$n(\phi) = \Phi^{-1} e^{-G(\phi-\alpha)}, \tag{52}$$

with $\Phi = \int_{-\pi}^{\pi} d\phi \, e^{-G(\phi)}$. Using the expression of $n(\phi)$ in Eq. (52), the self-consistent equations Eq. (49) are rewritten as

$$\begin{aligned} \Psi\cos\alpha &= \langle\cos\phi\rangle\cos\alpha - \langle\sin\phi\rangle\sin\alpha, \\ \Psi\sin\alpha &= \langle\cos\phi\rangle\sin\alpha + \langle\sin\phi\rangle\cos\alpha, \end{aligned} \tag{53}$$

where the expectation term is denoted as $\langle f(\phi)\rangle = \Phi^{-1}\int_{-\pi}^{\pi} d\phi\, f(\phi)\exp\left(-G(\phi)\right)$ and $f(\phi)$ is a function of ϕ. Solving Eq. (53) yields

$$\langle \cos\phi\rangle = \Psi, \quad \langle \sin\phi\rangle = 0. \tag{54}$$

For convenience of calculation, we separate $G(\phi)$ into symmetric and anti-symmetric parts, $G(\phi) = G_s(\phi) + G_a(\phi)$, with

$$\begin{aligned} G_s(\phi) &= -\frac{1}{\sigma^2}\left(\frac{K_s}{v}\cos v\phi\,\cos v\alpha + K\Psi\cos\phi\right) \\ G_a(\phi) &= \frac{K_s}{\sigma^2 v}\sin v\phi\,\sin v\alpha. \end{aligned} \tag{55}$$

The expectation $\langle \sin\phi\rangle$ is rewritten as

$$\langle \sin\phi\rangle = \Phi^{-1}\int_0^{\pi} d\phi\,\sin\phi\, e^{-G(\phi)}\left[1 - e^{2G_a(\phi)}\right]. \tag{56}$$

Using $\mathrm{sgn}(G_a(\phi)) = \mathrm{sgn}(\sin v\alpha)$, Eq. (56) implies

$$\mathrm{sgn}(\langle\sin\phi\rangle) = \mathrm{sgn}(-\sin v\alpha), \tag{57}$$

where $\mathrm{sgn}(x)$ is the sign function, which is -1 for a negative number, 0 for the number zero, or +1 for a positive number. The self-consistent Eqs. (54) yield $\alpha = 0$, thus $n(\phi)$ is reduced to

$$n(\phi) = \Phi^{-1} e^{\frac{1}{\sigma^2}\left(\frac{K_s}{v}\cos v\phi + K\Psi\cos\phi\right)}, \tag{58}$$

where $\Phi = \int_{-\pi}^{\pi} d\phi\, e^{\frac{1}{\sigma^2}\left(\frac{K_s}{v}\cos v\phi + K\Psi\cos\phi\right)}$ and

$$\Psi = \int_{-\pi}^{\pi} d\phi\,\cos\phi\, n(\phi). \tag{59}$$

As described in [2,5,6], multiple peaks of $n(\phi)$ indicate multiple clusters in the oscillator distribution when $t \to \infty$. The peaks of $n(\phi)$ correspond to its local extrema and are determined by $\frac{dn}{d\phi} = 0$,

$$\frac{dn}{d\phi} = -\frac{1}{\sigma^2}(K_s\sin v\phi + K\Psi\sin\phi)n(\phi). \tag{60}$$

For the case of $v = 1$, Eq. (60) reduces to

$$\frac{dn}{d\phi} = -\frac{1}{\sigma^2}(K_s + K\Psi)\sin\phi\, n(\phi). \tag{61}$$

For the positive coupling, i.e., $K > 0$, Eq. (61) yields $n(\phi)$ has a maximal value at $\phi = 0$ and a minimal value at $\phi = \pi$ in the interval $[0, 2\pi)$, implying the system has a stable equilibrium at $\phi = 0$. Fig. S4 (A) shows the analytical distribution of $n(\phi)$ corresponding to $v = 1$, and we take some representative values of σ and K_s by setting $K = 1$. The results show that $n(\phi)$ has a peak at $\phi = 0$, implying the system has one stationary cluster located in $\phi = 0$.

For the case of $v = 2$, Eq. (60) is written as

$$\frac{dn}{d\phi} = -\frac{2K_s}{\sigma^2}(\cos\phi + \frac{K\Psi}{2K_s})\sin\phi\, n(\phi). \tag{62}$$

For the term $K\Psi/(2K_s) > 1$, Eq. (62) yields $n(\phi)$ has a maximal value at $\phi = 0$ and a minimal value at $\phi = \pi$ in the interval $[0, 2\pi)$, implying the system has a stable equilibrium at $\phi = 0$. However, when the

term $0 < K\Psi/(2K_s) < 1$, $n(\phi)$ has two maximal values at $\phi = 0$ and $\phi = \pi$, as well as two minimal values at $\phi = \arccos(-K\Psi/(2K_s))$ and $\phi = 2\pi - \arccos(-K\Psi/(2K_s))$ in the interval $[0, 2\pi)$, implying the system has two stable equilibria at $\phi = 0$ and $\phi = \pi$. Fig. S4 (B) shows the analytical distribution of $n(\phi)$ with $K = 1$ for the case $v = 2$. The results show that $n(\phi)$ has a peak at $\phi = 0$ in green and golden lines with $K\Psi/(2K_s) > 1$, indicating the system has one stationary cluster located in $\phi = 0$. However, the blue and red lines with $0 < K\Psi/(2K_s) < 1$ show that $n(\phi)$ has two peaks at $\phi = 0$ and $\phi = \pi$, suggesting the oscillators tend to separate into two stationary clusters located at $\phi = 0$ and $\phi = \pi$ when $t \to \infty$. Consequently, Eq.(62) leads to the transitions at $K_s^c = K\Psi/2$ for $v = 2$, and Ψ is the function of K_s, K and σ. While for $K_s < K_s^c$, $n(\phi)$ has one cluster, for $K_s > K_s^c$, $n(\phi)$ has two clusters. The analytical transition line determined by $K_s^c = K\Psi/2$ with $K = 1$ is shown in Fig. S5. In the lower left of the transition line, the system exhibits one stationary cluster. However, the system prefers to separate into two stationary clusters in the upper right of the transition line.

To better determine the number of clusters when the system reaches a stationary state, we define

$$S_c = \frac{1}{4}\sum_{i,j}(1 - \cos(\phi_i)\cos(\phi_j)) = \frac{1}{4}\sum_{i,j}(1 - s_i s_j). \tag{63}$$

The measurement $S_c \geq 0$ due to $1 - \cos(\phi_i)\cos(\phi_j) \geq 0$ for all kinds of stationary cluster distributions. Based on different combinations of the parameters K_s and σ^2, the stationary states are one or two clusters. The measurement $S_c = 0$ implies the stationary states belong to one cluster. Assuming that one node deviates from the original cluster while all remaining nodes belong to the same cluster, we can obtain the threshold $\overline{S_{th}}$ for distinguishing the one-cluster stationary state. Without loss of generality, assuming that the i_0-th node deviates from the original cluster, and the remaining other nodes belong to the same cluster,

$$\overline{S_{th}} = \frac{1}{4}\left[\sum_{j\neq i_0}(1 - \cos(\phi_{i_0})\cos(\phi_j)) + \sum_{j\neq i_0}(1 - \cos(\phi_j)\cos(\phi_{i_0}))\right] = N - 1,$$

where we have used the fact that $1 - \cos\phi_{i_0}\cos\phi_j = 2$. Therefore, $S_c = 0$ if the stationary states belong to one cluster and $S_c \geq N - 1$ otherwise.

5 Partition function of an Ising Hamiltonian

5.1 Ising Hamiltonian of the graph coloring problem

First, we introduce the Ising Hamiltonian of graph coloring problem. We use m colors $\{c_1, c_2, \cdots, c_m\}$ to color n nodes. For each node i, we assign m spins $s_{ic_1}, s_{ic_2}, \cdots, s_{ic_m}$ to represent its coloring scheme, when only one of them is $+1$, the node is successfully colored as color c_k. Then we write an energy function H associated with the nm spins as [1, 4]

$$H = \sum_{i=1}^{n}(m - 2 + \sum_{k=1}^{m} s_{ic_k})^2 + \sum_{(i,j)\in\mathbb{E}}^{N_\mathbb{E}}\sum_{k=1}^{m}(1 + s_{ic_k})(1 + s_{jc_k}), \tag{64}$$

where n is the number of vertices, $\mathbb{E}$ represents the edge set, $N_\mathbb{E}$ is the number of edges. The first term of H is a sum of squares and is non-negative. It reaches zero only when exactly one $s_{ic_k} = +1$ for each i, i.e., when each node has a unique color. The latter term is also non-negative because each spin takes a value in {-1,+1}. It is zero when $s_{ic_k} = s_{jc_k} = +1$ never occurs for any edge connecting i and j and for any color c_k, i.e., when adjacent nodes do not share the same color. When H reaches its minimum value of 0, the spin configuration represents a valid coloring scheme. We follow the indices of $+1$ spins $i, c_k | s_{ic_k} = +1$ and assign color c_k to node i.

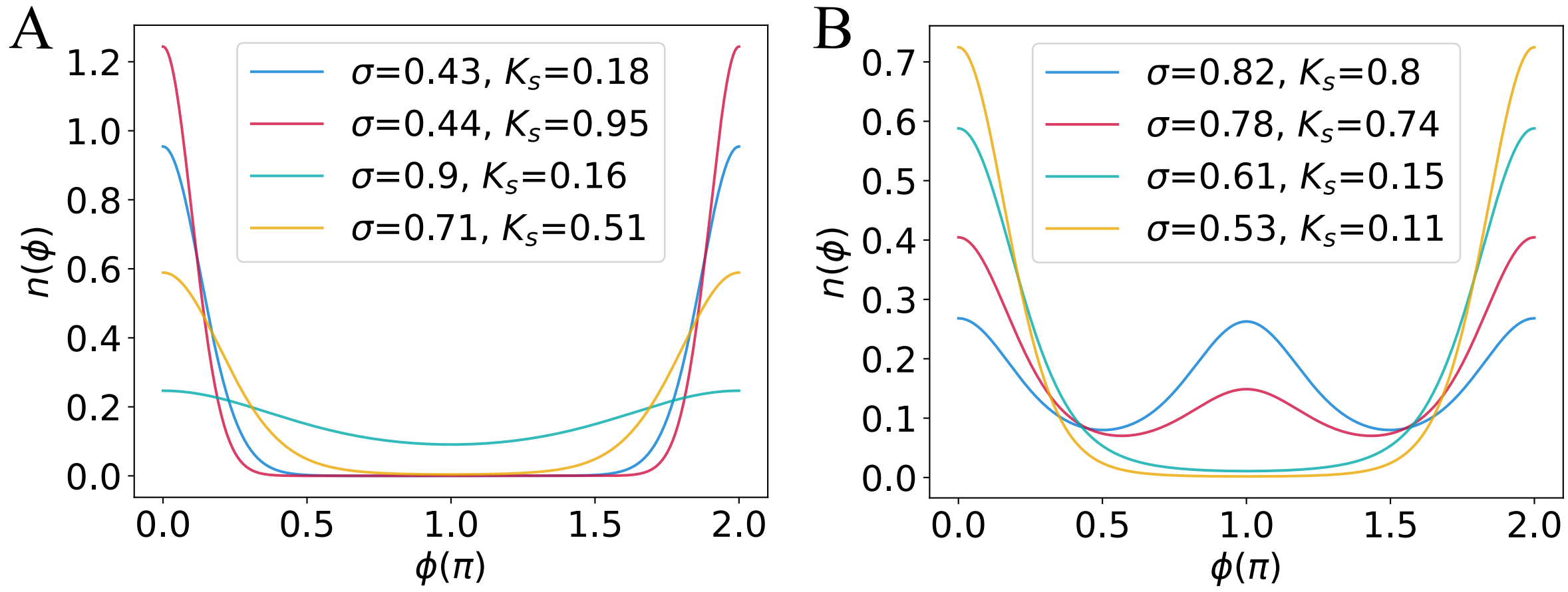

Figure S4. Analytical distribution of $n(\phi)$. (A) $v = 1$. Some representative parameters σ and K_s are used, i.e., both σ and K_s are small (blue line), σ is small but K_s is large (red line), σ is large but K_s is small (green line), both σ and K_s take intermediate values (golden line). (B) $v = 2$. Two representative groups of the parameters σ and K_s are used to illustrate whether $n(\phi)$ has one or two peaks. The blue and red lines show $n(\phi)$ has two peaks for $\phi \in [0, 2\pi)$, while the green and golden lines display one peak. In these analytical lines of $n(\phi)$, the coupling strength K is set to 1 to solve Eq. (58).

5.2 Partition function of an Ising Hamiltonian for the graph coloring problem

For the Ising Hamiltonian in Eq. (64), we denote the two terms by $H_1 = \sum_{i=1}^{n}(m - 2 + \sum_{k=1}^{m} s_{ic_k})^2$, $H_2 = \sum_{(i,j)\in\mathbb{E}}^{N_\mathbb{E}} \sum_{k=1}^{m}(1 + s_{ic_k})(1 + s_{jc_k})$, which characterize the effects of the number of colors and the underlying network structure, respectively. We write H as

$$H = H_1 + H_2. \tag{65}$$

In this research, we mainly focus on the influence of the underlying network structure, while keeping the number of colors constant at m. Specifically, we investigate the variations of H_2 with respect to different network structures. The partition function of H_2 is

$$\begin{aligned} Z_2 = \sum_{\{S_p\}} e^{-\frac{H_2}{T}} &= \sum_{\{S_p\}} e^{-\frac{\left|\sum_{k=1}^{m}\sum_{(i,j)\in\mathbb{E}}^{N_\mathbb{E}}(1+s_{ic_k})(1+s_{jc_k})\right|}{T}} \\ &= \int_{-\infty}^{\infty} \frac{d\hat{x}}{\pi} \frac{1}{1+\hat{x}^2} \sum_{\{S_p\}} e^{-\mathrm{i}\hat{x}\frac{\sum_{k=1}^{m}\sum_{(i,j)\in\mathbb{E}}^{N_\mathbb{E}}(1+s_{ic_k})(1+s_{jc_k})}{T}}, \end{aligned} \tag{66}$$

where we use $H_2 \geq 0$, i.e., $H_2 = |H_2|$. In what follows, we consider the specific network structures to solve the partition function.

5.2.1 Coloring a ring with n nodes

Case 1 Coloring n nodes with ring connections, and the corresponding H_2 in Hamiltonian is

$$H_2 = \sum_{k=1}^{m}\sum_{i=1}^{n}(1 + s_{ic_k})(1 + s_{i+1\,c_k}), \tag{67}$$

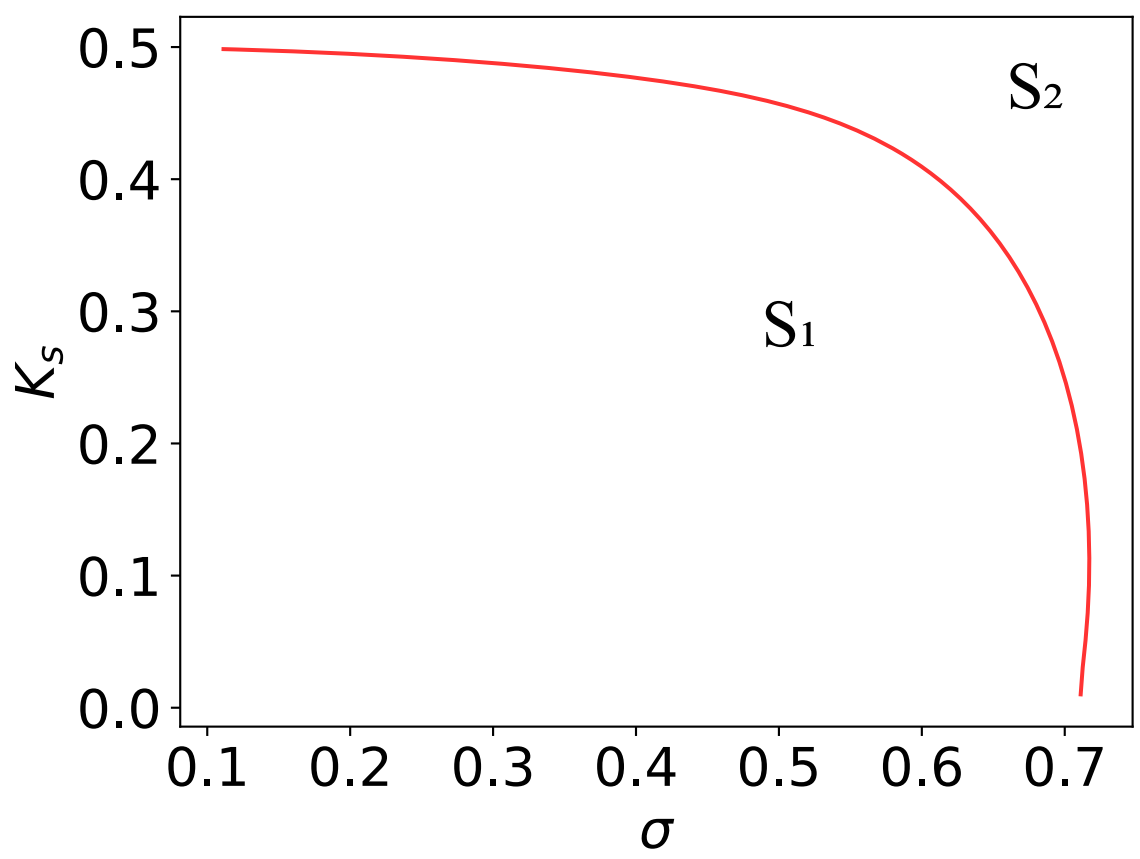

Figure S5. Analytical transition line determined by $K_s^c = K\Psi/2$. The notations S_1 and S_2 represent stationary phase distribution with one and two clusters, respectively. The coupling strength is set to $K = 1$.

where $s_{n+1\,c_k} \equiv s_{1c_k}$. The corresponding partition function is

$$Z_2 = \int_{-\infty}^{\infty} \frac{d\hat{x}}{\pi} \frac{1}{1+\hat{x}^2} \sum_{\{S_p\}} \prod_{k=1}^{m} e^{-\mathrm{i}\hat{x}\frac{\sum_{i=1}^{n}(1+s_{ic_k})(1+s_{i+1\,c_k})}{T}}$$
$$= \int_{-\infty}^{\infty} \frac{d\hat{x}}{\pi} \frac{1}{1+\hat{x}^2} \prod_{k=1}^{m} \sum_{\{s_{ic_k}=\pm1\}} e^{-\mathrm{i}\hat{x}\frac{\sum_{i=1}^{n}(1+s_{ic_k})(1+s_{i+1\,c_k})}{T}}. \tag{68}$$

To solve $\sum_{\{s_{ic_k}=\pm1\}} e^{-\mathrm{i}\hat{x}\frac{\sum_{i=1}^{n}(1+s_{ic_k})(1+s_{i+1\,c_k})}{T}}$, we define a matrix

$$M = \begin{pmatrix} M_{-1,-1} & M_{-1,1} \\ M_{1,-1} & M_{1,1} \end{pmatrix}, \tag{69}$$

where $M_{p,q} = e^{-\mathrm{i}\hat{x}\frac{(1+p)(1+q)}{T}}$, with $p, q \in \{-1, 1\}$. Using $\sum_{\{s_{ic_k}=\pm1\}} e^{-\mathrm{i}\hat{x}\frac{\sum_{i=1}^{n}(1+s_{ic_k})(1+s_{i+1\,c_k})}{T}} = \mathrm{Tr}(M^n)$, we rewrite Z_2 as

$$Z_2 = \int_{-\infty}^{\infty} \frac{d\hat{x}}{\pi} \frac{1}{1+\hat{x}^2} \prod_{k=1}^{m} \mathrm{Tr}(M^n). \tag{70}$$

The number of perfects equals the value of the partition function Z_2 in the temperature limitation $T \to 0$. From the definition $Z_2 = \sum_{\{S_p\}} e^{-\frac{H_2}{T}}$, the spins $\vec{s} \in \{S_p\}$ with $H_2 > 0$ leads to the sum terms in Z_2 tends to 0 when $T \to 0$. The element $M_{1,1}$ characterizes both two connected spins are 1, which leads to $H_2 > 0$ and $e^{-\frac{H_2}{T}} \to 0$ when $T \to 0$, and we obtain

$$M = \begin{pmatrix} 1 & 1 \\ 1 & 0 \end{pmatrix}. \tag{71}$$

The two eigenvalues of M are $\lambda_1 = \frac{1+\sqrt{5}}{2}$, $\lambda_2 = \frac{1-\sqrt{5}}{2}$, using $\mathrm{Tr}(M^n) = \lambda_1^n + \lambda_2^n$, we obtain the number of perfects with $H_2 = 0$ is

$$Z_2 = \left[\left(\frac{1+\sqrt{5}}{2}\right)^n + \left(\frac{1-\sqrt{5}}{2}\right)^n\right]^m. \tag{72}$$

The probability of perfects with $H_2 = 0$ is

$$P_{\text{perf}} = \left[\left(\frac{1+\sqrt{5}}{4} \right)^n + \left(\frac{1-\sqrt{5}}{4} \right)^n \right]^m . \tag{73}$$

We show the variations of P_{perf} with respect to n for $m = 3$ and $m = 4$ in Fig. S6 (A) and (B), respectively.

Using the permutation and combination method, we obtain the probability of perfects with Hamiltonian $H = 0$ is

$$P_{\text{perf}} = \left(\frac{m-1}{2^m} \right)^n + (m-1) \left(-\frac{1}{2^m} \right)^n . \tag{74}$$

We show P_{perf} with $H = 0$ for $m = 3$ and $m = 4$ in Fig. S6 (C) and (D), respectively.

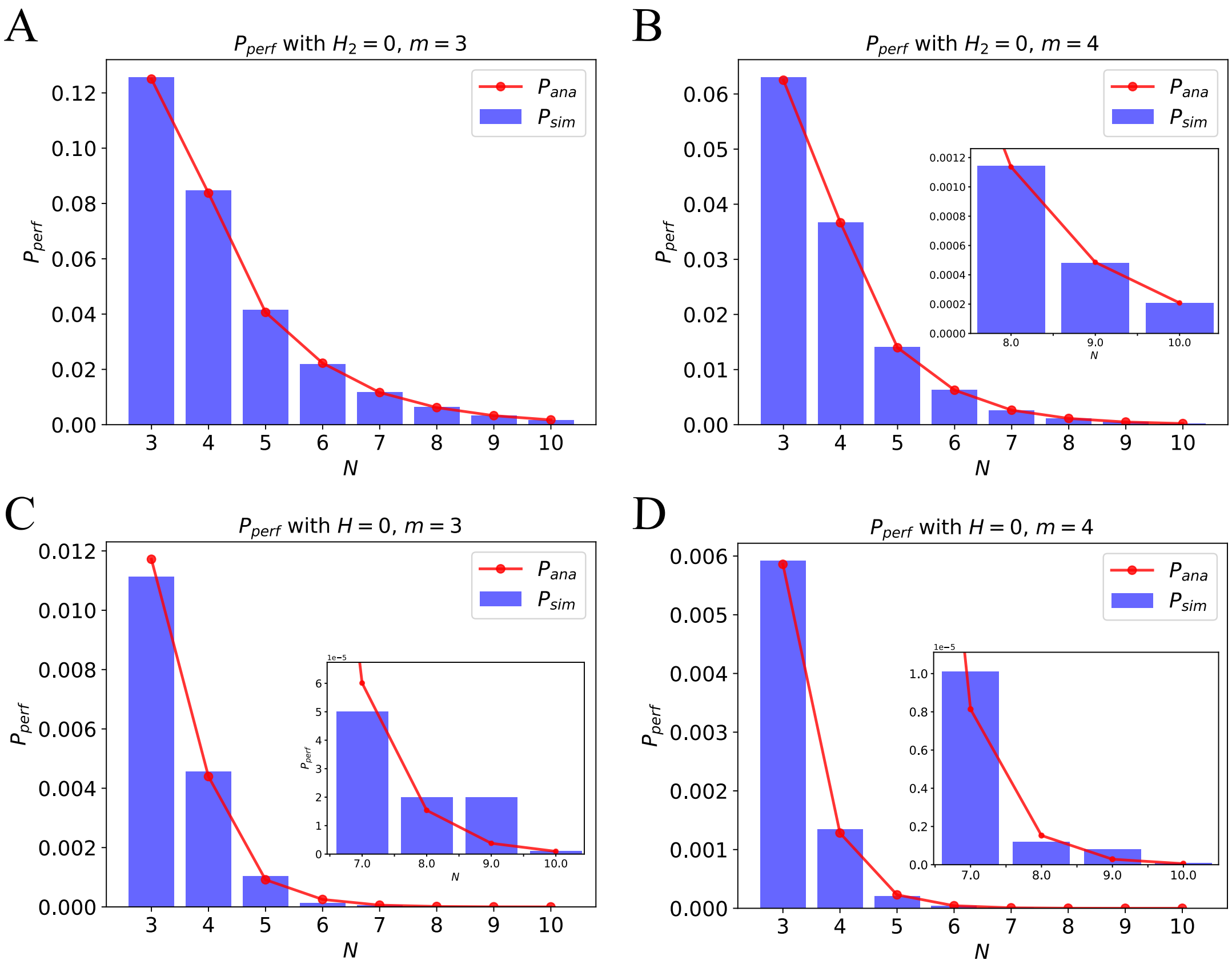

Figure S6. The probability of perfects P_{perf} with respect to n. (A-B) The probability P_{perf} with $H_2 = 0$, which is determined by Eq. (73) and obtained by the partition function method. (C-D) The probability P_{perf} with $H = 0$, which is expressed by Eq. (74) and obtained via the permutation and combination method. (A) and (C), (B) and (D), depend on different color numbers, $m = 3$ and $m = 4$, respectively. The simulation results are generated by the Monte Carlo method. We generate large number of Ising spins s_{ic_k} and repeat simulations numerous times, and count the number of simulations such that the Ising Hamiltonian $H_2 = 0$ (for (A) and (B)) or $H = 0$ (for (C) and (D)).

5.2.2 Coloring a ring of n nodes plus one non-local link

Case 2 Coloring a ring of n nodes plus one non-local link, with the corresponding H_2 in Hamiltonian

$$H_2 = \sum_{k=1}^{m}\left[\sum_{i=1}^{n}(1+s_{ic_k})(1+s_{i+1\,c_k}) + (1+s_{1\,c_k})(1+s_{i_0\,c_k})\right]. \quad (75)$$

The partition function of H_2 is

$$\begin{aligned} Z_2 &= \int_{-\infty}^{\infty}\frac{d\hat{x}}{\pi}\frac{1}{1+\hat{x}^2}\prod_{k=1}^{m}\sum_{\{s_{ic_k}=\pm1\}} e^{-\mathrm{i}\hat{x}\frac{\sum_{i=1}^{n}(1+s_{ic_k})(1+s_{i+1\,c_k})+(1+s_{1,c_k})(1+s_{i_0,c_k})}{T}} \\ &= \int_{-\infty}^{\infty}\frac{d\hat{x}}{\pi}\frac{1}{1+\hat{x}^2}\prod_{k=1}^{m}\Big[\left(M^{i_0-1}\right)_{-1,-1}\left(M^{n-i_0+1}\right)_{-1,-1} + \\ &\quad \left(M^{i_0-1}\right)_{-1,1}\left(M^{n-i_0+1}\right)_{-1,1} + \left(M^{i_0-1}\right)_{1,-1}\left(M^{n-i_0+1}\right)_{1,-1}\Big]. \end{aligned} \quad (76)$$

The eigen-decomposition of M is

$$M = \begin{pmatrix} \frac{1+\sqrt{5}}{\sqrt{10+2\sqrt{5}}} & \frac{1-\sqrt{5}}{\sqrt{10-2\sqrt{5}}} \\ \frac{2}{\sqrt{10+2\sqrt{5}}} & \frac{2}{\sqrt{10-2\sqrt{5}}} \end{pmatrix}\begin{pmatrix}\lambda_1 & \\ & \lambda_2\end{pmatrix}\begin{pmatrix} \frac{1+\sqrt{5}}{\sqrt{10+2\sqrt{5}}} & \frac{2}{\sqrt{10+2\sqrt{5}}} \\ \frac{1-\sqrt{5}}{\sqrt{10-2\sqrt{5}}} & \frac{2}{\sqrt{10-2\sqrt{5}}} \end{pmatrix}, \quad (77)$$

where the two eigenvalues of M are $\lambda_1 = \frac{1+\sqrt{5}}{2}$ and $\lambda_2 = \frac{1-\sqrt{5}}{2}$. Using the orthonormality of the eigenvectors, we have

$$M^{i_0-1} = \begin{pmatrix} \frac{(1+\sqrt{5})^{i_0+1}}{2^{i_0-1}(10+2\sqrt{5})} + \frac{(1-\sqrt{5})^{i_0+1}}{2^{i_0-1}(10-2\sqrt{5})} & \frac{(1+\sqrt{5})^{i_0}}{2^{i_0-2}(10+2\sqrt{5})} + \frac{(1-\sqrt{5})^{i_0}}{2^{i_0-2}(10-2\sqrt{5})} \\ \frac{(1+\sqrt{5})^{i_0}}{2^{i_0-2}(10+2\sqrt{5})} + \frac{(1-\sqrt{5})^{i_0}}{2^{i_0-2}(10-2\sqrt{5})} & \frac{(1+\sqrt{5})^{i_0-1}}{2^{i_0-3}(10+2\sqrt{5})} + \frac{(1-\sqrt{5})^{i_0-1}}{2^{i_0-3}(10-2\sqrt{5})} \end{pmatrix}, \quad (78)$$

i.e., $\left(M^{i_0-1}\right)_{-1,1} = \left(M^{i_0-1}\right)_{1,-1}$ for any i_0. We reduce Z_2 to

$$Z_2 = \left[(c_1^{i_0} - c_2^{i_0})(c_1^{n-i_0+2} - c_2^{n-i_0+2}) + 2(c_1^{i_0-1} - c_2^{i_0-1})(c_1^{n-i_0+1} - c_2^{n-i_0+1})\right]^m \left(\frac{1}{5}\right)^m. \quad (79)$$

where $c_1 = \frac{1+\sqrt{5}}{2}$, $c_2 = \frac{1-\sqrt{5}}{2}$. The probability of perfects with $H_2 = 0$ is

$$\begin{aligned} P_{\text{perf}} = \Bigg\{ &\frac{4}{5}\left[\left(\frac{1+\sqrt{5}}{4}\right)^{i_0} - \left(\frac{1-\sqrt{5}}{4}\right)^{i_0}\right]\left[\left(\frac{1+\sqrt{5}}{4}\right)^{n-i_0+2} - \left(\frac{1-\sqrt{5}}{4}\right)^{n-i_0+2}\right] + \\ &\frac{2}{5}\left[\left(\frac{1+\sqrt{5}}{4}\right)^{i_0-1} - \left(\frac{1-\sqrt{5}}{4}\right)^{i_0-1}\right]\left[\left(\frac{1+\sqrt{5}}{4}\right)^{n-i_0+1} - \left(\frac{1-\sqrt{5}}{4}\right)^{n-i_0+1}\right]\Bigg\}^m. \end{aligned} \quad (80)$$

For varying n and m, we show P_{perf} corresponding to $H_2 = 0$ as a function of i_0 in Fig. S7. For large n, because P_{perf} is too small, we only display the analytical results predicted by Eq. (80) in Fig. S8.

5.3 Partition function of Hamiltonian

5.3.1 A ring with n nodes

Considering a ring with n nodes, and the corresponding Hamiltonian is

$$H = \left|\sum_{i=1}^{n}\sum_{j=1}^{n} a_{ij}s_is_j\right|. \quad (81)$$

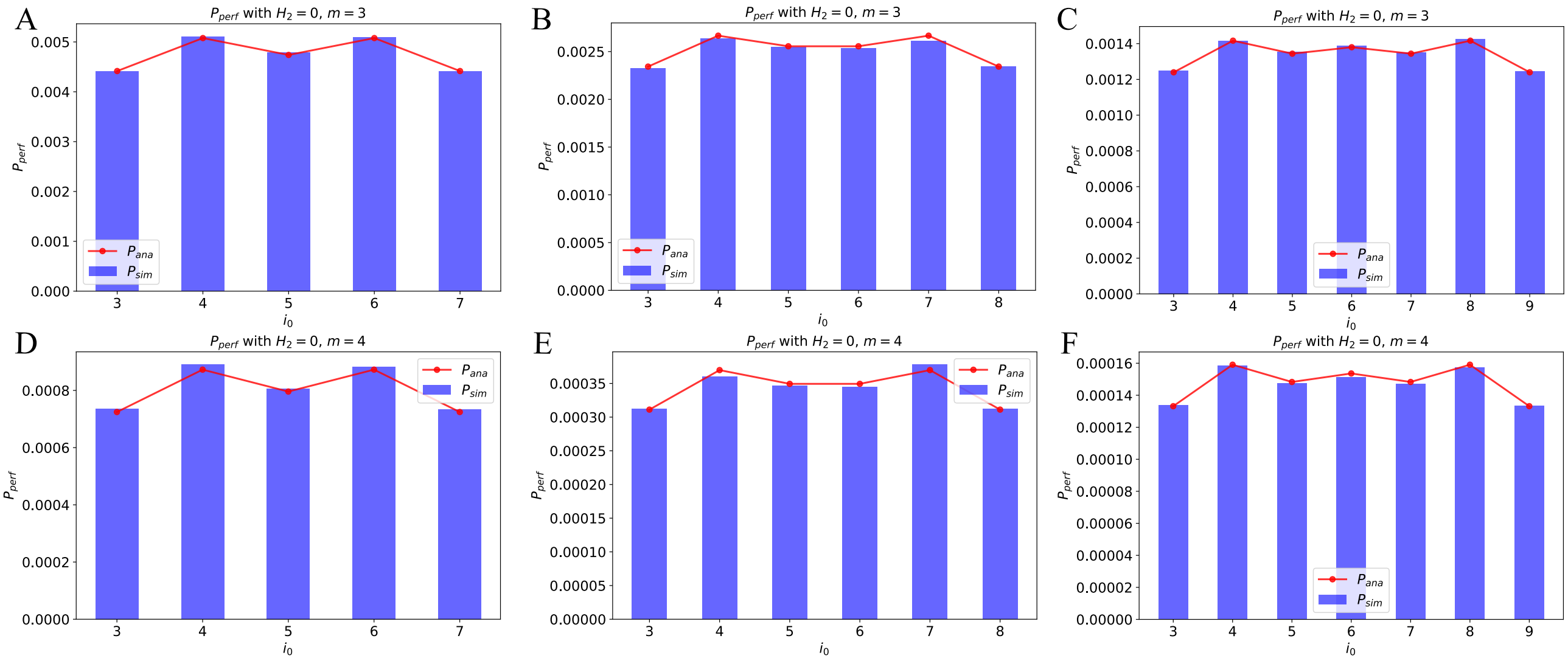

Figure S7. The probability of perfects P_{perf} with respect to the index of non-local link i_0. The probability P_{perf} derived from the Ising Hamiltonian $H_2 = 0$, which is determined by Eq. (80) and obtained by the partition function method. (A)-(C) rely on $n = 8$, $n = 9$, and $n = 10$, with the color number $m = 3$, respectively. (D)-(F) depend on $n = 8$, $n = 9$, and $n = 10$, with the color number $m = 4$, respectively. The simulation results are generated using the Monte Carlo method.

The spin vector $\boldsymbol{s} = [s_1, s_2, \cdots, s_n]^\top$ with $H = 0$ is called perfect. The partition function of H is

$$\begin{aligned} Z &= \sum_{\{S_k\}} e^{-\frac{H_k}{T}} = \sum_{\{S_k\}} \int_{-\infty}^{\infty} dx\, e^{-|x|} \delta(x - \frac{1}{T}\sum_{i,j} a_{ij} s_i s_j) \\ &= \sum_{\{S_k\}} \int_{-\infty}^{\infty} dx \int_{-\infty}^{\infty} \frac{d\hat{x}}{2\pi}\, e^{-|x|} e^{\mathrm{i}\hat{x}\left(x - \frac{\sum_{i,j} a_{ij} s_i s_j}{T}\right)} \\ &= \int_{-\infty}^{\infty} \frac{d\hat{x}}{2\pi} \int_{-\infty}^{\infty} dx\, e^{-|x| + \mathrm{i}\hat{x}x} \sum_{\{S_k\}} e^{-\mathrm{i}\hat{x}\frac{\sum_{i,j} a_{ij} s_i s_j}{T}} \\ &= \int_{-\infty}^{\infty} \frac{d\hat{x}}{\pi} \frac{1}{1+\hat{x}^2} \sum_{\{S_k\}} e^{-\mathrm{i}\hat{x}\frac{\sum_{i,j} a_{ij} s_i s_j}{T}}, \end{aligned} \tag{82}$$

where T is temperature and $\mathrm{i} = \sqrt{-1}$. To solve $\sum_{\{S_k\}} e^{-\mathrm{i}\hat{x}\frac{\sum_{i,j} a_{ij} s_i s_j}{T}}$, we assume $a_{ij} \equiv a$ and define a matrix

$$M = \begin{pmatrix} M_{-1,-1} & M_{-1,1} \\ M_{1,-1} & M_{1,1} \end{pmatrix}, \tag{83}$$

where $M_{p,q} = e^{-\mathrm{i}\hat{x}\frac{a}{T}pq}$ with $p, q \in \{-1, 1\}$. Using $\sum_{\{S_k\}} e^{-\mathrm{i}\hat{x}\frac{\sum_{i,j} a_{ij} s_i s_j}{T}} = \mathrm{Tr}(M^n)$, we rewrite Z as

$$Z = \int_{-\infty}^{\infty} \frac{d\hat{x}}{\pi} \frac{1}{1+\hat{x}^2} (\lambda_1^n + \lambda_2^n), \tag{84}$$

where $\lambda_1 = 2\cos(\frac{a\hat{x}}{T})$ and $\lambda_2 = -2\mathrm{i}\sin(\frac{a\hat{x}}{T})$ are the eigenvalues of M. For the temperature $T \to 0$, we

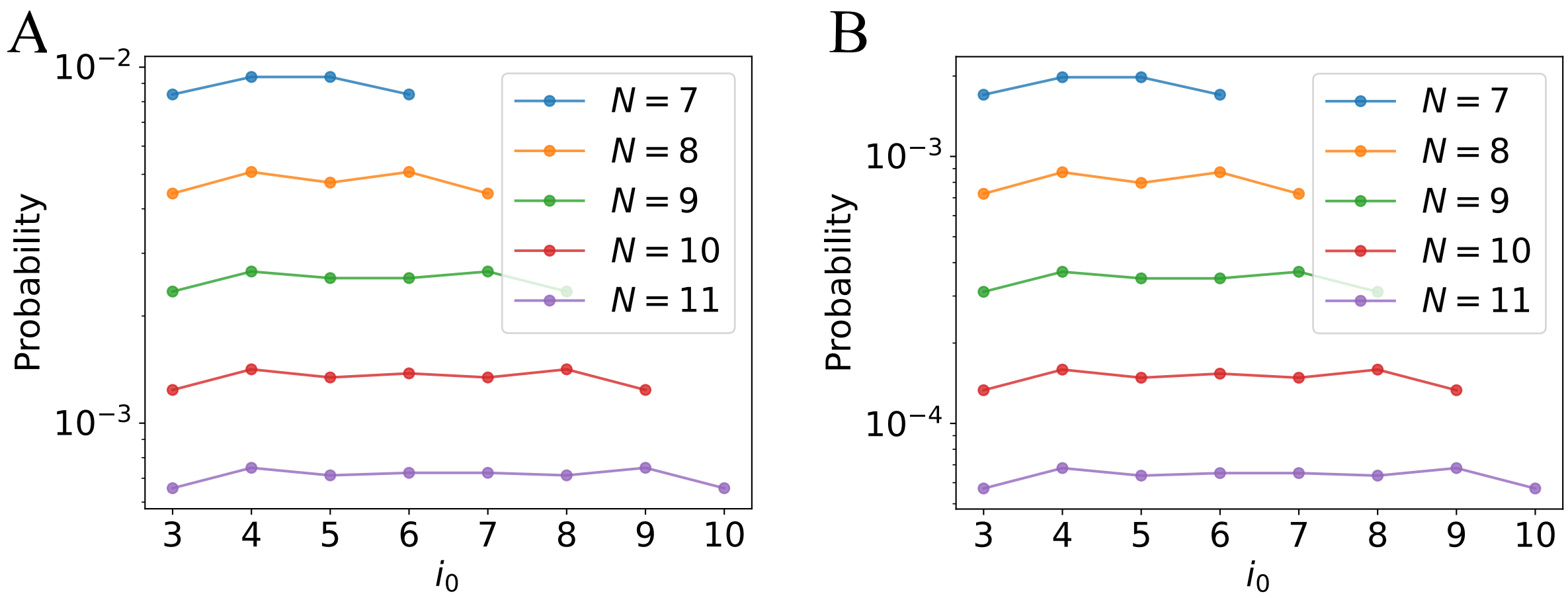

Figure S8. The probability of perfects versus the locations of non-local links. The maximum number of optimal solutions appears when connecting the third neighbors compared to other neighbors. (A) and (B) are associated with the color numbers $m = 3$ and $m = 4$, respectively.

approximate Z by

$$Z = 2^n \int_{-\infty}^{\infty} \frac{d\hat{x}}{\pi} \frac{1}{1+\hat{x}^2} \left[\langle \cos^n(\frac{a\hat{x}}{T}) \rangle + (-\mathrm{i})^n \langle \sin^n(\frac{a\hat{x}}{T}) \rangle \right], \tag{85}$$

where $\langle \cdot \rangle$ denotes the average of periodic function. Using the fact that

$$\frac{1}{2\pi}\int_0^{2\pi} \sin^n(x)dx = \frac{1}{2\pi}\int_0^{2\pi} \cos^n(x)dx = \begin{cases} 0, & n \text{ is odd}, \\ \frac{(n-1)!!}{n!!}, & n \text{ is even}, \end{cases} \tag{86}$$

and $\int_{-\infty}^{\infty} \frac{d\hat{x}}{\pi} \frac{1}{1+\hat{x}^2} = \int_{-\infty}^{\infty} \frac{d(\arctan \hat{x})}{\pi} = 1$, we have

$$Z = \begin{cases} 0, & n = 2n_0 + 1, \\ 0, & n = 2n_0 \text{ and } n_0 > 1, \\ 2^{n+1}\frac{(n-1)!!}{n!!}, & n = 4n_0. \end{cases}, \tag{87}$$

where

$$n!! = \begin{cases} n \times (n-2) \cdots 3 \times 1, & \text{n is odd}, \\ n \times (n-2) \cdots 4 \times 2, & \text{n is even}, \end{cases}, \tag{88}$$

and $n_0 = 1, 2, \cdots$. Eq. (87) describes the number of perfects based on the temperature $T = 0$. We consider a ring with $n = 4n_0$ nodes, where $n_0 = 1, 2, \cdots$. Since the total number of spin configurations is 2^n, the probability of perfects is

$$P_{\text{perf}} = 2\frac{(n-1)!!}{n!!}. \tag{89}$$

Fig. S9 shows that P_{perf} decreases with increasing problem size n.

5.3.2 A ring of n nodes plus one non-local link

Considering a ring of n nodes plus one non-local link, with corresponding Hamiltonian

$$H_b(\boldsymbol{s}) = \left| a \sum_{j=1}^{n} s_j s_{j+1} + b s_1 s_k \right|, \tag{90}$$

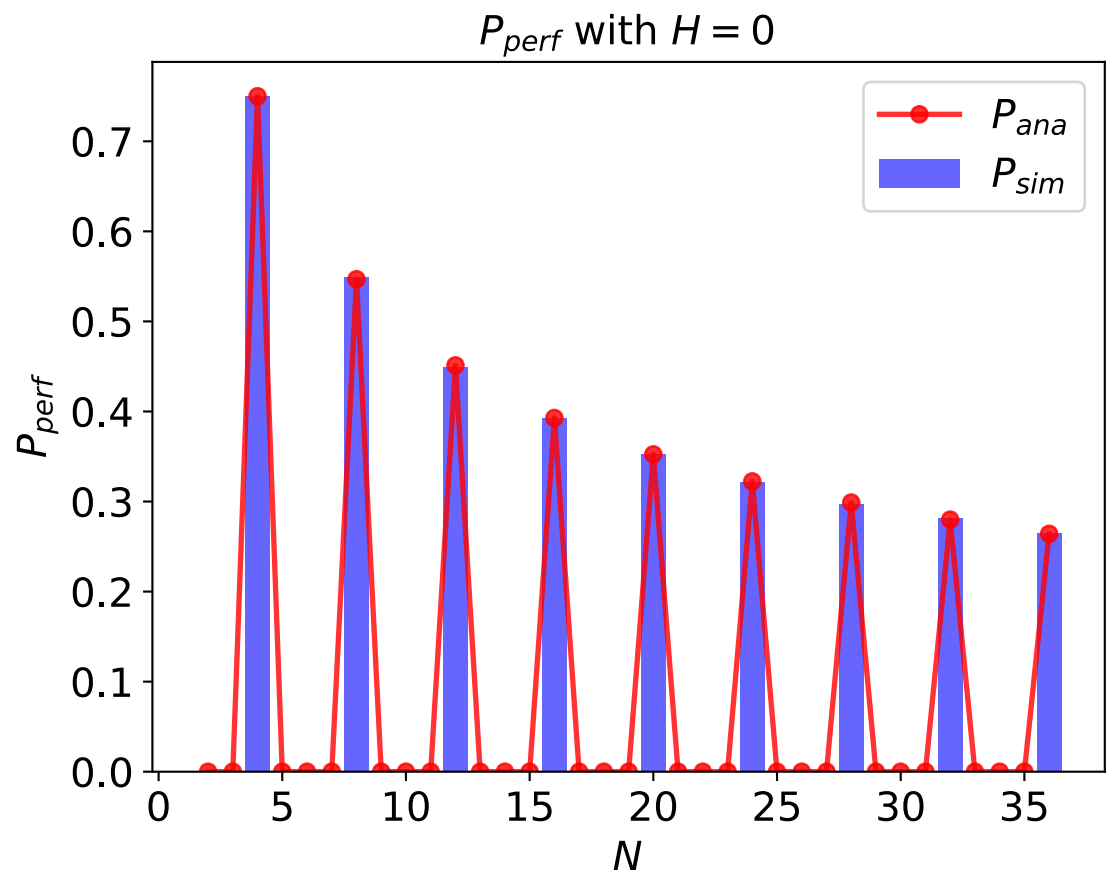

Figure S9. The probability of perfects $P_{\mathbf{perf}}$ decreases with increasing problem size n. The simulation results are obtained by the Monte Carlo method.

where $s_j = \pm 1$, $s_{n+1} \equiv s_1$ and $3 \le k \le n$. The partition function of $H_b(\boldsymbol{s})$ is

$$\begin{aligned} Z &= \sum_{\{S_k\}} e^{-H_b(\boldsymbol{s})} = \sum_{\{S_k\}} \int_{-\infty}^{\infty} dx\, e^{-|x|} \delta\Big(x - \frac{a\sum_{j=1}^{n} s_j s_{j+1} + b s_1 s_k}{T}\Big) \\ &= \sum_{\{S_k\}} \int_{-\infty}^{\infty} dx \int_{-\infty}^{\infty} \frac{d\hat{x}}{2\pi}\, e^{-|x| + \mathrm{i}\hat{x}\left(x - \frac{a\sum_{j=1}^{n} s_j s_{j+1} + b s_1 s_k}{T}\right)} \\ &= \int_{-\infty}^{\infty} \frac{d\hat{x}}{\pi} \frac{1}{1+\hat{x}^2} \sum_{\{S_k\}} e^{-\mathrm{i}\hat{x}\frac{a\sum_{j=1}^{n} s_j s_{j+1} + b s_1 s_k}{T}}. \end{aligned} \tag{91}$$

where we use $\int_{-\infty}^{\infty} dx\, e^{-|x| + \mathrm{i}\hat{x}x} = \frac{2}{1+\hat{x}^2}$. To solve $\sum_{\{S_k\}} e^{-\mathrm{i}\hat{x}\frac{a\sum_{j=1}^{n} s_j s_{j+1} + b s_1 s_k}{T}}$, we use the fact that

$$\begin{aligned} e^{-\mathrm{i}\hat{x}\frac{a\sum_{j=1}^{n} s_j s_{j+1} + b s_1 s_k}{T}} &= e^{-\mathrm{i}\hat{x}\frac{a}{T}s_1 s_2} e^{-\mathrm{i}\hat{x}\frac{a}{T}s_2 s_3} \cdots e^{-\mathrm{i}\hat{x}\frac{a}{T}s_{k-1} s_k} \\ &\times e^{-\mathrm{i}\hat{x}\frac{a}{T}s_k s_{k+1}} \cdots e^{-\mathrm{i}\hat{x}\frac{a}{T}s_{n-1} s_n} e^{-\mathrm{i}\hat{x}\frac{a}{T}s_n s_1} \times e^{-\mathrm{i}\hat{x}\frac{b}{T}s_1 s_k}. \end{aligned} \tag{92}$$

Using the definition of M in Eq. (83), we sum $e^{-\mathrm{i}\hat{x}\frac{a\sum_{j=1}^{n} s_j s_{j+1} + b s_1 s_k}{T}}$ over $s_2, s_3, \cdots, s_{k-1}$ and $s_{k+1}, s_{k+2}, \cdots, s_n$ giving $\left(M^{k-1}\right)_{s_1,s_k} \left(M^{n-(k-1)}\right)_{s_k,s_n} e^{-\mathrm{i}\hat{x}\frac{a}{T}s_1 s_k}$, and obtain

$$\begin{aligned} Z &= \int_{-\infty}^{\infty} \frac{d\hat{x}}{\pi} \frac{1}{1+\hat{x}^2} \sum_{s_1,s_k} \left(M^{k-1}\right)_{s_1,s_k} \left(M^{n-(k-1)}\right)_{s_k,s_n} e^{-\mathrm{i}\hat{x}\frac{b}{T}s_1 s_k} \\ &= \int_{-\infty}^{\infty} \frac{d\hat{x}}{\pi} \frac{1}{1+\hat{x}^2} \left\{ \left[\left(M^{k-1}\right)_{-1,-1} \left(M^{n-(k-1)}\right)_{-1,-1} + \left(M^{k-1}\right)_{1,1} \left(M^{n-(k-1)}\right)_{1,1} \right] e^{-i\hat{x}\frac{b}{T}} \right. \\ &\quad \left. + \left[\left(M^{k-1}\right)_{-1,1} \left(M^{n-(k-1)}\right)_{-1,1} + \left(M^{k-1}\right)_{1,-1} \left(M^{n-(k-1)}\right)_{1,-1} \right] e^{i\hat{x}\frac{b}{T}} \right\}. \end{aligned} \tag{93}$$

The eigen-decomposition of M is

$$M = \begin{pmatrix} \frac{\sqrt{2}}{2} & \frac{\sqrt{2}}{2} \\ \frac{\sqrt{2}}{2} & -\frac{\sqrt{2}}{2}i \end{pmatrix} \begin{pmatrix} \lambda_1 & \\ & \lambda_2 \end{pmatrix} \begin{pmatrix} \frac{\sqrt{2}}{2} & \frac{\sqrt{2}}{2} \\ \frac{\sqrt{2}}{2} & -\frac{\sqrt{2}}{2} \end{pmatrix}, \tag{94}$$

where the two eigenvalues of M are $\lambda_1 = 2\cos(\frac{a\hat{x}}{T})$ and $\lambda_2 = -2\mathrm{i}\sin(\frac{a\hat{x}}{T})$. Using the eigen-decomposition of M, we have

$$M^{k-1} = 2^{k-2}\begin{pmatrix} \cos^{k-1}(\frac{a}{T}\hat{x}) + (-\mathrm{i})^{k-1}\sin^{k-1}(\frac{a}{T}\hat{x}) & \cos^{k-1}(\frac{a}{T}\hat{x}) - (-\mathrm{i})^{k-1}\sin^{k-1}(\frac{a}{T}\hat{x}) \\ \cos^{k-1}(\frac{a}{T}\hat{x}) - (-\mathrm{i})^{k-1}\sin^{k-1}(\frac{a}{T}\hat{x}) & \cos^{k-1}(\frac{a}{T}\hat{x}) + (-\mathrm{i})^{k-1}\sin^{k-1}(\frac{a}{T}\hat{x}) \end{pmatrix}, \tag{95}$$

i.e., $\left(M^{k-1}\right)_{-1,-1} = \left(M^{k-1}\right)_{1,1}$ and $\left(M^{k-1}\right)_{-1,1} = \left(M^{k-1}\right)_{1,-1}$ for any k. We reduce Z to

$$\begin{aligned} Z = &\int_{-\infty}^{\infty}\frac{d\hat{x}}{\pi}\frac{2}{1+\hat{x}^2}\left[\left(M^{k-1}\right)_{1,1}\left(M^{n-(k-1)}\right)_{1,1}e^{-\mathrm{i}\hat{x}\frac{b}{T}} + \left(M^{k}\right)_{1,-1}\left(M^{n-(k-1)}\right)_{1,-1}e^{\mathrm{i}\hat{x}\frac{b}{T}}\right] \\ =&2^n\int_{-\infty}^{\infty}\frac{d\hat{x}}{\pi}\frac{2}{1+\hat{x}^2}\left\{\left[\cos^n(\frac{a}{T}\hat{x}) + (-\mathrm{i})^n\sin^n(\frac{a}{T}\hat{x})\right]\cos(\frac{b}{T}\hat{x})\right. \\ &\left. -\,\mathrm{i}\left[(-\mathrm{i})^{n-(k-1)}\cos^{k-1}(\frac{a}{T}\hat{x})\sin^{n-(k-1)}(\frac{a}{T}\hat{x}) + (-\mathrm{i})^{k-1}\cos^{n-(k-1)}(\frac{a}{T}\hat{x})\sin^{k-1}(\frac{a}{T}\hat{x})\right]\sin(\frac{b}{T}\hat{x})\right\} \end{aligned} \tag{96}$$

The actual result depends on whether n, k, and $n-k$ are separately even or odd, and the relation of b to a.

For a ring plus one non-local link, assuming b is an integer multiple of a and the perfect is existed, only if $n+\frac{b}{a}$ is even. We consider the number of perfects which is obtained from the limitation $T \to 0$ in Eq. (96). Using the De Moivre's formula $(\cos x + \mathrm{i}\sin x)^n = \cos(nx) + \mathrm{i}\sin(nx)$, we have

$$\begin{aligned} \cos(nx) + \mathrm{i}\sin(nx) =& \sum_{j=0}^{n}\frac{n!}{j!(n-j)!}\mathrm{i}^j\sin^j(x)cos^{n-j}(x) \\ =&\sum_{j=0}^{\lfloor\frac{n}{2}\rfloor}\frac{(-1)^j n!}{(2j)!(n-2j)!}\sin^{2j}(x)\cos^{n-2j}(x) \\ &+\mathrm{i}\sum_{j=0}^{\lfloor\frac{n}{2}\rfloor}\frac{(-1)^j n!}{(2j+1)!(n-2j-1)!}\sin^{2j+1}(x)\cos^{n-2j-1}(x), \end{aligned} \tag{97}$$

where $\lfloor\cdot\rfloor$ is the floor function.Using the fact that b is an integer multiple of a, we obtain

$$\cos(\frac{b}{T}\hat{x}) = \sum_{p=0}^{\lfloor\frac{b}{2a}\rfloor}\frac{(-1)^p(\frac{b}{a})!}{(2p)!(\frac{b}{a}-2p)!}\sin^{2p}(\frac{a}{T}\hat{x})\cos^{\frac{b}{a}-2p}(\frac{a}{T}\hat{x}), \tag{98}$$

$$\sin(\frac{b}{T}\hat{x}) = \sum_{p=0}^{\lfloor\frac{b-a}{2a}\rfloor}\frac{(-1)^p(\frac{b}{a})!}{(2p+1)!(\frac{b}{a}-2p-1)!}\sin^{2p+1}(\frac{a}{T}\hat{x})\cos^{\frac{b}{a}-2p-1}(\frac{a}{T}\hat{x}). \tag{99}$$

Substituting Eqs. (98) and (99) into the partition function Eq. (96), and using $n+\frac{b}{a}$ is even, we can approximate Z with $T \to 0$ to obtain the number of perfects. Due to $P_{\mathrm{perf}} = Z/2^n$, we calculate the probability of perfects in the following cases.

Case 1 n is odd, $\frac{b}{a}$ is odd.

(i) k is odd.

$$\begin{aligned} P_{\mathrm{perf}} =& \sum_{p=0}^{\lfloor\frac{b}{2a}\rfloor}\frac{(-1)^p(\frac{b}{a})!}{(2p)!(\frac{b}{a}-2p)!}\langle\sin^{2p}(\frac{a}{T}\hat{x})\cos^{\frac{b}{a}+n-2p}(\frac{a}{T}\hat{x})\rangle \\ &+\sum_{p=0}^{\lfloor\frac{b-a}{2a}\rfloor}\frac{(-1)^p(\frac{b}{a})!}{(2p+1)!(\frac{b}{a}-2p-1)!}(-1)^{\frac{n-k+2}{2}}\langle\sin^{2p+1+n-k+1}(\frac{a}{T}\hat{x})\cos^{\frac{b}{a}-2p-1+k-1}(\frac{a}{T}\hat{x})\rangle. \end{aligned} \tag{100}$$

Using the fact that

$$\frac{1}{2\pi}\int_0^{2\pi}\cos^{n_1}x\sin^{n_2}(x)dx=\begin{cases}\frac{(n_1-1)!!}{(n_2+1)(n_2+3)\cdots(n_2+n_1-1)}\frac{(n_2+n_1-1)!!}{(n_2+n_1)!!}, & \text{both } n_1 \text{ and } n_2 \text{ are even,}\\ 0, & \text{else,}\end{cases} \tag{101}$$

we obtain

$$\begin{aligned}P_{\rm perf}=&\sum_{p=0}^{\lfloor\frac{b}{2a}\rfloor}\frac{(-1)^p(\frac{b}{a})!}{(2p)!(\frac{b}{a}-2p)!}\frac{(\frac{b}{a}-2p+n-1)!!}{(2p+1)(2p+3)\cdots(\frac{b}{a}+n-1)}\frac{(\frac{b}{a}+n-1)!!}{(\frac{b}{a}+n)!!}\\&+\sum_{p=0}^{\lfloor\frac{b-a}{2a}\rfloor}\frac{(-1)^{p+\frac{n-k+2}{2}}(\frac{b}{a})!}{(2p+1)!(\frac{b}{a}-2p-1)!}\frac{(\frac{b}{a}-2p+k-3)!!}{(2p+n-k+3)(2p+n-k+5)\cdots(\frac{b}{a}+n-1)}\frac{(\frac{b}{a}+n-1)!!}{(\frac{b}{a}+n)!!}.\end{aligned} \tag{102}$$

(ii) k is even.

$$\begin{aligned}P_{\rm perf}=&\sum_{p=0}^{\lfloor\frac{b}{2a}\rfloor}\frac{(-1)^p(\frac{b}{a})!}{(2p)!(\frac{b}{a}-2p)!}\langle\sin^{2p}(\frac{a}{T}\hat{x})\cos^{\frac{b}{a}+n-2p}(\frac{a}{T}\hat{x})\rangle\\&+\sum_{p=0}^{\lfloor\frac{b-a}{2a}\rfloor}\frac{(-1)^p(\frac{b}{a})!}{(2p+1)!(\frac{b}{a}-2p-1)!}(-1)^{\frac{k}{2}}\langle\sin^{2p+k}(\frac{a}{T}\hat{x})\cos^{\frac{b}{a}+n-2p-k}(\frac{a}{T}\hat{x})\rangle\\=&\sum_{p=0}^{\lfloor\frac{b}{2a}\rfloor}\frac{(-1)^p(\frac{b}{a})!}{(2p)!(\frac{b}{a}-2p)!}\frac{(\frac{b}{a}-2p+n-1)!!}{(2p+1)(2p+3)\cdots(\frac{b}{a}+n-1)}\frac{(\frac{b}{a}+n-1)!!}{(\frac{b}{a}+n)!!}\\&+\sum_{p=0}^{\lfloor\frac{b-a}{2a}\rfloor}\frac{(-1)^{p+\frac{k}{2}}(\frac{b}{a})!}{(2p+1)!(\frac{b}{a}-2p-1)!}\frac{(\frac{b}{a}+n-2p-k-1)!!}{(2p+k+1)(2p+k+3)\cdots(\frac{b}{a}+n-1)}\frac{(\frac{b}{a}+n-1)!!}{(\frac{b}{a}+n)!!}\end{aligned} \tag{103}$$

For the case where n is odd and b/a is odd, the variations of $P_{\rm perf}$ versus the non-local link k is displayed in Fig. S10 (A)-(C).

Case 2 n is even, $\frac{b}{a}$ is even.

(i) k is odd.

$$\begin{aligned}P_{\rm perf}=&\sum_{p=0}^{\lfloor\frac{b}{2a}\rfloor}\frac{(-1)^p(\frac{b}{a})!}{(2p)!(\frac{b}{a}-2p)!}\left[\langle\sin^{2p}(\frac{a}{T}\hat{x})\cos^{\frac{b}{a}+n-2p}(\frac{a}{T}\hat{x})\rangle+(-1)^{\frac{n}{2}}\langle\sin^{2p+n}(\frac{a}{T}\hat{x})\cos^{\frac{b}{a}-2p}(\frac{a}{T}\hat{x})\rangle\right]\\=&\sum_{p=0}^{\lfloor\frac{b}{2a}\rfloor}\frac{(-1)^p(\frac{b}{a})!}{(2p)!(\frac{b}{a}-2p)!}\left[\frac{(\frac{b}{a}-2p+n-1)!!}{(2p+1)(2p+3)\cdots(\frac{b}{a}+n-1)}\frac{(\frac{b}{a}+n-1)!!}{(\frac{b}{a}+n)!!}\right.\\&\left.+(-1)^{\frac{n}{2}}\frac{(\frac{b}{a}-2p-1)!!}{(2p+n+1)(2p+n+3)\cdots(\frac{b}{a}+n-1)}\frac{(\frac{b}{a}+n-1)!!}{(\frac{b}{a}+n)!!}\right].\end{aligned} \tag{104}$$

Remarkably, Z is independent of k.

(ii) k is even.

$$
\begin{aligned}
P_{\rm perf} =& \sum_{p=0}^{\lfloor \frac{b}{2a} \rfloor} \frac{(-1)^p (\frac{b}{a})!}{(2p)!(\frac{b}{a}-2p)!} \Big[\langle \sin^{2p}(\frac{a}{T}\hat{x}) \cos^{\frac{b}{a}+n-2p}(\frac{a}{T}\hat{x}) \rangle + (-1)^{\frac{n}{2}} \langle \sin^{2p+n}(\frac{a}{T}\hat{x}) \cos^{\frac{b}{a}-2p}(\frac{a}{T}\hat{x}) \rangle \Big] \\
&+ \sum_{p=0}^{\lfloor \frac{b-a}{2a} \rfloor} \frac{(-1)^p (\frac{b}{a})!}{(2p+1)!(\frac{b}{a}-2p-1)!} \Big[(-1)^{\frac{n-k+2}{2}} \langle \sin^{2p+n-k+2}(\frac{a}{T}\hat{x}) \cos^{\frac{b}{a}+k-2p-2}(\frac{a}{T}\hat{x}) \rangle \\
&+ (-1)^{\frac{k}{2}} \langle \sin^{2p+k}(\frac{a}{T}\hat{x}) \cos^{\frac{b}{a}+n-2p-k}(\frac{a}{T}\hat{x}) \rangle \Big] \\
=& \Bigg\{ \sum_{p=0}^{\lfloor \frac{b}{2a} \rfloor} \frac{(-1)^p (\frac{b}{a})!}{(2p)!(\frac{b}{a}-2p)!} \Bigg[\frac{(\frac{b}{a}-2p+n-1)!!}{(2p+1)(2p+3)\cdots(\frac{b}{a}+n-1)} \\
&+ (-1)^{\frac{n}{2}} \frac{(\frac{b}{a}-2p-1)!!}{(2p+n+1)(2p+n+3)\cdots(\frac{b}{a}+n-1)} \Bigg] \\
&+ \sum_{p=0}^{\lfloor \frac{b-a}{2a} \rfloor} \frac{(-1)^p (\frac{b}{a})!}{(2p+1)!(\frac{b}{a}-2p-1)!} \Bigg[(-1)^{\frac{n-k+2}{2}} \frac{(\frac{b}{a}+k-2p-3)!!}{(2p+n-k+3)(2p+n-k+5)\cdots(\frac{b}{a}+n-1)} \\
&+ (-1)^{\frac{k}{2}} \frac{(\frac{b}{a}+n-k-2p-1)!!}{(2p+k+1)(2p+k+3)\cdots(\frac{b}{a}+n-1)} \Bigg] \Bigg\} \frac{(\frac{b}{a}+n-1)!!}{(\frac{b}{a}+n)!!}.
\end{aligned}
\tag{105}
$$

For the case where both n and b/a are even, the variations of $P_{\rm perf}$ versus the non-local link k is shown in Fig. S10 (D)-(E). In particular, the probability $P_{\rm perf} \equiv 0$ if n is integral multiple of 4, i.e., $n = 4n_0$ with $n_0 = 0, 1, 2, \cdots$ (see Fig. S11).

6 Solving graph coloring problem

6.1 From Ising Hamiltonian to Kuramoto-type oscillator model

We expand the sum of H in Eq. (64),

$$
\begin{aligned}
H =& \sum_{i=1}^{n} \left[(m-2)^2 + \sum_{k=1}^{m} s_{ic_k}^2 + 2(m-2)\sum_{k=1}^{m} s_{ic_k} + \sum_{k=1}^{m} s_{ic_k} \sum_{l\neq k} s_{ic_l} \right] \\
&+ \sum_{(i,j)\in\mathbb{E}}^{N_{\mathbb{E}}} \sum_{k=1}^{m} (1 + s_{ic_k} + s_{jc_k} + s_{ic_k}s_{jc_k}).
\end{aligned}
\tag{106}
$$

Using the fact that $s_{ic_k}^2 = 1$, we have

$$
\begin{aligned}
H =& n(m-2)^2 + nm + N_{\mathbb{E}}m + 2(m-2)\sum_{i=1}^{n}\sum_{k=1}^{m} s_{ic_k} + \sum_{i=1}^{n}\sum_{k=1}^{m}\sum_{l\neq k} s_{ic_k}s_{ic_l} \\
&+ \sum_{(i,j)\in\mathbb{E}}^{N_{\mathbb{E}}} \sum_{k=1}^{m} (s_{ic_k} + s_{jc_k} + s_{ic_k}s_{jc_k}),
\end{aligned}
\tag{107}
$$

where $\sum_{l\neq k} s_{ic_k}s_{ic_l}$ contains $2s_{ic_k}s_{ic_l}$ for any $k \neq l$, $\sum_{(i,j)\in\mathbb{E}}^{N_{\mathbb{E}}} s_{ic_k}$ and $\sum_{(i,j)\in\mathbb{E}}^{N_{\mathbb{E}}} s_{jc_k}$ are connected to the degree of node i and j, respectively. The Ising Hamiltonian H incorporates products of two spins, represented by J_{ij}s,

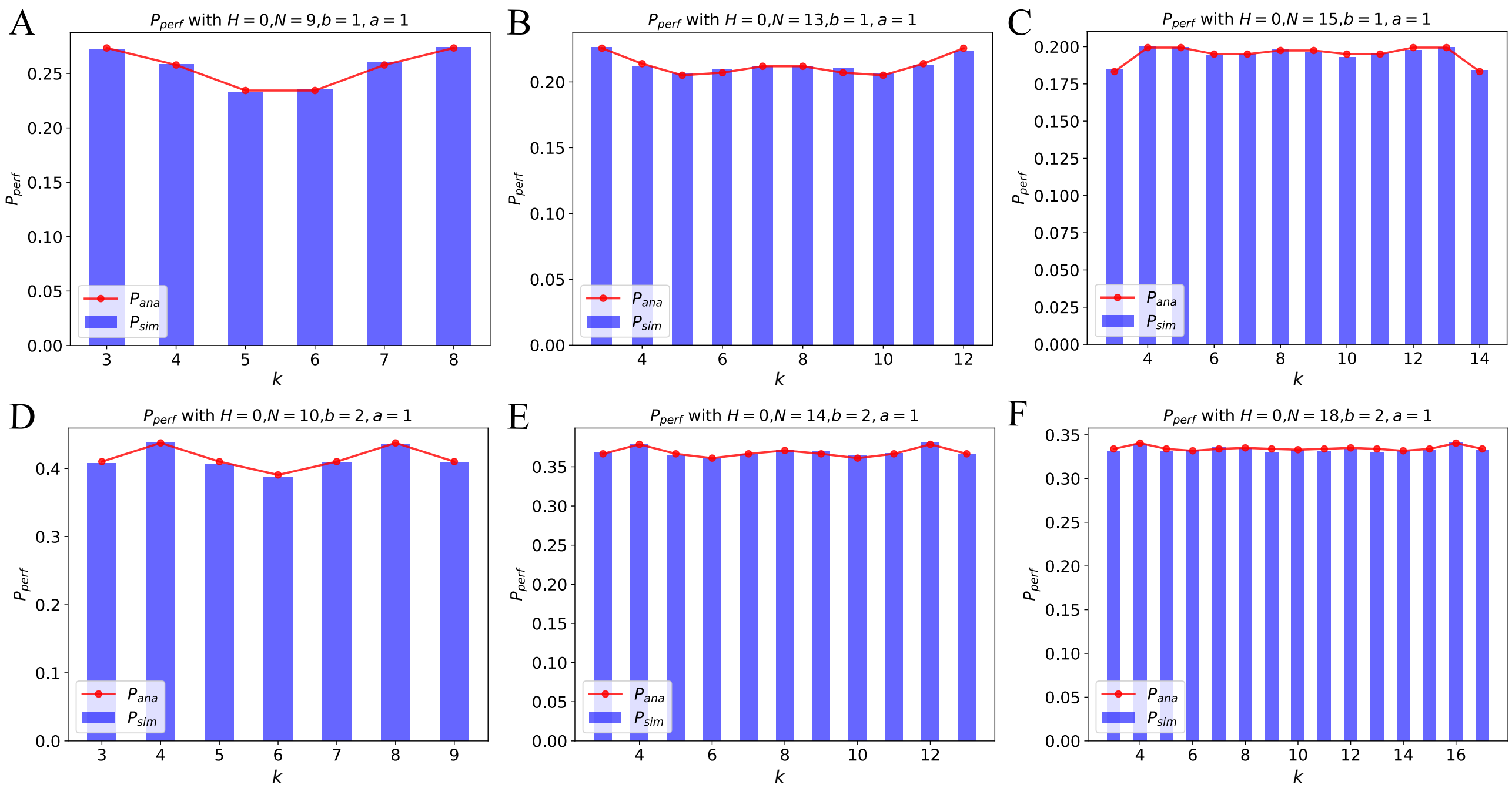

Figure S10. The probability of perfects $P_{\mathbf{perf}}$ versus the index of non-local link k. (A)-(C) Both n and b/a are odd, with n =9, 13, 15, respectively. The weight of non-local link is $b = 1$, and other connection weights are $a = 1$. (D)-(E) Both n and b/a are even, with n =10, 14, 18, respectively. The weight of non-local link is $b = 2$, and other connection weights are $a = 1$. The simulation results are obtained by the Monte Carlo method.

self-terms represented by h_i, and the square of spins. We label the spins s_{ic_k} such that $s_{ic_k} = s_{i+(k-1)n}$ for $i = 1, \cdots, n$, and obtain the dynamics of oscillator i

$$\dot{\phi}_i = -Kh_i \sin\phi_i - K\sum_j J_{ij}\sin(\phi_i - \phi_j) - K_s \sin(2\phi_i), \tag{108}$$

for $i = 1, 2, \cdots, nm$. As the Kuramoto oscillators converge to stationary states with the Ising Hamiltonian $H = 0$, the choice of $\phi_{ic_k} = 0$ corresponds to coloring the i-th node with the color c_k.

6.2 Parameter settings of the Kuramoto-type oscillator model

Coloring a graph with n nodes using m colors, the expansion of H_1 is

$$\begin{aligned} H_1 =& \sum_{i=1}^{n}\left[(m-2)^2 + 2(m-2)\sum_{k=1}^{m} s_{ic_k} + \sum_{k=1}^{m} s_{ic_k}^2 + \sum_{k=1}^{m}\sum_{l\neq k} s_{ic_k}s_{ic_l}\right] \\ =& n(m-2)^2 + nm + 2(m-2)\sum_{i=1}^{n}\sum_{k=1}^{m} s_{ic_k} + \sum_{i=1}^{n}\sum_{k=1}^{m}\sum_{l\neq k} s_{ic_k}s_{ic_l}, \end{aligned} \tag{109}$$

where the term $\sum_{i=1}^{n}\sum_{k=1}^{m}\sum_{l\neq k} s_{ic_k}s_{ic_l}$ denotes the couplings between different colors for the same region. If we use J to represent the coupling matrix of spins with the number $N = nm$, the off-diagonal block of J is $-I$, where I is the identity matrix.

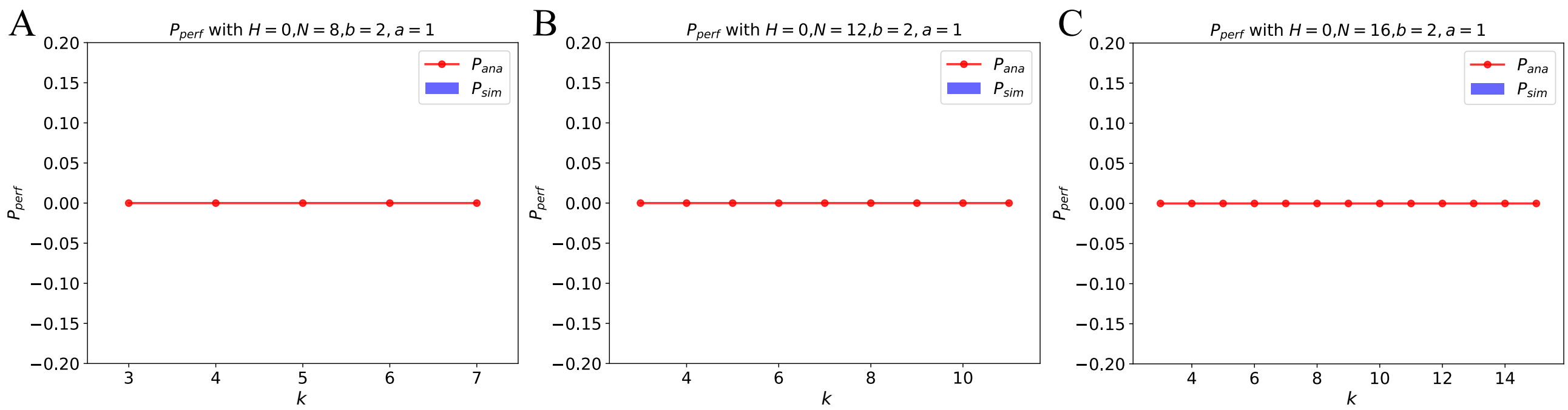

Figure S11. When n is integral multiple of 4, i.e., $n = 4n_0$ with $n_0 = 0, 1, 2, \cdots$, and b/a are even, the probability $P_{\mathbf{perf}} \equiv 0$ with the variations of non-local link index k. (A)-(C) illustrate scenarios with n =8, 12, 16, respectively, relying on $a = 1$ and $b = 2$. The simulation results are obtained using the Monte Carlo method.

Expanding H_2 as

$$\begin{aligned} H_2 =& \sum_{k=1}^{m} \sum_{(i,j)\in\mathbb{E}}^{N_\mathbb{E}} (1 + s_{ic_k} + s_{jc_k} + s_{ic_k}s_{jc_k}) \\ =& N_\mathbb{E}m + \sum_{k=1}^{m}\left[\sum_{(i,j)\in\mathbb{E}} s_{ic_k} + \sum_{(i,j)\in\mathbb{E}} s_{jc_k}\right] + \sum_{k=1}^{m}\sum_{(i,j)\in\mathbb{E}} s_{ic_k}s_{jc_k}. \end{aligned} \tag{110}$$

Denoting $\sum_{i=1}^{n} d_i s_{ic_k} = \sum_{(i,j)\in\mathbb{E}} s_{ic_k}$, $\sum_{j=1}^{n} d_j s_{jc_k} = \sum_{(i,j)\in\mathbb{E}} s_{jc_k}$, where d_i and d_j represent the degrees of i-th and j-th nodes, respectively.

$$\begin{aligned} H_2 =& N_\mathbb{E}m + \sum_{k=1}^{m}\left[\sum_{(i,j)\in\mathbb{E}} d_i s_{ic_k} + \sum_{(i,j)\in\mathbb{E}} d_j s_{jc_k}\right] + \sum_{k=1}^{m}\sum_{(i,j)\in\mathbb{E}} s_{ic_k}s_{jc_k} \\ =& N_\mathbb{E}m + 2\sum_{k=1}^{m}\sum_{(i,j)\in\mathbb{E}} d_i s_{ic_k} + \sum_{k=1}^{m}\sum_{(i,j)\in\mathbb{E}} s_{ic_k}s_{jc_k}. \end{aligned} \tag{111}$$

The term $\sum_{k=1}^{m}\sum_{(i,j)\in\mathbb{E}} s_{ic_k}s_{jc_k}$ denotes the couplings between different regions for same color, resulting in the diagonal block of J as $-A$, where A is the adjacency matrix of the graph to be colored. From the derivations of H_1 and H_2, the self-term h_i of spins is expressed as $h_i = -(m-2) - d_i$.

To validate the efficacy of the Kuramoto-type oscillator model in solving the graph coloring problem, we employ two representative networks, namely a ring and a ring plus one non-local link, which is used in the partition function method. We extract the matrix J and array h from the two networks and simulate the Kuramoto-type oscillator model described by Eq. (108). In order to facilitate the system (108) in escaping local optimal solutions and prompting the discovery of minimal values of the Ising Hamiltonian, Gaussian white noise is injected into the system. Fig. S12 (A)-(C) depict the coloring schedule and phase evolution of a ring, and panels (D)-(E) illustrate the corresponding results for a ring plus one non-local link.

6.3 Coloring a map

The graph coloring problem is often exemplified by the map coloring problem, where regions (represented by nodes) on a map need to be colored such that no two adjacent regions share the same color. The four color

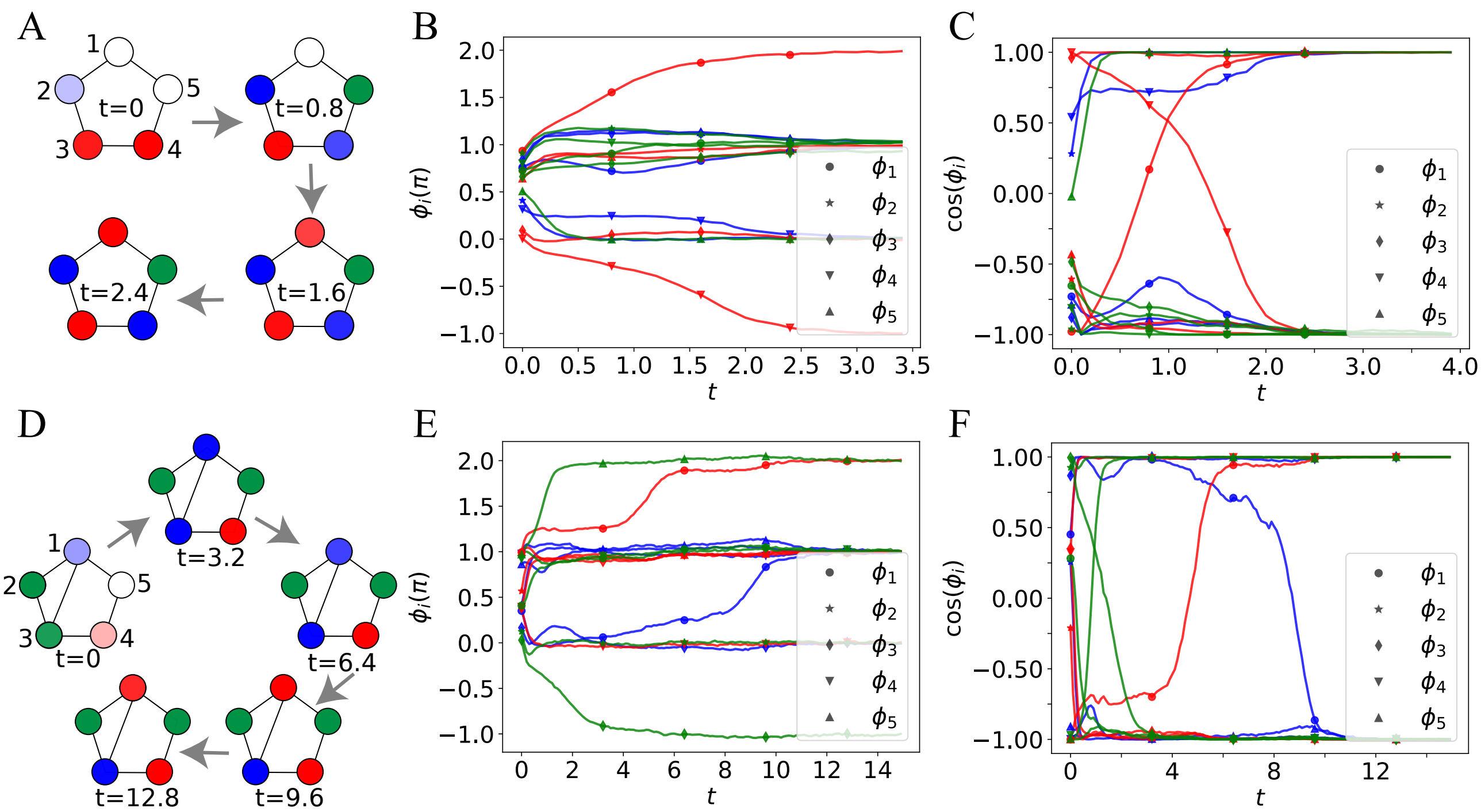

Figure S12. The Kuramoto-type oscillator model effectively solves the graph coloring problem. (A)-(C) The results of a ring with 5 nodes. (A) The coloring schedule is displayed at four time points $t = 0$, 0.8, 1.6, 2.4. (B) The evolution of phases with varying time t. The markers at four time points correspond to those in (A). Each type of marker represents a specific node, and the associated three lines represent three colors: red, blue, and green. (C) The evolution of $\cos(\phi_i)$. The meanings of markers and colors are consistent with (B). Each node in (A) is colored by the corresponding marker, with phases satisfying $\cos(\phi_i) = 1$. (D)-(F) The results of a ring plus one non-local link. (D) The coloring schedule is shown at five time points $t = 0$, 3.2, 6.4, 9.6, 12.8. (E) The phase evolution of the Kuramoto oscillators. (F) The evolution of $\cos(\phi_i)$.

map theorem ensures that any plane map can be colored using four colors [11,12]. We extract a simplified map with $n = 47$ regions from the American mainland map shown in Fig. S13 (A), and employ the Kuramoto-type oscillator model to solve the problem via $m = 4$ colors.

The Kuramoto oscillators exhibit oscillations between different states in the early time stages, approaching stationary states after a short duration (refer to Fig. S13 (B)). Concurrently, the corresponding Ising spins $s_i = \cos(\phi)$ tend to a balanced state as illustrated in Fig. S13 (C). Fig. S13 (D) demonstrates that the system converges to a state associated with minimal energy values. The Ising Hamiltonian progressively approaches 0 (Fig. S13 (E)), indicating that the system is reaching a solution where the energy is minimized. The decreasing Ising Hamiltonian indicates that the system approaches a state in which neighboring regions have distinct colors. The Ising Hamiltonian ultimately attains 0, signifying that the system successfully finds a valid solution to the map-coloring problem (see Fig. S13 (F)).

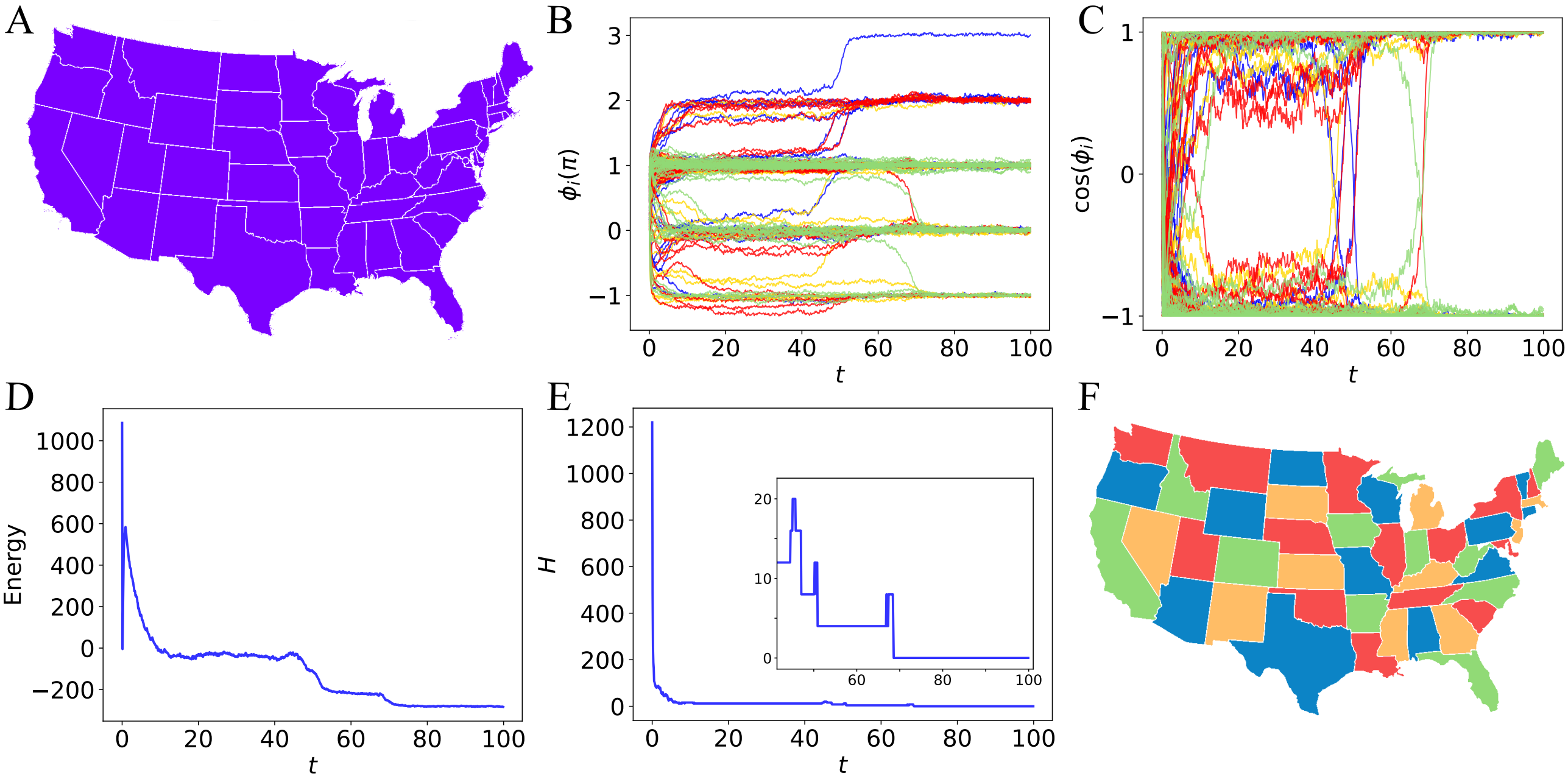

Figure S13. Coloring the American mainland map using the Kuramoto-type oscillator model. (A) A non-colored American mainland map. (B) Phase evolution of the Kuramoto oscillators. (C) The evolution of $\cos(\phi_i)$. (D) The variations of energy versus time. (E) The Ising Hamiltonian decreases and eventually approaches 0 with respect to time. (F) The colored American mainland map, where neighboring regions have different colors, reflecting a successful solution of the map coloring problem.

7 Solving MaxCut problem

7.1 Kuramoto-type oscillator model

One of the applications of the Kuramoto-type oscillator model is finding the maximum cut of a graph. Let $\mathcal{G} = (\mathcal{V}, \mathcal{E})$ be a graph with vertices $\mathcal{V}$ and edges $\mathcal{E}$. A cut of the graph $\mathcal{G}$ is a partition of the vertices of $\mathcal{G}$ into two disjoint subsets $\mathcal{G}_1 = (\mathcal{V}_1, \mathcal{E}_1)$, $\mathcal{G}_2 = (\mathcal{V}_2, \mathcal{E}_2)$, with $\mathcal{V}_1 \cap \mathcal{V}_2 = \varnothing$. The size of the cut is defined to be the number of edges connecting the two subsets. The maximum cut is defined to be the cut of a graph $\mathcal{G}$ whose size is at least as large as any other cut. For a weighted graph object, we can define the maximum cut to be the cut with weight at least as large as any other cut. The problem of finding a maximum cut in a graph is known as the MaxCut problem, which is one of the 21 algorithms on Karp's 21 NP-complete problems [1, 13].

The MaxCut problem can be mapped to the Ising model as follows. Given a graph $\mathcal{G} = (\mathcal{V}, \mathcal{E})$, with edge weight w_{ij} between vertices i and j. Choosing J_{ij} to be the negative of the weight of the edges between vertices i and j, i.e., $J_{ij} = -w_{ij}$, and partitioning the vertices into two sets $\mathcal{V}_1$ and $\mathcal{V}_2$. Therefore, there are three groups of edges in the graph $\mathcal{G}$, i.e., the edges connecting vertices within $\mathcal{V}_1$, the edges connecting vertices within $\mathcal{V}_2$, and the cut set containing edges across $\mathcal{V}_1$ and $\mathcal{V}_2$. The sums of the weights in these three sets are denoted by S_1, S_2, and S_{cut}, respectively, and together constitute the total weight of the graph $\mathcal{G}$, i.e.,

$$S_1 + S_2 + S_{\text{cut}} = \sum_{i<j} w_{ij} = -\sum_{i<j} J_{ij}. \tag{112}$$

This partitioning directly corresponds to the values of Ising spins, achieved by assigning $+1$ to a spin i if $i \in \mathcal{V}_1$

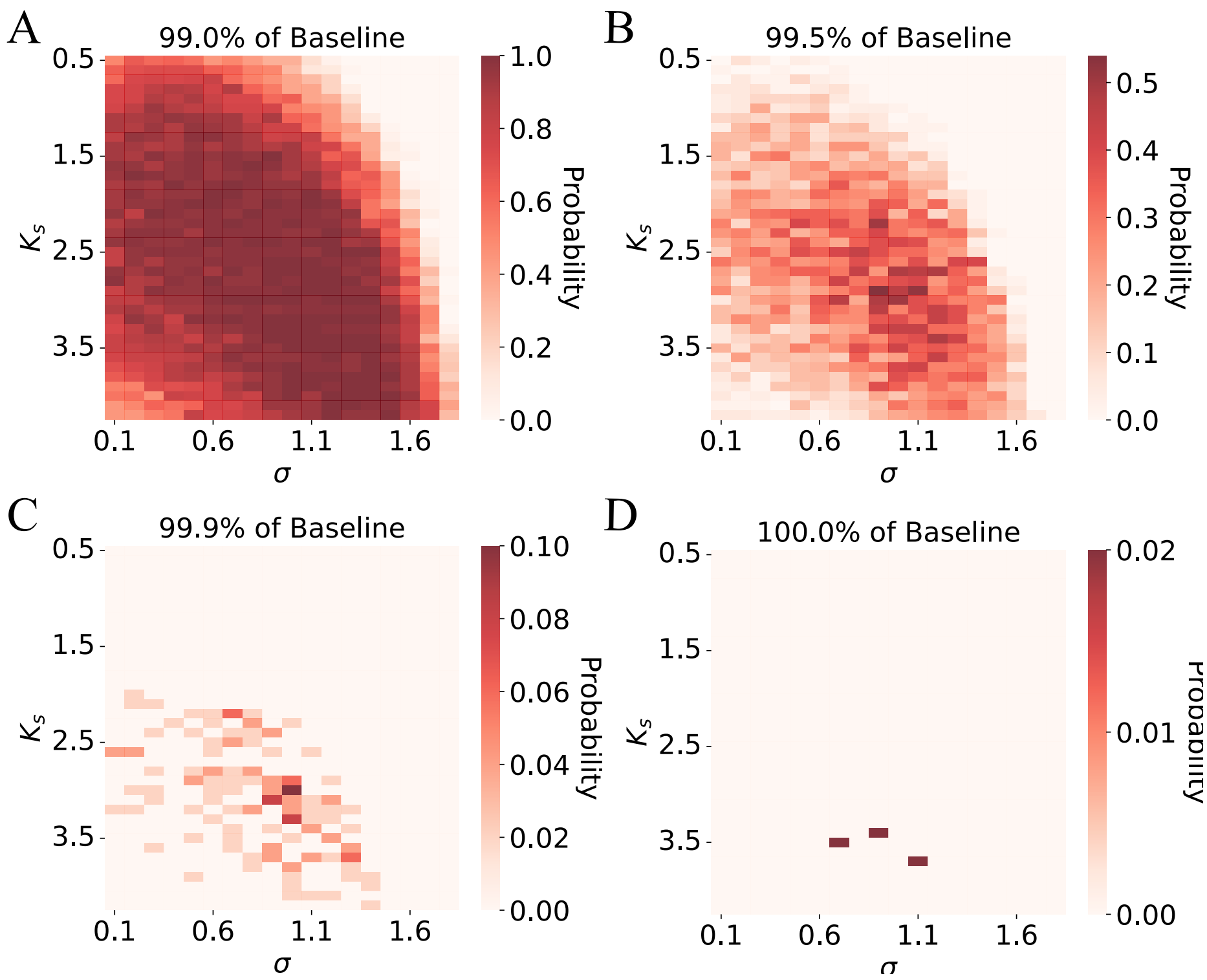

Figure S14. The probabilities of different percentages of baseline obtained by the Kuramoto-type oscillator model. (A) Probability distribution for 99% of the baseline. (B) Probability distribution for 99.5% of the baseline. (C) Probability distribution for 99.9% of the baseline. (D) Probability distribution for 100% of the baseline. The graph is obtained from G1 set, with 800 nodes and 19176 edges. The Kuramoto-type oscillator model is simulated 50 times and the global coupling strength is fixed at $K = 1.5$. The parameter ranges are $K_s \in [0.5, 4.2]$ and $\sigma \in [0.1, 1.8]$, both sampled with an interval of 0.1. The initial phases are randomly distributed uniformly in the interval $[0, 2\pi)$. The time t of phase evolution is 100s, with a time step of 0.004s. The cut value is obtained by substituting the phases into Eq. (114) and using $s_i = \cos\phi_i$. The Gaussian white noise satisfies mean 0 and standard deviation $\sqrt{2}\sigma$. The baseline value, 11624, is the best-known cut value of G1.

and -1 if $i \in \mathcal{V}_2$. The Ising Hamiltonian in Eq. (18) can be rewritten as

$$\begin{aligned}
H &= -\sum_{i<j} J_{ij} s_i s_j \\
&= -\sum_{i<j,\, i,j\in\mathcal{V}_1} J_{ij}(+1)(+1) - \sum_{i<j,\, i,j\in\mathcal{V}_2} J_{ij}(-1)(-1) - \sum_{i<j,\, i\in\mathcal{V}_1,\, j\in\mathcal{V}_2} J_{ij}(+1)(-1) \\
&= -\sum_{i<j,\, i,j\in\mathcal{V}_1} J_{ij} - \sum_{i<j,\, i,j\in\mathcal{V}_2} J_{ij} + \sum_{i<j,\, i\in\mathcal{V}_1,\, j\in\mathcal{V}_2} J_{ij} \\
&= S_1 + S_2 - S_{\text{cut}} \\
&= (S_1 + S_2 + S_{\text{cut}}) - 2S_{\text{cut}} \\
&= \sum_{i<j} w_{ij} - 2S_{\text{cut}}. \qquad (113) \\
&= -\sum_{i<j} J_{ij} - 2S_{\text{cut}}
\end{aligned}$$

Therefore, the maximal value of cut size is corresponding to the minimal value of Ising Hamiltonian. The maximal cut value is

$$\begin{aligned}S_{\text{cut}} &= -\frac{1}{2}(H + \sum_{i<j} J_{ij}) \\ &= \frac{1}{2}\sum_{i<j} J_{ij}(s_i s_j - 1) \\ &= \frac{1}{2}\sum_{i<j} w_{ij}(1 - s_i s_j).\end{aligned} \tag{114}$$

To apply the Kuramoto-type oscillator model to the MaxCut problem, Eq. (9) is rewritten as

$$\dot{\phi}_i(t) = -K\sum_{j\neq i}^{n} J_{ij}\sin(\phi_i(t) - \phi_j(t)) - K_s\sin(2\phi_i(t)) + \eta_i(t), \tag{115}$$

where $\eta_i(t)$ represents the Gaussian white noise defined in Eq. (46).

Using a graph $\mathcal{G}$ generated from G1, comprising 800 nodes and 19176 edges, we simulate the Kuramoto-type oscillator model described by Eq. (115) to solve the MaxCut problem. The results reveal a maximal cut value of 11624. To provide a comprehensive comparison, we evaluate the Kuramoto-type oscillator model against three alternative methods which will be described in detail as follows. The Kuramoto-type oscillator model reaches the best-known cut value of 11624 and exhibits competitive performance relative to the other methods. We use the best-known cut value of G1, 11624, as the reference value (baseline) [1], and establish the probability distribution for different percentages relative to this baseline. For the Kuramoto-type oscillator model with varying K_s and σ, Fig. S14 (A)-(D) show the probability distribution for 99%, 99.5%, 99.9%, 100% of the baseline. In particular, the higher probabilities mainly concentrate in the region with $K_s > \sigma$ for all four percentages.

7.2 OEO-based CIMs

To evaluate the performance of the Kuramoto-type oscillator model for solving MaxCut problems, we compare it with several alternative optimization methods. The first comparison method is an OEO-based coherent Ising machine (CIM) with self-feedback [14]. The discrete-time dynamics of the i-th oscillator is described by

$$x_i[k+1] = \cos^2(f_i[k] - \frac{\pi}{4} + \zeta_i[k]) - \frac{1}{2}, \tag{116}$$

where $x_i[k]$ denotes the state of the i-th oscillator at iteration k, and $\zeta_i[k]$ denotes Gaussian noise with zero mean and standard deviation σ. The constant phase bias $-\pi/4$ shifts the nonlinear transfer function, while the feedback signal $f_i[k]$ determines the input to the nonlinear map. The feedback signal is defined as

$$f_i[k] = \alpha x_i[k] + \beta\sum_j J_{ij}x_j[k], \tag{117}$$

where α controls the self-feedback of each oscillator, β controls the coupling strength among oscillators, and J_{ij} denotes the Ising coupling matrix. The second term incorporates the interactions with the other oscillator states according to the prescribed Ising coupling topology.

The oscillator state is mapped to the corresponding Ising spin through $s_i = \text{sgn}(x_i[k])$, where $\text{sgn}(x)$ denotes the sign function. Thus, the sign of $x_i[k]$ determines the binary spin state used to evaluate the Ising Hamiltonian and the corresponding MaxCut value. For the graph $\mathcal{G}$ from the G1 instance, we employ the OEO-based CIM described by Eqs. (116) and (117) to solve the MaxCut problem. The maximal cut value obtained is 11623. With varying α and β, Fig. S15(A)–(D) shows the probability distributions for 99%, 99.5%, 99.9%, and 100% of the baseline.

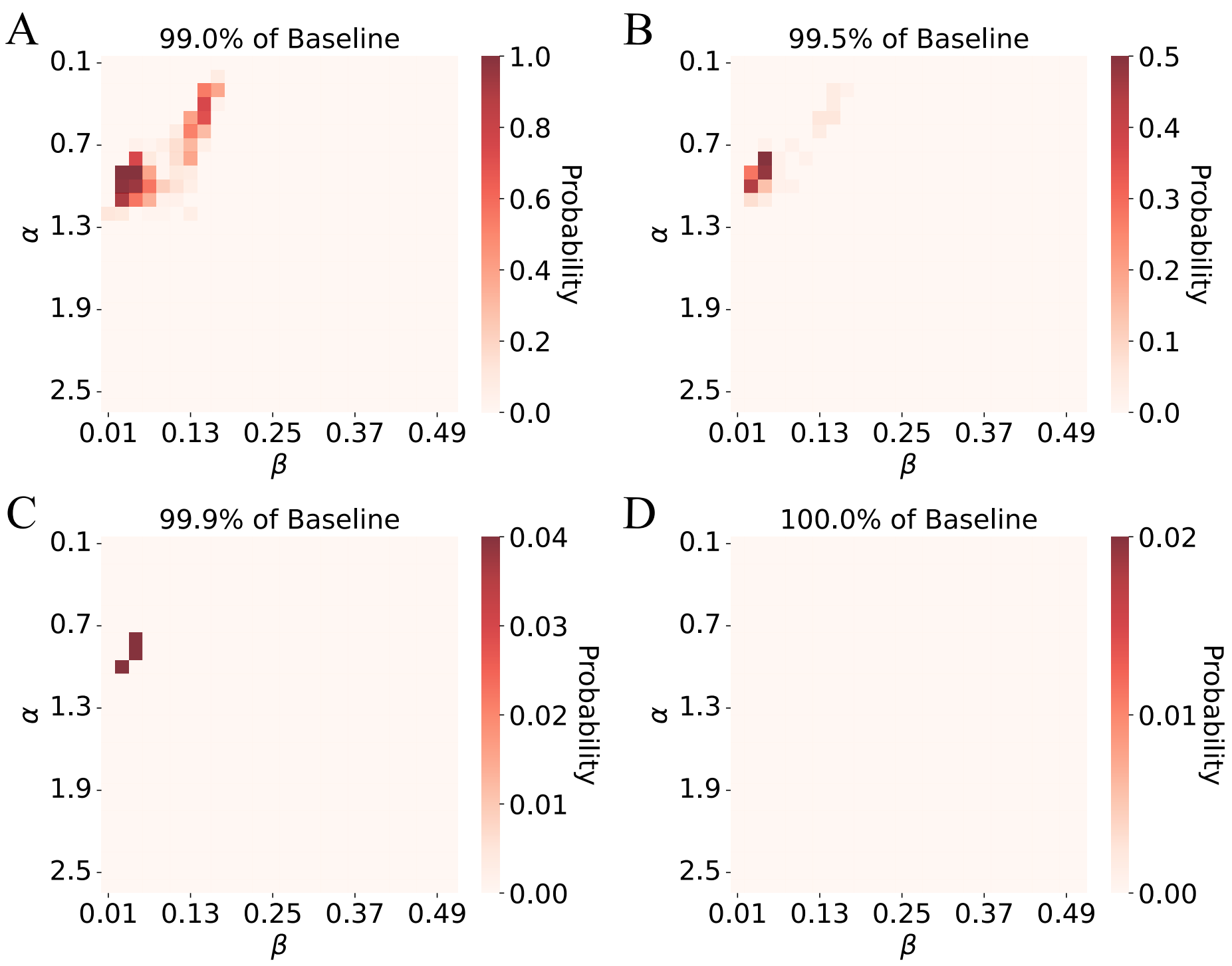

Figure S15. Probabilities of different percentages of the baseline obtained by OEO-based CIMs. (A) Probability distribution for 99% of the baseline. (B) Probability distribution for 99.5% of the baseline. (C) Probability distribution for 99.9% of the baseline. (D) Probability distribution for 100% of the baseline. The OEO-based CIM is simulated 50 times, with all oscillator states initialized at $x_i = 0$. Each simulation consists of 5000 iterations. The standard deviation of Gaussian noise ζ_i is fixed at 0.1. The cut values are obtained by substituting the states into Eq. (114) with $s_i = \mathrm{sgn}(x_i)$.

7.3 Monte Carlo simulations

As another comparison method, we employ simulated annealing based on single-spin Metropolis updates to solve the MaxCut problem. At each update, one Ising spin is selected according to a sequential typewriter ordering, and the corresponding change in the Ising energy, ΔH, is evaluated. A proposed flip is accepted with the Metropolis probability

$$P_{\mathrm{acc}} = \min\left[1, \exp\left(-\frac{\Delta H}{k_B T}\right)\right], \tag{118}$$

where k_B is the Boltzmann constant and T denotes the temperature. Energy-decreasing moves are always accepted, whereas energy-increasing moves are accepted with a temperature-dependent probability. The temperature controls the exploration of the configuration space, with higher temperatures allowing occasional unfavorable moves and lower temperatures progressively favoring lower-energy states.

For the G1 instance, the spins are initialized independently with $s_i = \pm 1$ with equal probability. We set the Boltzmann constant $k_B = 1$, and the temperature follows the exponential annealing schedule [14]

$$T[k] = 2\exp(-0.0001k), \tag{119}$$

where k denotes the index of the single-spin update. Each simulation consists of 300000 single-spin update attempts, and the largest cut value reached over the entire trajectory is recorded. We perform 50 independent

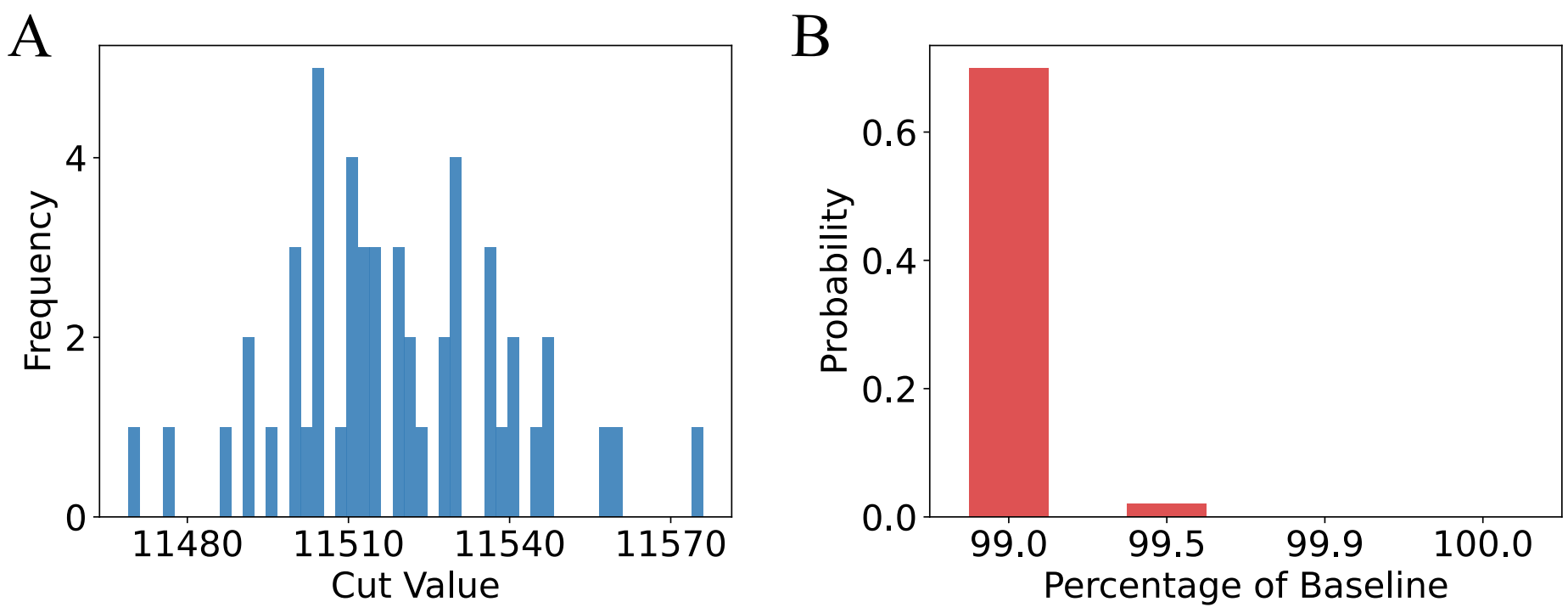

Figure S16. Cut values and probabilities relative to the baseline obtained by Monte Carlo simulations. (A) The best cut values obtained from 50 independent Monte Carlo simulations. (B) Probabilities of reaching 99.0%, 99.5%, 99.9%, and 100% of the baseline. Each simulation consists of 300000 single-spin update attempts. The spins are initialized independently with $s_i = \pm 1$ with equal probability and are updated sequentially in a typewriter ordering. The best cut value reached over the entire trajectory is recorded for each simulation.

simulations. The maximal cut value obtained is 11576. Figure S16(A) shows the distribution of the best cut values obtained from the 50 simulations, while Fig. S16(B) shows the probabilities of reaching 99%, 99.5%, 99.9%, and 100% of the baseline.

7.4 D-Wave NetworkX

As another comparison method, we solve the MaxCut problem using D-Wave NetworkX with the classical simulated annealing sampler provided by the D-Wave Ocean tools (https://dnx.readthedocs.io). The graph structure is constructed from the G1 adjacency matrix, and the `maximum_cut` function returns a set of nodes defining one of the two graph partitions. The corresponding cut value is calculated from the total weight of the edges connecting the two partitions.

For each independent run, the simulated annealing sampler is configured with one read and 1000 sweeps. We perform 50 independent runs using different random seeds. The maximal cut value obtained is 11624. Figure S17(A) shows the distribution of the cut values obtained from the 50 runs, while Fig. S17(B) shows the probabilities of reaching 99%, 99.5%, 99.9%, and 100% of the baseline.

References

[1] Tianshi Wang and Jaijeet Roychowdhury. Oim: Oscillator-based ising machines for solving combinatorial optimisation problems. In *International Conference on Unconventional Computation and Natural Computation*, pages 232–256. Springer, 2019.

[2] Shigeru Shinomoto and Yoshiki Kuramoto. Phase transitions in active rotator systems. *Progress of Theoretical Physics*, 75(5):1105–1110, 1986.

[3] Aleksandr Mikhailovich Lyapunov. The general problem of the stability of motion. *International journal of control*, 55(3):531–534, 1992.

[4] Tianshi Wang, Leon Wu, Parth Nobel, and Jaijeet Roychowdhury. Solving combinatorial optimisation problems using oscillator based ising machines. *Natural Computing*, 20(2):287–306, 2021.

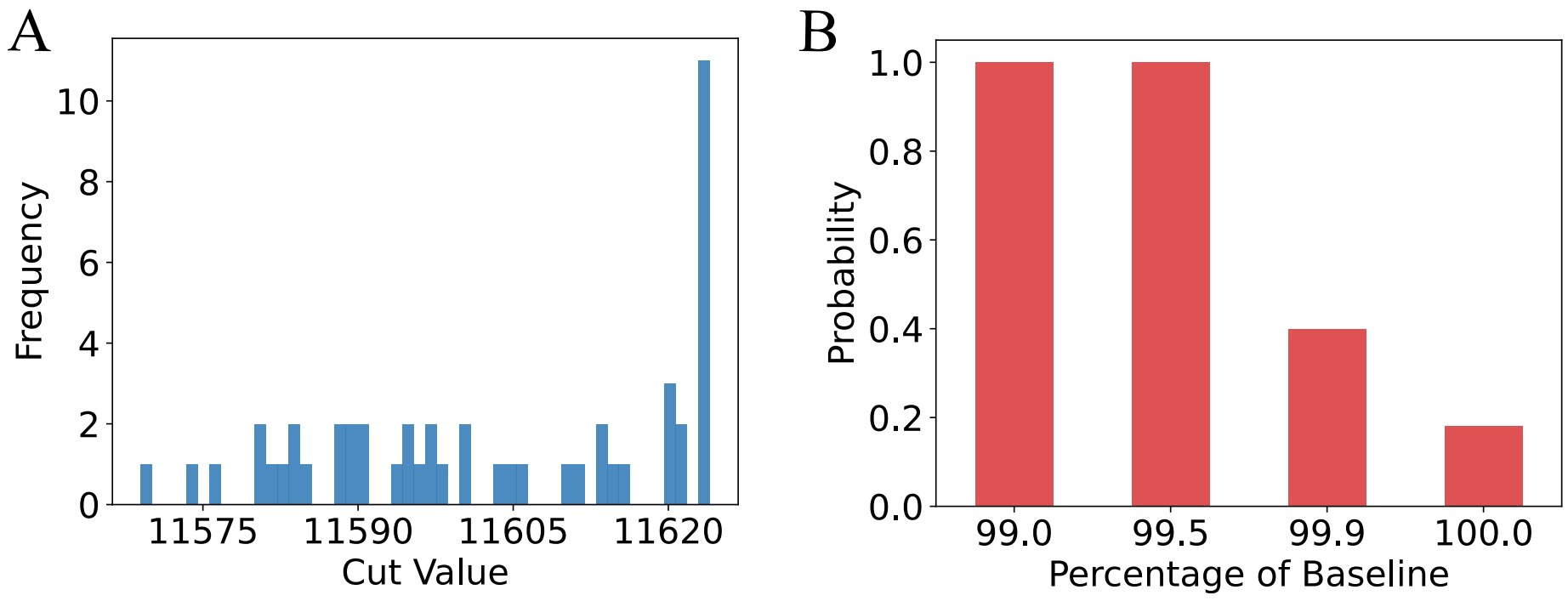

Figure S17. Cut values and probabilities relative to the baseline obtained using D-Wave NetworkX. (A) Cut values obtained from 50 independent runs of D-Wave NetworkX with the simulated annealing sampler. (B) Probabilities of reaching 99.0%, 99.5%, 99.9%, and 100% of the baseline. For each run, the sampler uses one read and 1000 sweeps.

[5] Seon Hee Park and Seunghwan Kim. Noise-induced phase transitions in globally coupled active rotators. *Physical Review E*, 53(4):3425, 1996.

[6] Seunghwan Kim, Seon Hee Park, Charles R Doering, and Chang Su Ryu. Reentrant transitions in globally coupled active rotators with multiplicative and additive noises. *Physics Letters A*, 224(3):147–153, 1997.

[7] Ernst Ising. Beitrag zur theorie des ferromagnetismus. *Zeitschrift für Physik*, 31(1):253–258, 1925.

[8] Edward Laurence, Nicolas Doyon, Louis J Dubé, and Patrick Desrosiers. Spectral dimension reduction of complex dynamical networks. *Physical Review X*, 9(1):011042, 2019.

[9] Bala Kameshwar Poolla, Saverio Bolognani, and Florian Dörfler. Placing rotational inertia in power grids. In *2016 American Control Conference (ACC)*, pages 2314–2320. IEEE, 2016.

[10] Hannes Risken. Fokker-planck equation. In *The Fokker-Planck Equation*, pages 63–95. Springer, 1996.

[11] Rudolf Fritsch, Rudolf Fritsch, G Fritsch, and Gerda Fritsch. *Four-Color Theorem*. Springer, 1998.

[12] Georges Gonthier et al. Formal proof–the four-color theorem. *Notices of the AMS*, 55(11):1382–1393, 2008.

[13] Richard M Karp. Reducibility among combinatorial problems. In *Complexity of Computer Computations: Proceedings of a symposium on the Complexity of Computer Computations, held March 20–22, 1972, at the IBM Thomas J. Watson Research Center, Yorktown Heights, New York, and sponsored by the Office of Naval Research, Mathematics Program, IBM World Trade Corporation, and the IBM Research Mathematical Sciences Department*, pages 85–103. Springer, 1972.

[14] Fabian Böhm, Guy Verschaffelt, and Guy Van der Sande. A poor man's coherent ising machine based on opto-electronic feedback systems for solving optimization problems. *Nature communications*, 10(1):1–9, 2019.
